# CCDC134 controls TLR biogenesis through the ER chaperone Gp96

**DOI:** 10.1084/jem.20240825

**Published:** 2024-12-10

**Authors:** Léa Bernaleau, Michaela Drobek, Fenja Blank, Philipp Walch, Maeva Delacrétaz, Ales Drobek, Marta Monguió-Tortajada, Petr Broz, Olivia Majer, Manuele Rebsamen

**Affiliations:** 1 Department of Immunobiology, University of Lausanne, Epalinges, Switzerland; 2 Max Planck Institute for Infection Biology, Berlin, Germany

## Abstract

Toll-like receptors (TLRs) are central to initiate immune responses against invading pathogens. To ensure host defense while avoiding aberrant activation leading to pathogenic inflammation and autoimmune diseases, TLRs are tightly controlled by multilevel regulatory mechanisms. Through a loss-of-function genetic screen in a reporter cell line engineered to undergo cell death upon TLR7-induced IRF5 activation, we identified here CCDC134 as an essential factor for TLR responses. CCDC134 deficiency impaired endolysosomal TLR-induced NF-κB, MAPK, and IRF5 activation, as well as downstream production of proinflammatory cytokines and type I interferons. We further demonstrated that CCDC134 is an endoplasmic reticulum (ER)–resident interactor of Gp96 (HSP90B1/Grp94), an ER chaperone essential for folding and trafficking of plasma membrane and endolysosomal TLRs. CCDC134 controlled Gp96 stability as its loss led to Gp96 hyperglycosylation and ER-associated protein degradation (ERAD)-mediated clearance. Accordingly, CCDC134 deficiency impaired the folding, maturation, and trafficking of TLRs, resulting in blunted inflammatory responses upon stimulation. Altogether, this study reveals CCDC134 as a central regulator of the chaperone Gp96, thereby controlling TLR biogenesis and responses.

## Teaser

A loss-of-function genetic screen identifies the ER-resident protein CCDC134 as an essential regulator of Toll-like receptor (TLR) responses. CCDC134 binds and stabilizes the TLR chaperone Gp96, controlling thereby the folding and trafficking of the plasma membrane and endolysosomal TLRs.

## Graphical Abstract

**Figure:**
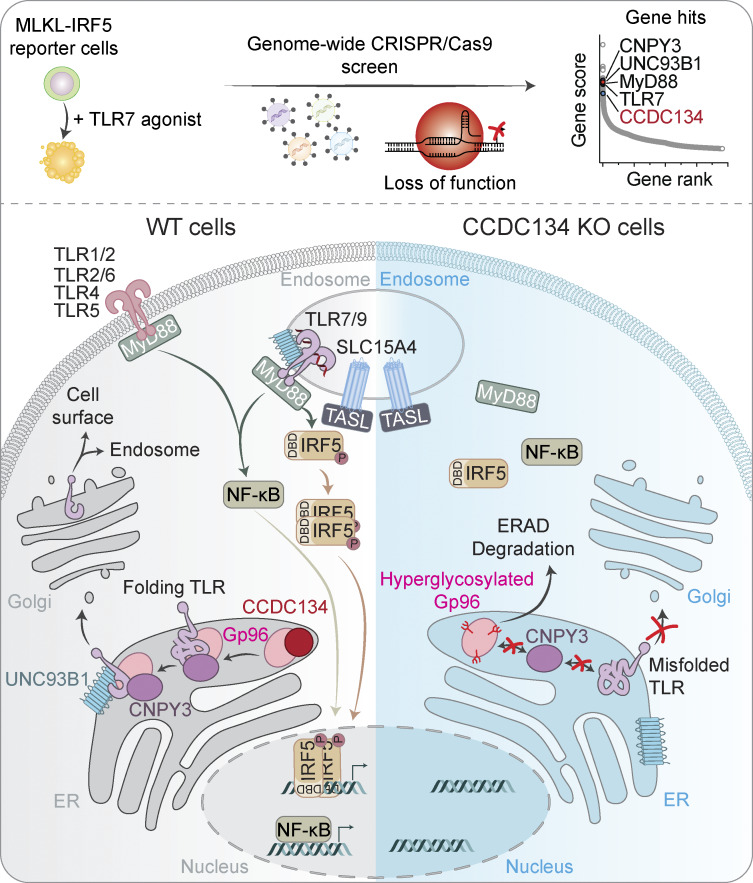

## Introduction

Toll-like receptors (TLRs) are critical innate sensors required to initiate immune responses upon pathogen invasion or tissue damage (Fitzgerald and Kagan, 2020; Kawai et al., 2024; Lind et al., 2022). While these responses are central for host protection, TLRs need to be tightly regulated, as uncontrolled activation can contribute to the development of inflammatory and autoimmune diseases (Lind et al., 2022; Pelka et al., 2016; Vinuesa et al., 2023).

TLRs are type I transmembrane receptors composed of an extracellular leucine-rich repeat domain, which detects the ligand, a single transmembrane domain, and an intracellular Toll/IL-1 receptor domain, which ensures downstream signal transduction (Fitzgerald and Kagan, 2020; Kawai et al., 2024; Lind et al., 2022). Ten (1–10) and twelve (1–9, 11–13) TLRs are expressed in humans and mice, respectively. TLRs are first synthesized in the endoplasmic reticulum (ER), where they need to be properly folded to traffic to their final location on the cell surface (TLR1–2, 4–6, 10) or in the endolysosomal compartment (TLR3, 7–9, 11–13). Folding and export from the ER are controlled by several ER proteins. For instance, folding of TLRs 1, 2, 4, 5, 7, and 9 has been shown to be mediated by the chaperone Gp96 (HSP90B1/Grp94/endoplasmin), a member of the HSP90 family controlling the folding of a limited number of client proteins including some integrins, and by its TLR-specific co-chaperone CNPY3 (PRAT4A) (Liu et al., 2010; Randow and Seed, 2001; Takahashi et al., 2007; Wakabayashi et al., 2006; Yang et al., 2007). Furthermore, UNC93B1 is required for the trafficking of TLR5 to the cell surface and of intracellular TLRs to the endolysosomal compartments (Huh et al., 2014; Majer et al., 2017). Of note, besides trafficking, UNC93B1 also regulates endosomal TLRs’ activation and degradation (Majer et al., 2019a, 2019b). In the endosomal compartment, TLR7, 8, and 9 undergo further proteolytic cleavage in their ectodomain, an additional key step for signaling (Majer et al., 2017; Miyake et al., 2021). While cell surface TLRs recognize mainly microbial components, endosomal TLRs detect nucleic acids (NA) and their degradation products (de Oliveira Mann and Hornung, 2021). NA sensing allows broader recognition of pathogens but increases the risk of aberrant activation by self-molecules (Bartok and Hartmann, 2020). Supporting this, human and mouse studies have linked inappropriate endosomal TLRs’ activation with autoinflammatory and autoimmune disorders, including systemic lupus erythematosus (Pelka et al., 2016; Vinuesa et al., 2023). Engagement of TLR7-9 by the respective ligands results in the recruitment of the adaptor protein MyD88, followed by the induction of the NF-κB, MAPK, and interferon regulatory factor (IRF) pathways leading to the production of proinflammatory cytokines and type I interferons (IFN) (Kawai et al., 2024; Lind et al., 2022). Among the IRF transcription factors, IRF5 is a key mediator for both of these responses (Almuttaqi and Udalova, 2019; Ban et al., 2018). Upon TLR7-9 activation, IRF5 is recruited to the recently identified SLC15A4-TASL endolysosomal complex, resulting in its phosphorylation, dimerization, and translocation to the nucleus where it contributes to these transcriptional responses (Boeszoermenyi et al., 2023; Chen et al., 2023; Heinz et al., 2020; Zhang et al., 2023a). Given the established involvement of the TLR7-9 pathway as well as of IRF5 in inflammatory and autoimmune disorders (Almuttaqi and Udalova, 2019; Ban et al., 2018; Pelka et al., 2016; Vinuesa et al., 2023), we set up to investigate the regulatory network controlling this signaling pathway.

By performing a genome-scale loss-of-function screen on TLR7 responses using an IRF5-dependent reporter system allowing positive selection, we identified here the previously poorly characterized protein CCDC134 as an ER-resident component critically required for both endolysosomal and plasma membrane TLR responses. Mechanistically, CCDC134 interacts with the chaperone Gp96 in the ER and controls its stability, with loss of CCDC134 resulting in Gp96 hyperglycosylation and degradation. Accordingly, CCDC134 deficiency affected the folding, stability, and trafficking of multiple TLRs. These findings uncover CCDC134 as a critical regulator of Gp96 required for TLRs’ biogenesis and possibly other Gp96-dependent pathways.

## Results

### Genome-scale loss-of-function screen for TLR7-dependent IRF5 activation using an MLKL-IRF5 chimera reporter system identified CCDC134

**Figure 1.**
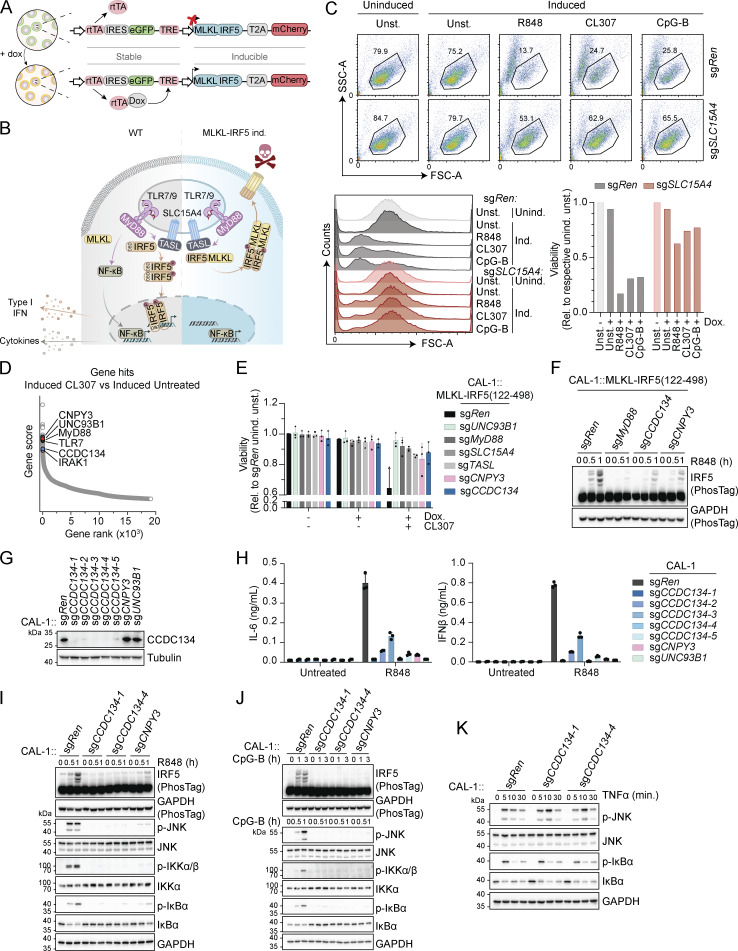
**A genome-wide loss-of-function screen identified CCDC134 as an essential factor for TLR7/9 signaling. (A)** Schematic of doxycycline-inducible MLKL-IRF5(122–498)-T2A-mCherry construct. **(B)** Schematic of TLR7-9 signaling in wildtype cells versus MLKL-IRF5(122–498)-T2A-mCherry expressing cells. **(C)** Representative dot-plot of FSC versus SSC gating used to assess cell viability (upper panel), with histogram for FSC and quantification of cell viability relative to the respective uninduced unstimulated control condition (lower panels). CAL-1 cells stably expressing MLKL-IRF5(122–498)-T2A-mCherry construct (CAL-1 reporter clone) and carrying sgRNA targeting *SLC15A4* (sg*SLC15A4*) or control sgRNA targeting *Renilla* (sg*Ren*) were induced or not with doxycycline (0.5 µg/ml) for 17 h and stimulated with R848 (2 µg/ml), CL307 (2 µg/ml) or CpG-B (ODN2006, 2 µM) for 6 h. unind.: uninduced; ind.: induced; unst.: unstimulated; Dox.: doxycycline. **(D)** Results of genome-wide loss-of-function screen in CAL-1 reporter cells (clone). Gene rank and gene score based on comparison between doxycycline-induced CL307 treated versus doxycycline-induced untreated conditions. **(E)** Cell viability of the indicated CAL-1 reporter cells assessed by flow cytometry (based on FSC versus SSC gating), relative to sg*Ren* uninduced unstimulated (unind. unst.). Cells were induced or not with doxycycline (Dox.) (0.5 µg/ml, 17 h) before being stimulated or not with CL307 (2 µg/ml, 6 h). **(F)** Immunoblots of indicated CAL-1 reporter cells uninduced and stimulated with R848 (5 µg/ml, for 0–1 h). PhosTag, phos-Tag-containing gel. **(G)** Immunoblots of indicated knockout CAL-1 cells. **(H)** IL-6 (left panel) or IFNβ (right panel) production of indicated CAL-1 cells stimulated for 24 h with R848 (5 μg/ml). **(I–K)** Immunoblots of indicated knockout CAL-1 cells stimulated with R848 (5 µg/ml, for 0–1 h) (I), CpG-B (ODN2006, 5 µM, for 0–3 h) (J) or TNFα (10 ng/ml, for 0–30 min) (K). In C, F, G, and I–K data are representative of two independent experiments. In E, data show mean ± SD of three independent experiments. In H, data show mean ± SD of three stimulation replicates from one experiment representative of three independent experiments. Source data are available for this figure: SourceData F1.

**Figure S1.**
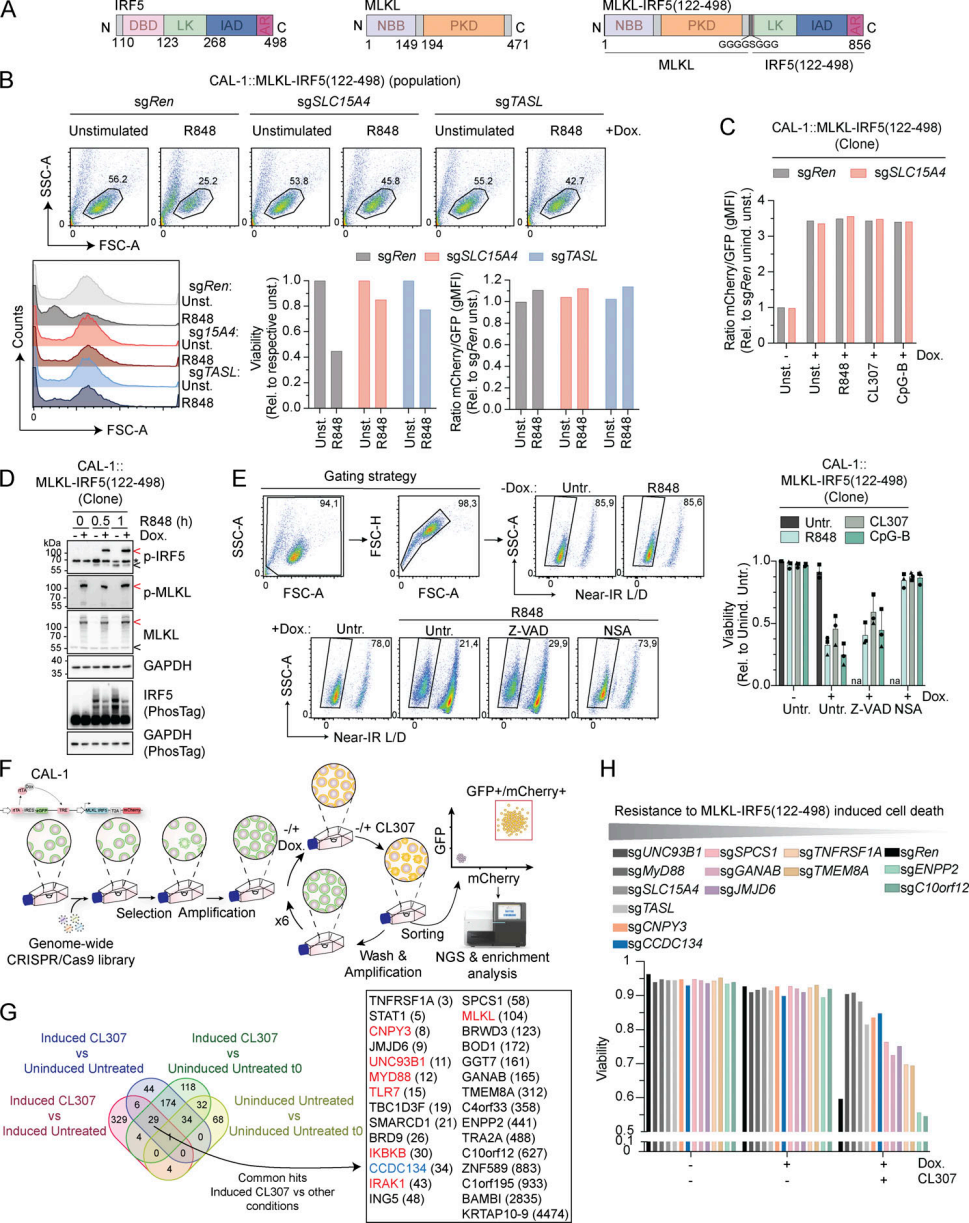
**Validation of MLKL-IRF5(122–498) reporter cell line and genome-wide CRISPR/Cas9 screening. (A)** Schematic of MLKL-IRF5(122–498) construct. DBD: DNA-binding domain; LK: linker region; IAD: IRF association domain; AR: auto-inhibitory region; NBB: N-terminal bundle and brace; PKD: pseudo kinase domain. **(B)** Representative dot-plot of FSC versus SSC gating used to assess cell viability (upper panel) with histogram for FSC (left lower panel), quantification of cell viability relative to the respective unstimulated (unst.) condition (middle lower panel) and ratio of mCherry/GFP gMFI relative to sg*Ren* unstimulated (unst.) cells (right lower panel). CAL-1 cells stably expressing MLKL-IRF5(122–498)-T2A-mCherry construct (population) and indicated knockout were induced with doxycycline (0.5 µg/ml) for 17 h before being stimulated or not with R848 (5 µg/ml) for 6 h. **(C)** Representative histogram of mCherry/GFP gMFI ratio (relative to sg*Ren* uninduced unstimulated [unind. unst.] condition) of CAL-1 reporter cells (clone) and indicated knockout. Cells were induced or not with doxycycline (Dox.) (0.5 µg/ml) for 17 h before being stimulated or not with R848 (2 µg/ml), CL307 (2 µg/ml) or CpG-B (ODN2006, 2 µM) for 6 h. **(D)** Immunoblots of CAL-1 reporter cells (clone) induced or not with doxycycline (Dox.) (0.5 µg/ml) for 17 h before being stimulated with R848 (5 µg/ml, for 0–1 h). Unlike the anti-phospho-IRF5 antibody, the IRF5 antibody used detects endogenous IRF5 but not the MLKL-IRF5(122–498)-T2A-mCherry construct. Red arrow indicates MLKL-IRF5(122–498) construct, black arrow endogenous IRF5 or MLKL and asterisk a non-specific band. **(E)** Representative dot-plot gating used to assess cell viability (left panel) and quantification of cell viability relative to uninduced untreated (unind. untr.) condition (right panel). CAL-1 cells stably expressing MLKL-IRF5(122–498)-T2A-mCherry construct (clone) were induced with doxycycline (Dox.) (0.5 µg/ml) and simultaneously treated or not with Z-VAD-FMK (Z-VAD) (20 µM) or NSA (5 µM) for 17 h before being stimulated or not with R848 (2 µg/ml) for 6 h. Small debris and cell aggregates were neglected using FSC and SSC gating while dead cells were excluded by gating on the negative/low population (Near-IR Live/Dead [L/D]). na: condition not assessed. **(F)** Schematic of genome-wide loss-of-function screen. **(G)** Venn diagram showing the overlap of hits with a fold change >1.55 for each of the indicated comparisons (left panel), and a list of the 29 overlapping hits in the different comparisons including the induced CL307 condition (right panel). The ranking, indicated in bracket, is based on the induced CL307 versus induced untreated comparison. **(H)** Cell viability quantification of an extended panel of CAL-1 reporter (clone) knockout cell lines of one independent experiment previously illustrated in Fig. 1 E. Cells were induced or not by doxycycline (Dox.) (0.5 µg/ml) for 17 h before being stimulated or not with CL307 (2 µg/ml) for 6 h. Data are representative of two (B–D) or one (H) independent experiments. In E (right panel), data show mean ± SD from three independent experiments. Source data are available for this figure: SourceData FS1.

Live/death read-outs are exquisitely powerful to perform genome-wide CRISPR/Cas9-based screens. To convert endolysosomal TLRs activation into a death-inducing event, we thought to take advantage of the fact that (1) TLRs engagement induces phosphorylation-dependent IRF5 dimerization and (2) optogenetic-induced dimerization of the necroptosis executioner mixed lineage kinase domain like pseudokinase (MLKL) is a potent trigger of cell death (Shkarina et al., 2022). We therefore designed a reporter system converting IRF5 activation into cell death by substituting its DNA binding domain with full-length MLKL (Fig. 1, A and B; and Fig. S1 A). The reporter, which allowed doxycycline-inducible expression of MLKL-IRF5(122–498)-T2A-mCherry (to monitor efficient expression), was stably expressed by lentiviral transduction in human CAL-1, a plasmacytoid dendritic (pDC) cell line competent for TLR7 and TLR9 signaling (Heinz et al., 2020; Maeda et al., 2005). Validating our approach, stimulation with endosomal TLR agonists R848 (TLR7/8), CL307 (TLR7), or CpG-B (TLR9) induced cell death in the reporter cell population as well as in a selected cell clone (Fig. 1 C; and Fig. S1, B and C). This was dependent on the upstream IRF5-activating complex formed by SLC15A4 and TASL (Fig. 1 C; and Fig. S1, B and C) (Heinz et al., 2020). Accordingly, upon R848 treatment, MLKL-IRF5 was rapidly phosphorylated in the IRF5 dimerization domain as detected by a specific phospho-S446-IRF5 antibody, while phospho-MLKL was already present upon doxycycline induction (Fig. S1 D). Confirming the induction of necroptotic cell death, this was blocked by the MLKL inhibitor necrosulfonamide (NSA) while pan-caspase inhibitor Z-VAD-FMK had no effect (Fig. S1 E). Next, we performed a genome-wide CRISPR/Cas9-based loss-of-function screen to identify essential components of the TLR7-induced IRF5 activation pathway (Fig. S1 F). Briefly, the MLKL-IRF5 reporter CAL-1 clone was transduced with a lentiviral whole-genome single-guide RNA (sgRNA) library and, after selection, cells were treated overnight with doxycycline to induce expression of the reporter and subsequently stimulated with CL307 for 6 h. After repeated treatments, mCherry-positive cells were sorted by FACS to select for CL307-resistant cells which retained the expression of the reporter. sgRNA abundance was then assessed by NGS in doxycycline-induced CL307-treated cells as well as in three control conditions: before the first treatment (time 0), as well as uninduced and doxycycline-induced unstimulated populations. Analysis for positively selected genes (i.e., conferring resistance to CL307-induced cell death when knockout) consistently identified across the different conditions highlighted several candidates (Fig. 1 D, Fig. S1 G, and Table S1). These comprise key pathway components (TLR7, MyD88, IRAK1, UNC93B1, CNPY3, IKKβ, and the reporter executioner MLKL) as well as putative new regulators. We first assessed the most promising novel candidates in the MLKL-IRF5 reporter system by establishing individual knockout lines (Fig. S1 H). Among these hits, we observed that deficiency of CCDC134, a previously poorly characterized protein proposed to be either secreted or acting in the nucleus (Huang et al., 2008, 2012), conferred the strongest protection from cell death, which was stable over time (Fig. 1 E and Fig. S1 H). Furthermore, CCDC134 knockout impaired endogenous IRF5 phosphorylation upon R848 treatment in uninduced reporter cells (i.e., in the absence of doxycycline-mediated MLKL-IRF5 expression), which was comparable to the loss of the TLR co-chaperone CNPY3 (Fig. 1 F). Altogether these results indicate that the screening approach was successful in identifying known and novel candidate components required for TLR7 responses, with CCDC134 representing a promising regulator.

To confirm the on-target effect of CCDC134 knockout and to further validate the impact on the endogenous IRF5 pathway, we assessed five independent sgRNA targeting CCDC134 in wildtype CAL-1 cells (Fig. 1 G). IL-6 and IFNβ production upon TLR7 stimulation was strongly reduced across these knockouts, similar to what was observed in CNPY3 and UNC93B1-deficient cells (Fig. 1 H). Accordingly, R848-induced IRF5 phosphorylation as well as NF-κB and MAPK pathways activation, monitored by IKKα/β, IκBα, and JNK phosphorylation, were impaired upon CCDC134 loss (Fig. 1 I). A similar profound defect in pathway activation was also observed after TLR9 stimulation (Fig. 1 J). Of note, CCDC134 deficiency did not interfere with NF-κB and MAPK activation induced by TNF, confirming the specificity for TLRs’ signaling and excluding a general impairment of cellular responses (Fig. 1 K). These data demonstrate the profound effect of CCDC134 deficiency on endolysosomal TLR7/9-induced responses and suggest an impact on early events proximal to receptor activation.

### CCDC134 is an ER-resident protein required for functional endolysosomal TLR7/9

**Figure 2.**
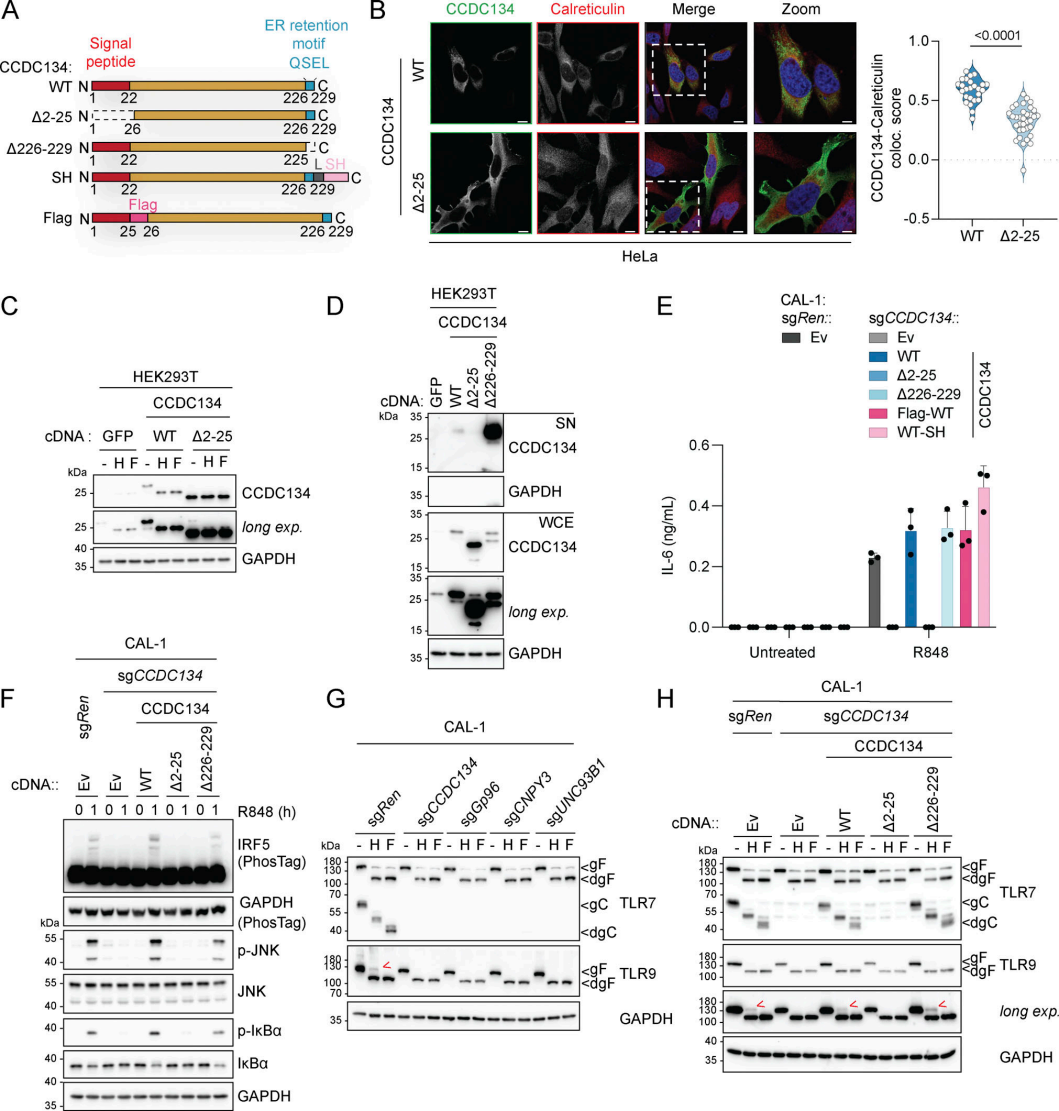
**Loss of the ER-resident protein CCDC134 impaired endolysosomal TLR7/9 maturation. (A)** Schematic of wildtype, deletion, or tagged CCDC134 constructs. L: linker, SH: Strep-HA tag. **(B)** Representative confocal microscopy images of HeLa cells transfected with wildtype or Δ2–25 mutant CCDC134 (left panel) and quantification of the colocalization between CCDC134 and calreticulin (right panel). Data are expressed as colocalization score (coloc. score) and pooled from three independent experiments. Each dot represents the analysis of a single field of view containing one to four transfected cells, and violins show the variation of individual dots across all experiments; P value <0.0001, two-tailed Mann–Whitney test. Green: anti-CCDC134; red: anti-Calreticulin; blue: DAPI. Scale bar: 10 μm. **(C)** Immunoblots of cell lysates treated with EndoH (H) or PNGase F (F) from HEK293T cells transfected as indicated. **(D)** Immunoblots of proteins precipitated from supernatant (SN) and whole-cell extracts (WCE) from HEK293T cells transfected as indicated. **(E)** IL-6 production of indicated CAL-1 cells stimulated for 24 h with R848 (5 μg/ml). **(F–H)** Immunoblots of lysates from indicated CAL-1 cells untreated (F) or treated with EndoH (H) or PNGase F (F) (G and H). Red arrows indicate the EndoH-resistant full-length form of TLR9. Ev: empty vector, gF: glycosylated full-length; dgF: deglycosylated full-length; gC: glycosylated cleaved form; dgC: deglycosylated cleaved form; long exp.: long exposure. In C, D, and F–H, data are representative of two independent experiments. In E, data show mean ± SD of three stimulation replicates from one experiment representative of three independent experiments. Source data are available for this figure: SourceData F2.

**Figure S2.**
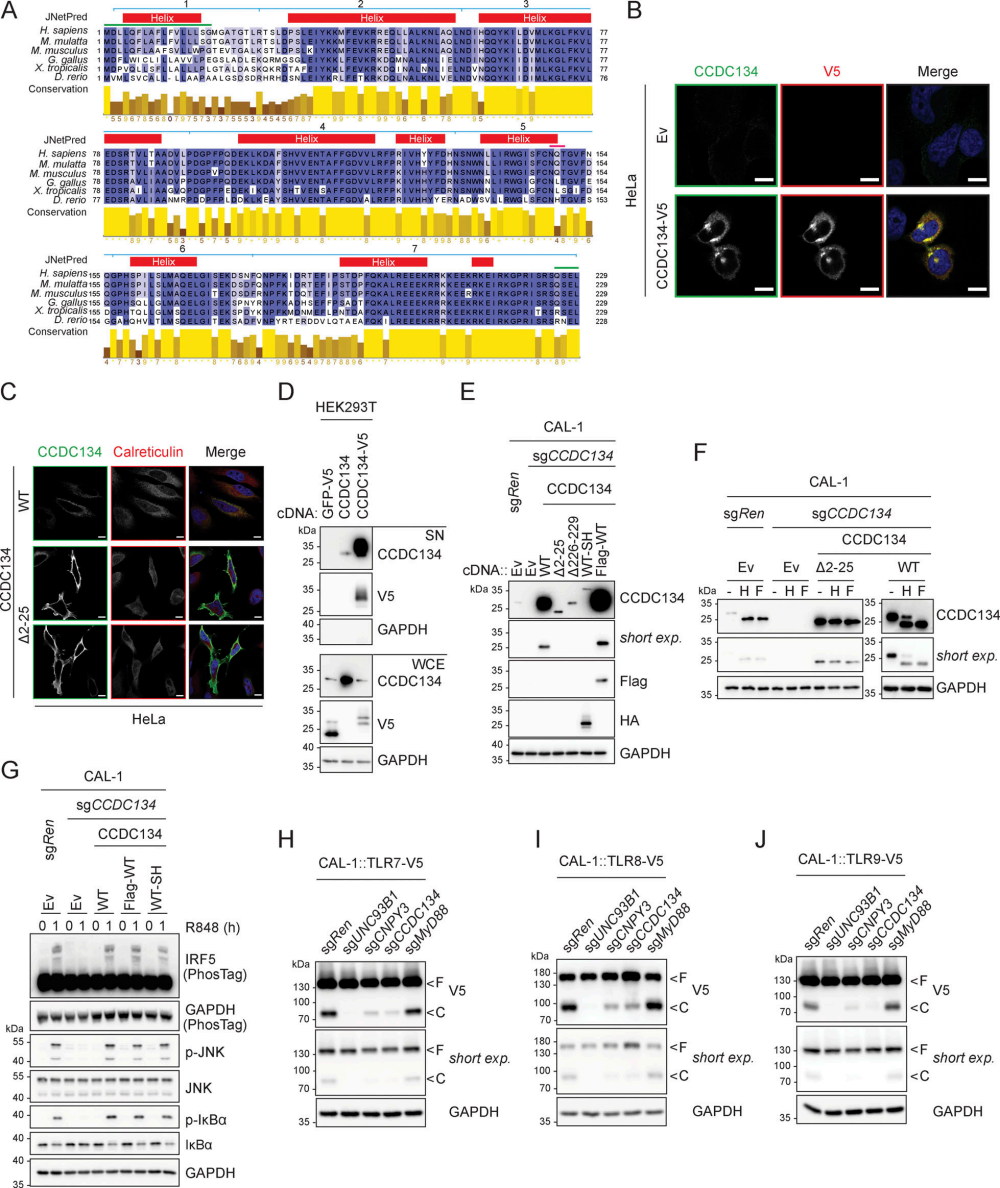
**CCDC134 is an ER-resident protein. (A)** Multiple sequence alignment of CCDC134 protein across species. UniProt entry names: CC134_HUMAN, G7N3Z8_MACMU, CC134_MOUSE, E1BVM7_CHICK, CC134_XENTR, and A0A8M6Z583_DANRE. Boxes above the alignment indicate consensus prediction from JPred4. Red: helix; blue lines: deleted regions of deletion mutant constructs used in this study (1: Δ2–25, 2: Δ25–57, 3: Δ57–91, 4: Δ91–133, 5: Δ133–156, 6: Δ156–178, 7: Δ178–229); pink line: predicted N-linked glycosylation on NQT sequon; green lines: signal peptide (1–22) or ER retention signal (226–229). **(B and C)** Representative confocal images microscopy of HeLa cells transfected as indicated. Green: anti-CCDC134; red: anti-Calreticulin; blue: DAPI. Scale bar: 10 μm. Ev: empty vector. **(D)** Immunoblots of proteins precipitated from supernatant (SN) and whole-cell extracts (WCE) of HEK293T cells transfected as indicated. **(E and F)** Immunoblots of lysate from indicated CAL-1 cells untreated (E) or treated with EndoH (H) or PNGase F (F) (F). Left and right panels of immunoblots in F are presented with two different exposures. Ev: empty vector; SH: strep-HA tag; short exp.: short exposure. **(G–J)** Immunoblots of indicated CAL-1 cells stimulated with R848 (5 µg/ml, for 0–1 h) (G) or untreated (H–J). F: full-length; C: cleaved form. Short exp.: short exposure. In D–J, data are representative of two independent experiments. Source data are available for this figure: SourceData FS2.

CCDC134 is a 229-amino acid protein, which has been proposed both to be secreted and have immune cytokine-like functions, inhibiting the MAPK pathway (Huang et al., 2008; Xia et al., 2018), as well as to localize in the nucleus and participate in DNA damage-induced responses (Huang et al., 2012). Furthermore, full-body CCDC134 knockout has been shown to be embryonically lethal while T cell–specific deletion attenuated TCR signaling (Yu et al., 2018; Zhang et al., 2023b). Conversely, transgenic mice overexpressing CCDC134 were protected in encephalomyelitis and arthritis models (Xia et al., 2017, 2018). Recently, CCDC134 loss-of-function mutations were identified in osteogenesis imperfecta patients (Ali et al., 2022; Dubail et al., 2020; Holick et al., 2021). Therefore, the mechanistic link between CCDC134 and TLRs remained unclear. CCDC134 is highly conserved across species, and sequence analysis identified a putative N-terminal signal peptide, in agreement with a previous report (Fig. 2 A and Fig. S2 A) (Huang et al., 2008; Teufel et al., 2022). Furthermore, we noticed a C-terminal QSEL sequence, which has been previously reported as an alternative ER retention motif (Raykhel et al., 2007), analogous to the classical KDEL, suggesting that CCDC134 is an ER-resident protein (Fig. 2 A and Fig. S2 A). To test this hypothesis and to define the subcellular localization of CCDC134, we generated deletion mutants lacking either the signal peptide (Δ2–25 CCDC134) or the potential ER retention QSEL motif (Δ226–229 CCDC134) (Fig. 2 A). In transfected HeLa cells, staining of wildtype CCDC134 with a CCDC134-specific antibody showed colocalization with the ER marker calreticulin (Fig. 2 B and Fig. S2, B and C). In contrast, Δ2–25 CCDC134 lost calreticulin colocalization and appeared diffuse in the cytoplasm (Fig. 2 B and Fig. S2 C). Furthermore, we observed that full-length CCDC134, but not the signal peptide Δ2–25 mutant, is N-glycosylated and sensitive to both endoglycosidase H (EndoH) and peptide-N-glycosidase F (PNGase F) treatments (Fig. 2 C). As modifications of glycans occurring during trafficking through the Golgi confer resistance to EndoH, this further indicates that CCDC134 is an ER-resident protein. In line with this, overexpressed wildtype or Δ2–25 CCDC134 proteins were largely absent in the supernatant of transfected HEK293T cells, while deletion of the putative ER retention QSEL motif or addition of a C-terminal tag, likely disrupting its function, led to CCDC134 secretion (Fig. 2 D and Fig. S2 D). Overall, these data indicate that CCDC134 is localized to the ER via a signal peptide and the ER retention motif QSEL.

To assess whether ER localization is required for the effect of CCDC134 on TLR responses, we stably reconstituted knockout CAL-1 cells with these different constructs. Moreover, we designed an internal Flag-tagged version of CCDC134 to prevent the secretion of the protein by interfering with the ER retention motif (Fig. 2 A). All proteins, and in particular wildtype and Flag-tagged CCDC134, were expressed at higher levels compared with endogenous CCDC134 (Fig. S2 E). Similar to what was observed in transfected HEK293T cells, CCDC134 was sensitive to both EndoH and PNGase F treatments while Δ2–25 CCDC134 was not glycosylated (Fig. S2 F). All the ER-targeted CCDC134 constructs fully restored TLR7 responses, both in terms of IRF5, NF-κB, and MAPK pathways activation, as well as downstream production of IL-6, while the signal peptide deficient CCDC134 Δ2–25 was inactive (Fig. 2, E and F; and Fig. S2 G). Of note, disruption of the ER retention motif by deletion or addition of a C-terminal tag did not affect CCDC134 functionality, indicating that transient localization to the ER is sufficient to rescue TLR7 signaling in these settings. Considering the requirement for ER localization and the proximal effect on TLR signaling, we hypothesized that CCDC134 might be involved in the folding, stability, and/or trafficking of endolysosomal TLRs, similar to UNC93B1 or ER-resident CNPY3 and Gp96. We therefore assessed whether CCDC134 deficiency affected the cleavage of the ectodomain of TLR7, TLR8, and TLR9, an essential process for their function (Majer et al., 2017; Miyake et al., 2021). In CAL-1 stably expressing C-terminal V5-tagged TLR7, TLR8, and TLR9, CCDC134 knockout resulted in a profound impairment of TLR processing, as shown by the substantial reduction in the cleaved, but not the full-length TLR forms, largely phenocopying CNPY3 or UNC93B1 deficiency (Fig. S2, H–J). This was confirmed by monitoring endogenous TLR7 cleavage. The mature cleaved form of TLR7, but not the full-length ER-localized form, was lost in CCDC134 deficient cells, similarly to knockouts of UNC93B1, Gp96 chaperone, and its co-chaperone CNPY3 (Fig. 2 G). In line with this, while the anti-TLR9 antibody used does not detect the cleaved form, we observed that the EndoH-resistant TLR9 form present in control cells, representing the minor fraction of full-length protein that trafficked through the Golgi, was absent in CCDC134 knockout cells, again similar to what was observed in Gp96-, CNPY3-, and UNC93B1-deficient cells (Fig. 2 G). TLR7 and TLR9 maturation was fully restored upon expression of ER-targeted CCDC134 constructs, but not the non-functional Δ2–25 mutant (Fig. 2 H).

These results demonstrate that in CCDC134-deficient cells, TLR7, 8, and 9 fail to undergo proteolytic cleavage in the endolysosomal compartment, possibly resulting from impairment of their folding and/or trafficking.

### CCDC134 interacts with the TLR chaperone Gp96 and is critical for its stability

**Figure S3.**
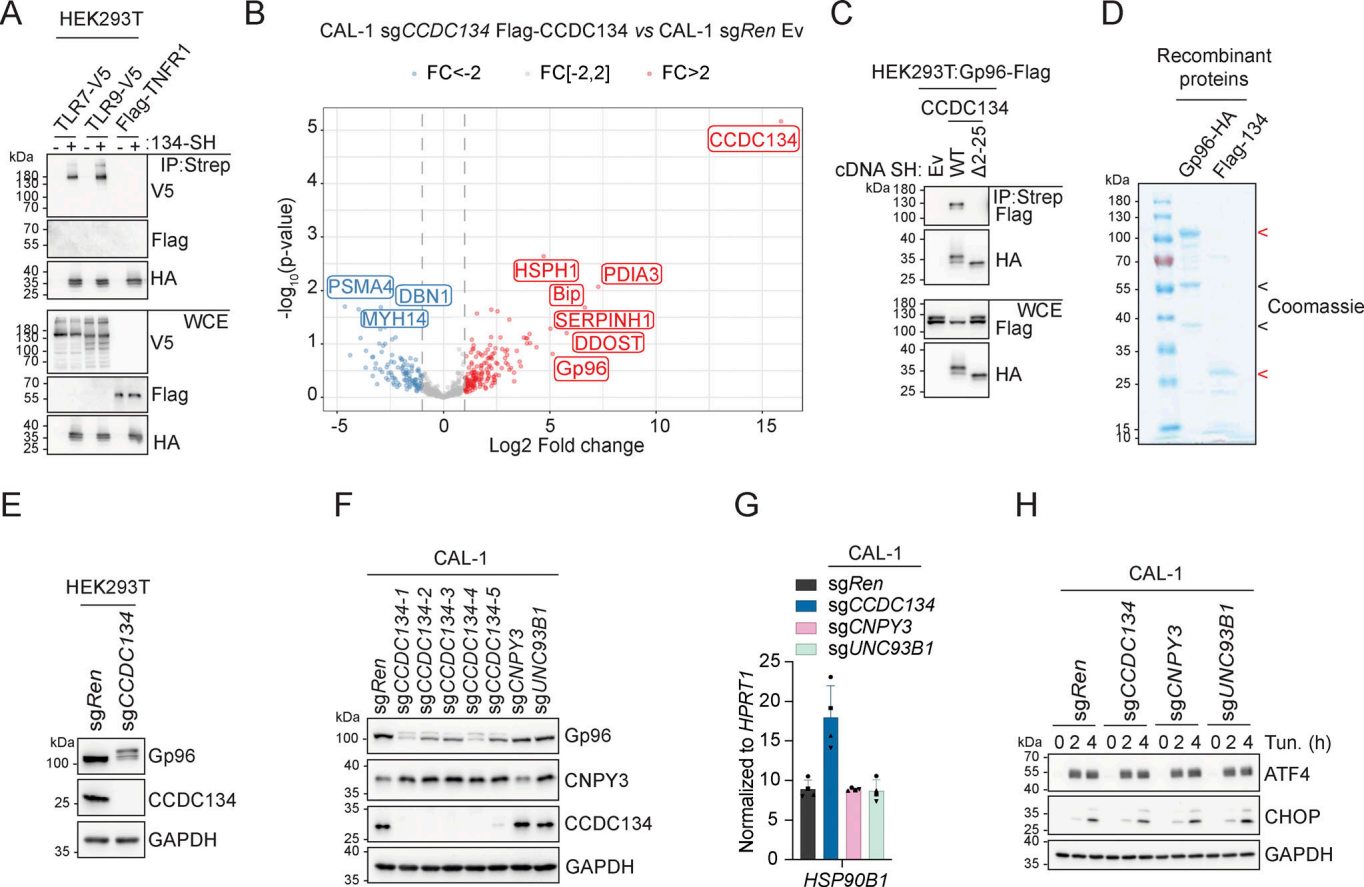
**CCDC134 controls Gp96 protein glycosylation and stability. (A)** Immunoprecipitates (IP) and whole-cell extracts (WCE) from HEK293T cells transfected as indicated. **(B)** Volcano plot of proteins, identified by mass spectrometry, from Flag-immunoprecipitates from sg*CCDC134* CAL-1 stably reconstituted with Flag-CCDC134 or control CAL-1 (sg*Ren* transduced with empty vector [EV]). red: fold change (FC) >2; gray: fold change (FC) = [−2, 2]; blue: fold change (FC) less than −2. **(C)** Immunoprecipitates (IP) and whole-cell extracts (WCE) from HEK293T cells transfected as indicated. **(D)** Coomassie blue staining of recombinant proteins Gp96-HA or Flag-CCDC134 (Flag-134) in a 10% sodium dodecyl sulphate–polyacrylamide gel electrophoresis (SDS–PAGE). Red arrow indicates specific Gp96-HA or Flag-CCDC134 bands; black arrow indicates putative C-terminal species of Gp96-HA. **(E)** Immunoblots of indicated knockout HEK293T cells. **(F)** Immunoblots of indicated CAL-1 cells. **(G)***HSP90B1* (gene coding for Gp96) mRNA levels of indicated CAL-1 cells measured by qPCR (normalized to *HPRT1*). **(H)** Immunoblots of indicated knockout CAL-1 cells treated with tunicamycin (Tun.) (5 µg/ml, for 0–4 h) or vehicle DMSO. In A, C–F, and H, data are representative of two independent experiments. In G, data show mean ± SD of four independent experiments. Source data are available for this figure: SourceData FS3.

**Figure 3.**
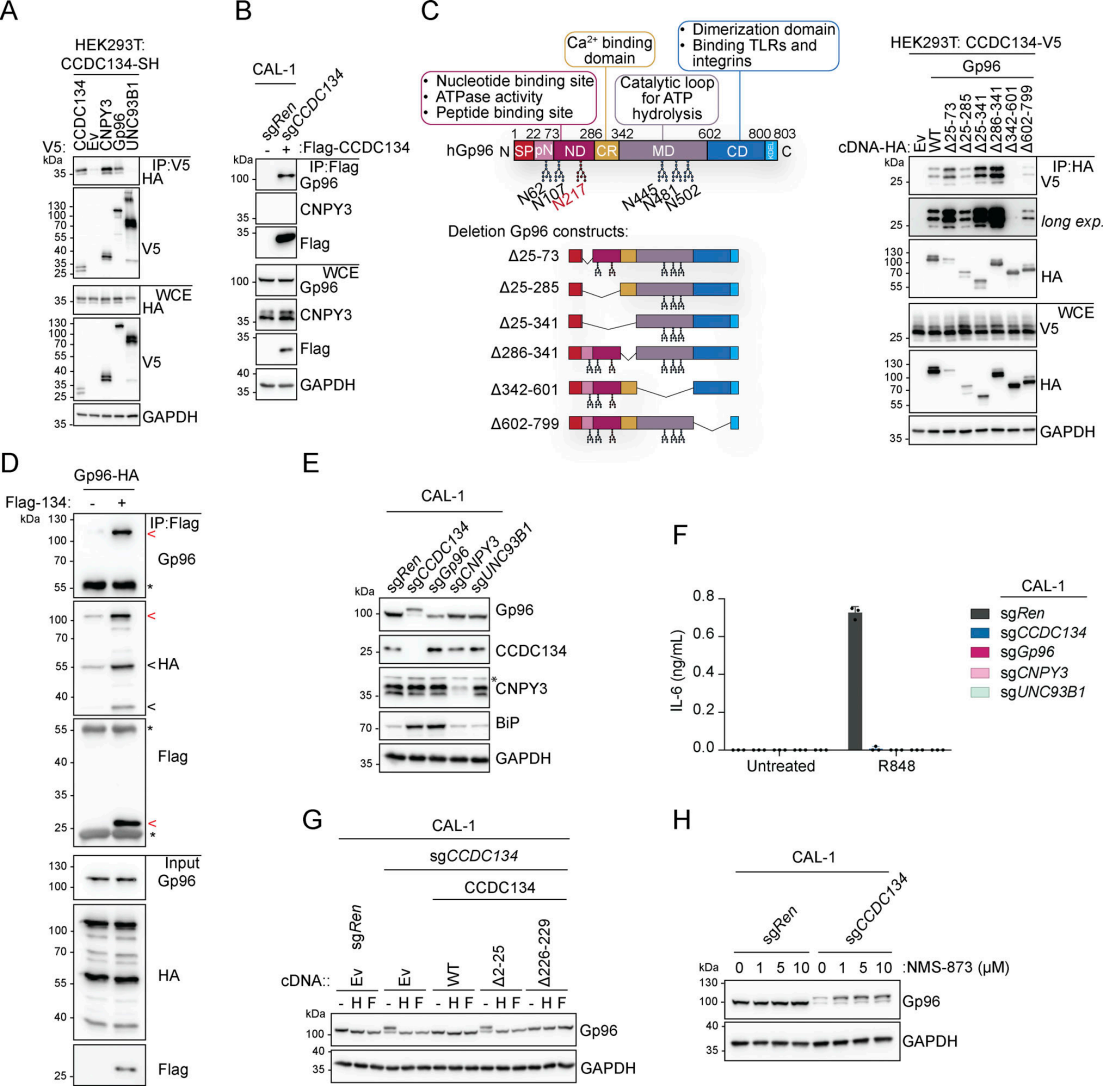
**CCDC134 interacts and stabilizes the ER chaperone Gp96. (A and B)** Immunoprecipitates (IP) and whole-cell extracts (WCE) from transfected HEK293T (A) or knockout CAL-1 cell lines (B) as indicated. **(C)** Schematic of wildtype Gp96 and deletion constructs (left panel) as well as immunoprecipitates (IP) and whole-cell extracts (WCE) from HEK293T transfected as indicated (right panel). SP: signal peptide, pN: pre-N-terminal domain; ND: N-terminal domain, CR: charged linker region; MD: middle domain; CD: C-terminal domain; KDEL: ER retention motif; steady state (N217 red) and cryptic N-glycans acceptor sites are shown. **(D)** Immunoprecipitates (IP) and input using Flag-CCDC134 and Gp96-HA recombinant proteins. For the complex formation, Flag-CCDC134 and Gp96-HA were preincubated overnight at 4°C before to perform the immunoprecipitation assay. Red arrow indicates specific Gp96-HA or Flag-CCDC134 bands; black arrow indicates putative C-terminal species of Gp96-HA; asterisks indicate IgG heavy or light chains. **(E)** Immunoblots of lysates from indicated knockout CAL-1 cells. Asterisks indicate a non-specific band. **(F)** IL-6 production of indicated knockout CAL-1 cells stimulated for 24 h with R848 (5 μg/ml). **(G)** Immunoblots of cell lysate treated with EndoH (H) or PNGase F (F) from indicated CAL-1 cell lines. **(H)** Immunoblots from indicated knockout CAL-1 cells treated with NMS-873 (1, 5, and 10 μM) or vehicle DMSO for 8 h. In A–E, G, and H, data are representative of two independent experiments. In F, data show mean ± SD of three stimulation replicates from one experiment representative of three independent experiments. Source data are available for this figure: SourceData F3.

**Figure 4.**
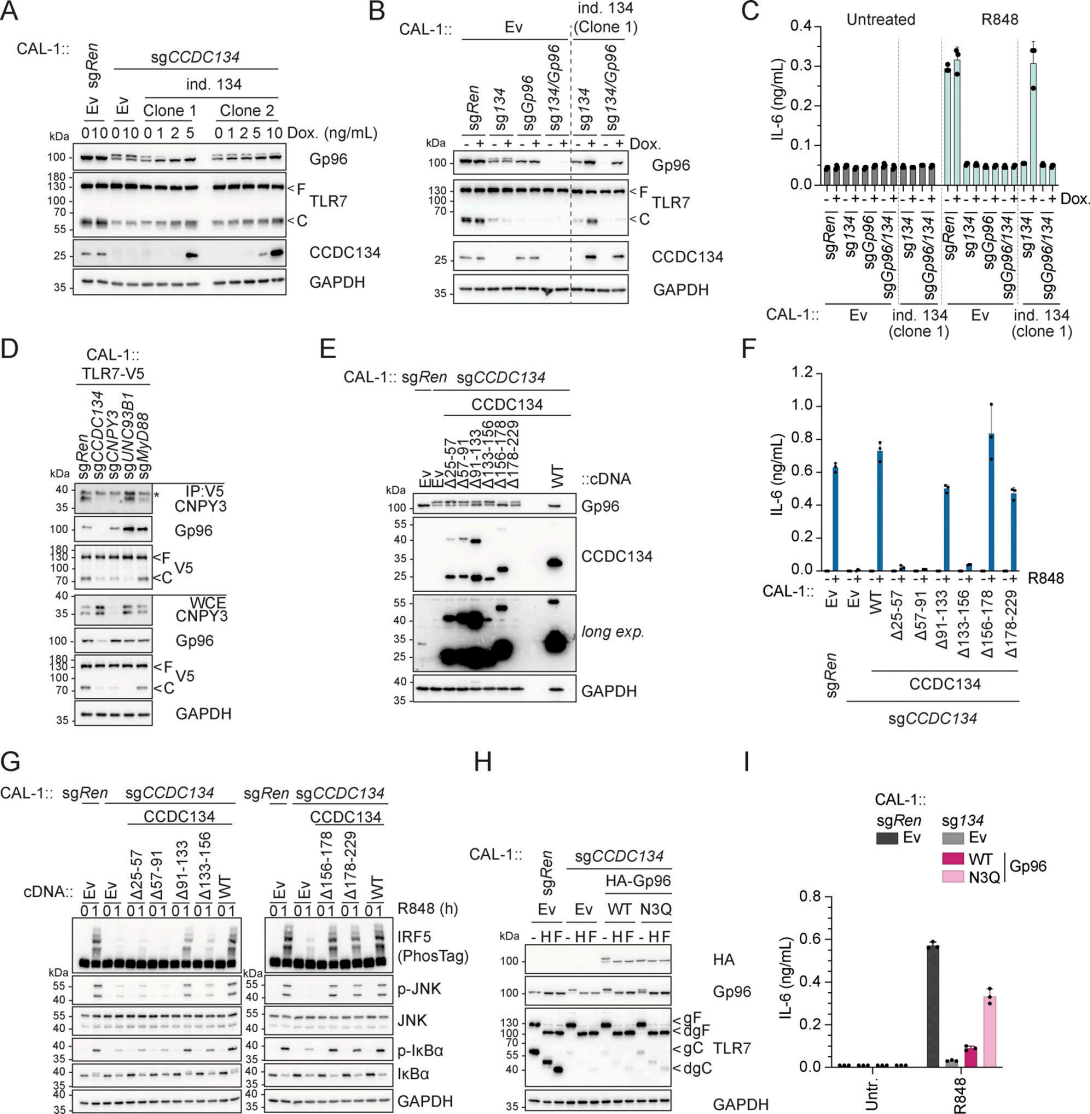
**CCDC134 modulates TLR7/9 signaling by regulating Gp96 hyperglycosylation. (A)** Immunoblots of knockout CAL-1 cells stably expressing a doxycycline-inducible CCDC134 (ind. 134) construct (clone 1 or 2) treated with the indicated concentration of doxycycline (Dox., 0–10 ng/ml) for 24 h. **(B)** Immunoblots of knockout CAL-1 cells stably expressing a doxycycline-inducible CCDC134 (ind. 134) construct (clone 1) treated with doxycycline (Dox., 5 ng/ml) for 24 h. **(C)** IL-6 production of indicated knockout CAL-1 cells expressing a doxycycline-inducible CCDC134 (ind. 134) construct (clone 1) induced with 5 ng/ml of doxycycline (Dox.) for 17 h and followed by 24 h stimulation with R848 (5 μg/ml). **(D)** Immunoprecipitates (IP) and whole-cell extracts (WCE) from TLR7-V5-expressing CAL-1 knockout cell lines as indicated. Asterisks indicate a non-specific band. **(E)** Immunoblots of lysates from indicated CAL-1 cell lines. long exp.: long exposure. **(F)** IL-6 production of indicated CAL-1 cells stimulated for 24 h with R848 (5 μg/ml). **(G)** Immunoblots of indicated CAL-1 cells treated with R848 (5 µg/ml, for 0–1 h). **(H)** Immunoblots of cell lysates treated with EndoH (H) or PNGase F (F). N3Q Gp96 bears mutations at positions N445Q-N481Q-N502Q. **(I)** IL-6 production of indicated CAL-1 cells stimulated for 24 h with R848 (5 μg/ml). In A, B, D, E, G, and H, data are representative of two independent experiments. In C, F, and I, data show mean ± SD of three stimulation replicates from one experiment representative of three independent experiments. Source data are available for this figure: SourceData F4.

**Figure S4.**
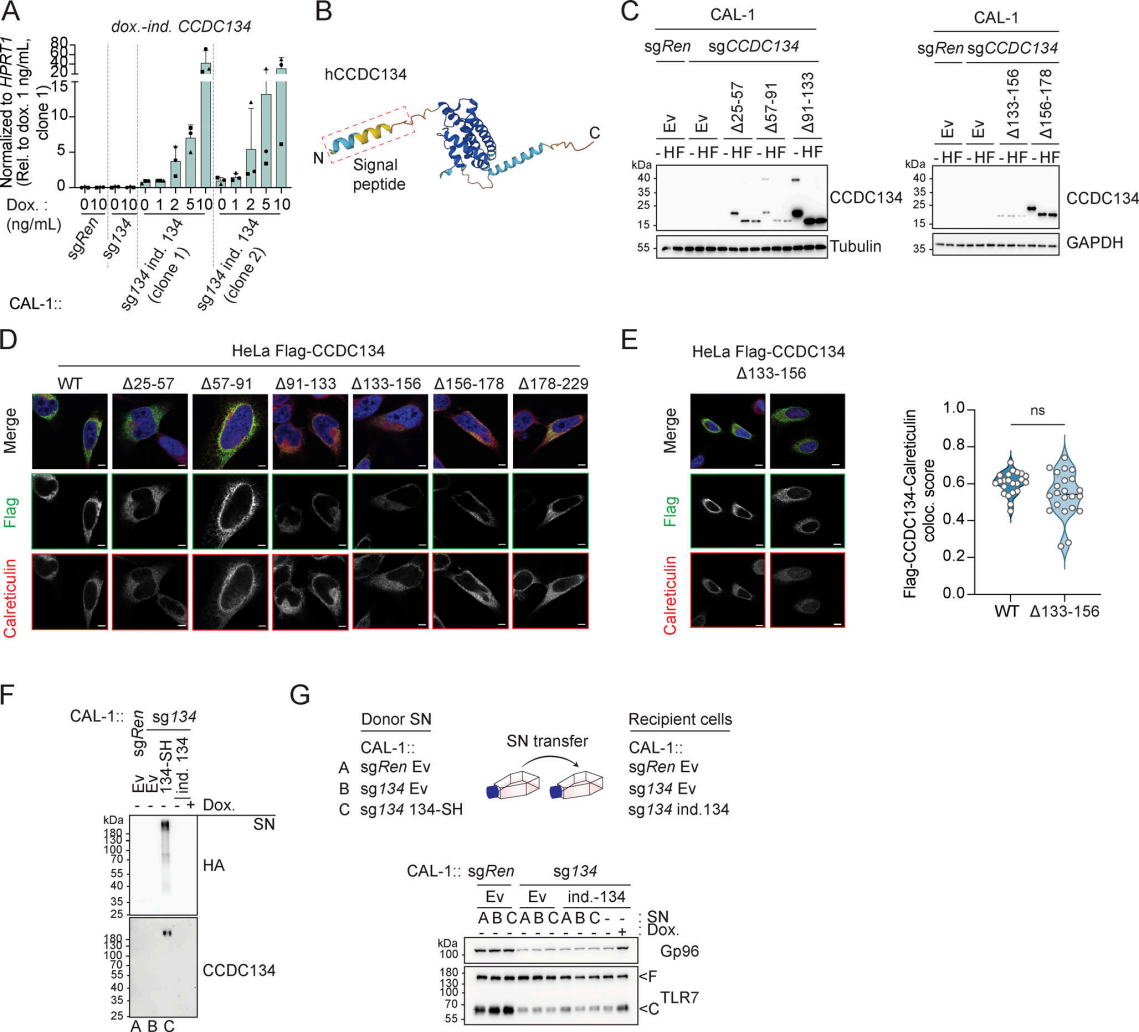
**Mapping of CCDC134 requirement for regulation of Gp96 in the ER. (A)** Doxycycline-inducible CCDC134 mRNA (*dox.-ind*. *CCDC134*) levels in indicated cells measured by qPCR. The primers were specifically designed to detect only the doxycycline-inducible construct, excluding any detection of the endogenous CCDC134 mrRNA. CAL-1 cells stably expressing the doxycycline-inducible CCDC134 construct (clone 1 or 2) were treated with the indicated concentration of doxycycline (Dox., 0–10 ng/ml) for 24 h. **(B)** AlphaFold structure prediction for human CCDC134 (Uniprot entry name: Q9H6E4 CC134_HUMAN). **(C)** Immunoblots of cell lysate treated with EndoH (H) or PNGase F (F) of indicated CAL-1 cells reconstituted with wildtype or deletion CCDC134 mutants. The Δ133–156 CCDC134 deletion mutant lacks the predicted N-linked glycosylated NQT sequon. Ev: empty vector. **(D)** Representative confocal microscopy images of HeLa cells transfected with Flag-tagged wildtype (WT) or deletion mutant CCDC134 constructs. Green: anti-Flag; red: anti-Calreticulin; blue: DAPI. Scale bar: 5 μm. **(E)** Representative confocal microscopy images of HeLa cells transfected with Flag-tagged Δ133–156 CCDC134 deletion mutant which lacks the predicted N-linked glycosylated NQT sequon (left panel) and quantification of the colocalization between Flag-CCDC134 (wildtype or Δ133–156 deletion mutant) and calreticulin (right panel). Data are expressed as colocalization score (coloc. score) and pooled from three independent experiments. Each dot represents analysis of a single field of view containing one to four transfected cells, and violins show the variation of individual dot across all experiments; P value = ns, two-tailed Mann–Whitney test. Green: anti-Flag; red: anti-Calreticulin; blue: DAPI. Scale bar: 5 μm. **(F)** Immunoblots of proteins from the (not precipitated) supernatant (SN) of indicated CAL-1 cells. Doxycycline-inducible CCDC134 cells (clone 1) were induced with doxycycline (5 ng/ml, Dox.) for 24 h. SH: strep-HA tag, ind. 134: doxycycline-inducible CCDC134. **(G)** Immunoblots of indicated CAL-1 cells incubated for 48 h with supernatants (SN) of donor cells cultured for 24 h in Opti-MEM. Doxycycline-inducible CCDC134 cells (clone 1) were induced with doxycycline (5 ng/ml, Dox.) for 48 h. SH: strep-HA tag, ind. 134: doxycycline-inducible CCDC134. In A, data show mean ± SD of three independent experiments. In C, F, and G, data are representative of two independent experiments. Source data are available for this figure: SourceData FS4.

Based on these data and the fact that CCDC134 deficiency mirrored the loss of the critical TLR7/9 folding and trafficking regulators UNC93B1 and CNPY3, we next assessed whether CCDC134 could interact with these factors. When co-expressed in HEK293T cells, CCDC134 co-immunoprecipitated TLR7 and TLR9, but not TNFR1 (Fig. S3 A). Furthermore, in these settings, we detected interaction between CCDC134 and CNPY3 as well as Gp96, while no binding to UNC93B1 was observed (Fig. 3 A). CCDC134 was also co-immunoprecipitated, indicating possible oligomerization. To unbiasedly investigate the interactome of CCDC134 in more physiological settings, we performed mass spectrometry analysis upon immunoprecipitation of Flag-CCDC134 from stably reconstituted CCDC134 knockout CAL-1 cells and identified Gp96 as one of the strong binders (Fig. S3 B and Table S2). Indeed, Flag-CCDC134 co-immunoprecipitated endogenous Gp96, while its co-chaperone CNPY3 was not detected in these conditions, suggesting that Gp96 is the key interactor (Fig. 3 B). As predicted, binding occurred in the ER as only wildtype CCDC134 and not the signal peptide–deleted mutant ∆2–25 associated with Gp96 (Fig. S3 C). Moreover, the interaction required the middle and C-terminal domains of Gp96 (Fig. 3 C). Strongly supporting direct interaction between CCDC134 and Gp96, this was observed also in vitro using purified recombinant proteins (Fig. 3 D and Fig. S3 D). Strikingly, we observed that the loss of CCDC134 had a profound impact on Gp96 protein, supporting the functional relevance of this interaction (Fig. 3 E). CCDC134 deficiency in CAL-1 cells resulted in reduced total protein levels and the appearance of a higher molecular weight form of Gp96, while the co-chaperone CNPY3 was not affected (Fig. 3 E). This effect was specific for CCDC134, as Gp96 levels were normal in CNPY3- and UNC93B1-deficient cells, and was also observed in HEK293T (Fig. 3 E and Fig. S3 E). Across the different CCDC134 sgRNA CAL-1 lines, the amount of the high-molecular weight form of Gp96 and the decrease of its total protein level correlated with the reduction in R848-induced IL-6 production previously observed (Fig. 1 H and Fig. S3 F). Gp96 knockout similarly impaired R848-induced responses as expected, but did not alter CCDC134 or CNPY3 protein levels (Fig. 3, E and F). CCDC134 affected Gp96 at the protein level, as CCDC134 deficiency did not reduce Gp96 mRNA, which was in contrast moderately increased, possibly reflecting compensatory mechanisms (Fig. S3 G). Loss of Gp96 has been reported to induce upregulation of ER chaperone BiP (Dersh et al., 2014; Eletto et al., 2012), which was also observed in CCDC134- but not CNPY3-deficient cells (Fig. 3 E). Similar to Gp96 deficiency (Eletto et al., 2012), CCDC134 knockout did not have a major impact on ER-stress responses at steady state or upon tunicamycin treatment, as monitored by ATF4 and CHOP levels (Fig. S3 H). While Gp96 is mostly monoglycosylated at steady state, it has been reported to undergo aberrant hyperglycosylation under condition of cell stress (Cherepanova et al., 2019; Dersh et al., 2014; Yan et al., 2020). Hyperglycosylation occurs at cryptic N-glycan acceptor sites which are usually not modified, leading to a Gp96 form with reduced protein stability (Cherepanova et al., 2019; Dersh et al., 2014) (Fig. 3 C). The higher molecular weight band observed in CCDC134 knockout cells was sensitive to both EndoH and PNGase F treatments, consistent with representing hyperglycosylated Gp96 (Fig. 3 G). Gp96 glycosylation pattern and protein levels were restored in knockout cells stably reconstituted with ER-targeted CCDC134 constructs (Fig. 3 G). Treatment of CCDC134 knockout cells with NMS-873, an inhibitor of the essential component of the ERAD pathway p97/VCP, resulted in the accumulation of hyperglycosylated Gp96, suggesting the involvement of this pathway in its degradation (Fig. 3 H). As stable reconstitution of CCDC134 in CAL-1 knockout cells resulted in supra-endogenous expression levels, we further confirmed these findings by titrating CCDC134 expression using a doxycycline-inducible system. In two independent clonal populations, induction of CCDC134 abolished Gp96 hyperglycosylation and restored both its protein levels as well as its functionality as indicated by the effect on TLR7 cleavage (Fig. 4 A and Fig. S4 A). Of note, CCDC134 had a significant impact on Gp96 even when expressed below the endogenous level (Fig. 4 A). Moreover, deletion of Gp96 impaired the rescuing effect of CCDC134 induction both on TLR7 cleavage and cytokine responses, further indicating that CCDC134 acts through Gp96 (Fig. 4, B and C). Supporting the key role of CCDC134 for Gp96- and CNPY3-mediated folding of TLRs, CCDC134 deficiency impaired recruitment of both endogenous Gp96 and CNPY3 to TLR7, while CNPY3 loss did not affect Gp96 binding (Fig. 4 D). Next, we mapped the regions of CCDC134 required for its function. CCDC134 is predicted to form a globular protein composed of six alpha helices, which we individually deleted (Fig. S2 A and Fig. S4 B). Deletion did not affect the localization as all constructs were targeted to the ER, including the Δ133–156 mutant lacking the CCDC134 glycosylation site (Fig. S2 A and Fig. S4, C–E). Upon stable reconstitution in CCDC134-deficient cells, we observed that constructs that reduced Gp96 hyperglycosylation (Δ91–133, Δ156–178, and Δ178–229, which lacks the ER retention sequence) restored also R848-induced TLR7/8 signaling (Fig. 4, E–G). To further confirm that CCDC134 exerted its function by regulating Gp96 glycosylation in the ER and not through its previously proposed secreted form, which we observe only upon disruption of the newly identified ER retention sequence, we assessed whether transfer of cellular supernatant from CCDC134 expressing cells could rescue CCDC134 knockout. CCDC134 was detectable only as a high molecular weight form in the supernatant of cells expressing C-terminally tagged CCDC134 but not from cells expressing endogenous or doxycycline-inducible untagged CCDC134, which preserves a functional ER retention motif (Fig. S4 F). In line with CCDC134 exerting its function in the ER, none of these supernatants could rescue Gp96 level nor TLR7 cleavage in CCDC134 knockout cells (Fig. S4 G). Lastly, we reasoned that if the effect of CCDC134 loss is fully mediated by the induction of Gp96 hyperglycosylation and degradation, preventing this glycosylation should rescue TLR responses in CCDC134 knockout cells. Therefore, we reconstituted these cells either with Gp96 wildtype or mutagenized for three glycosylation sites present in its middle domain (N445Q-N481Q-N502Q, referred as Gp96 N3Q), as we observed that the construct lacking this region (∆342–601) did not show hyperglycosylation when overexpressed (Fig. 3 C). Supporting our model, while Gp96 wildtype showed hyperglycosylation, this was not observed for Gp96 N3Q protein, which accordingly accumulated at higher levels (Fig. 4 H). Importantly, Gp96 N3Q could partially restore TLR7 processing and rescued R848-induced IL-6 production in CCDC134 knockout cells, while expression of wildtype Gp96 was largely ineffective (Fig. 4, H and I).

Altogether, these data show that CCDC134 interacts with Gp96 in the ER and that loss of CCDC134 results in Gp96 hyperglycosylation and ERAD-dependent degradation, which in turn impairs endosomal TLRs’ processing and function. Moreover, these findings suggest that CCDC134 has an upstream and more central role in Gp96 function than the co-chaperone CNPY3, which does not impact Gp96 protein stability.

### CCDC134 is essential for Gp96-regulated TLRs

**Figure 5.**
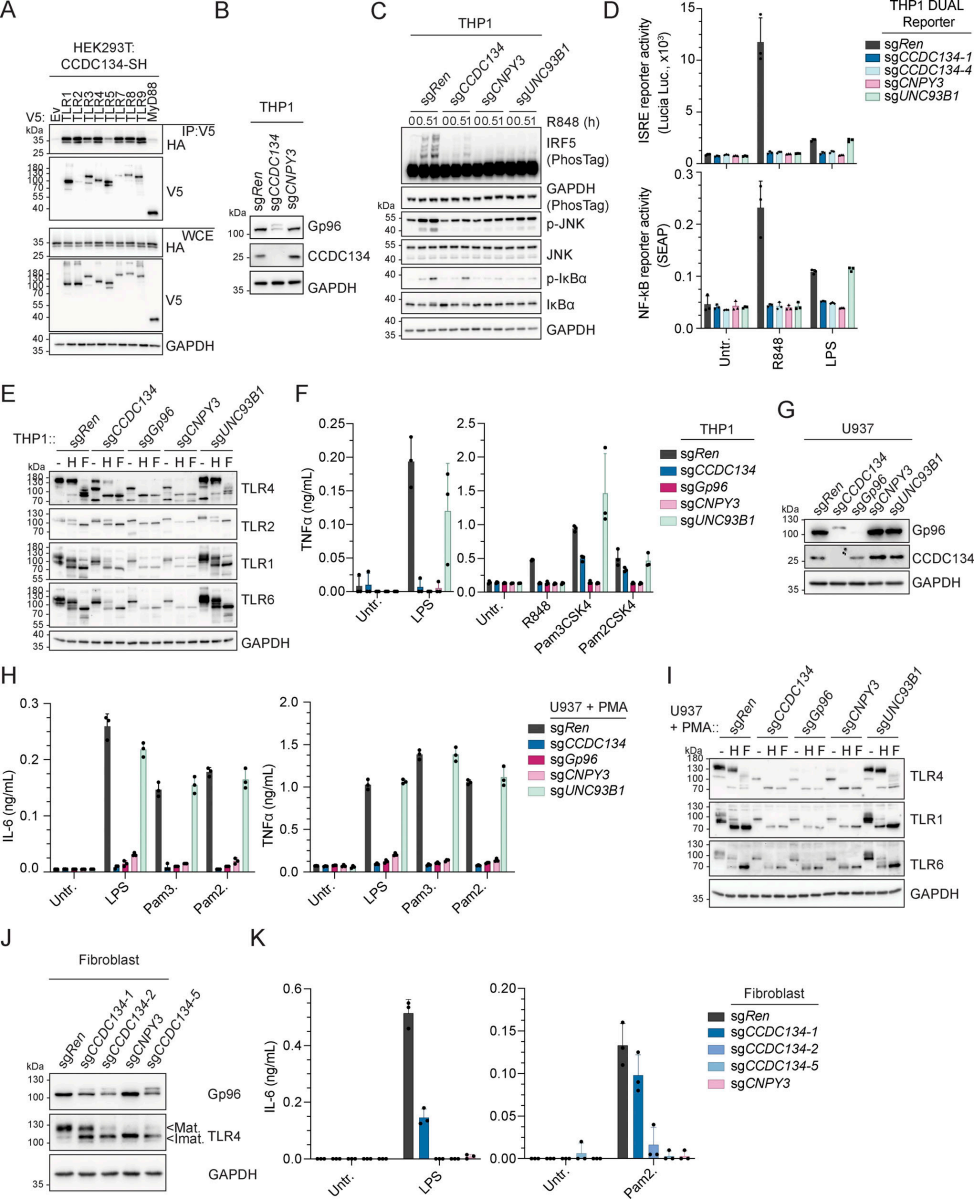
**CCDC134 deficiency selectively impaired TLR-mediated immune responses. (A)** Immunoprecipitates (IP) and whole-cell extracts (WCE) from HEK293T cells transfected as indicated. SH: Strep-HA tag. **(B and C)** Immunoblots of indicated knockout THP1 cells unstimulated (B) or stimulated with R848 (5 µg/ml, for 0–1 h) (C). **(D)** Indicated knockout THP1 DUAL reporter cells stimulated with R848 (5 μg/ml) or LPS (0.1 μg/ml) for 24 h. Supernatants were analyzed for ISRE and NF-κB reporter activity. Untr.: untreated. **(E)** Immunoblots of cell lysates from indicated knockout THP1 cells treated with EndoH (H) or PNGase F (F). **(F)** TNFα production of indicated knockout THP1 cells stimulated for 24 h with LPS (0.1 μg/ml), R848 (5 μg/ml), Pam3CSK4 (0.1 μg/ml) or Pam2CSK4 (0.01 μg/ml). Untr.: untreated. **(G)** Immunoblots of cell lysates from indicated knockout U937 cells. **(H)** IL-6 (left panel) and TNFα (right panel) production of indicated knockout U937 cells differentiated with 200 nM of PMA for 24 h before stimulation with LPS (0.1 μg/ml), Pam3CSK4 (Pam3.) (1 μg/ml), or Pam2CSK4 (Pam2.) (0.1 μg/ml) for 24 h. Untr.: untreated. **(I and J)** Immunoblots of cell lysates from indicated knockout U937 cells differentiated with 200 nM of PMA for 48 h, treated with EndoH (H) or PNGase F (F) (I) or from indicated knockout human primary dermal fibroblast cells (J). Mat.: Mature form; Imat.: Immature form. **(K)** IL-6 production of indicated knockout human primary dermal fibroblast cells stimulated with LPS (0.1 μg/ml) or Pam2CSK4 (Pam2.) (0.01 μg/ml) for 24 h. Untr.: untreated. In A–C, E, G, I, and J, data are representative of two independent experiments. In D, F, H, and K, data show mean ± SD of three stimulation replicates from one experiment representative of three independent experiments. Source data are available for this figure: SourceData F5.

**Figure S5.**
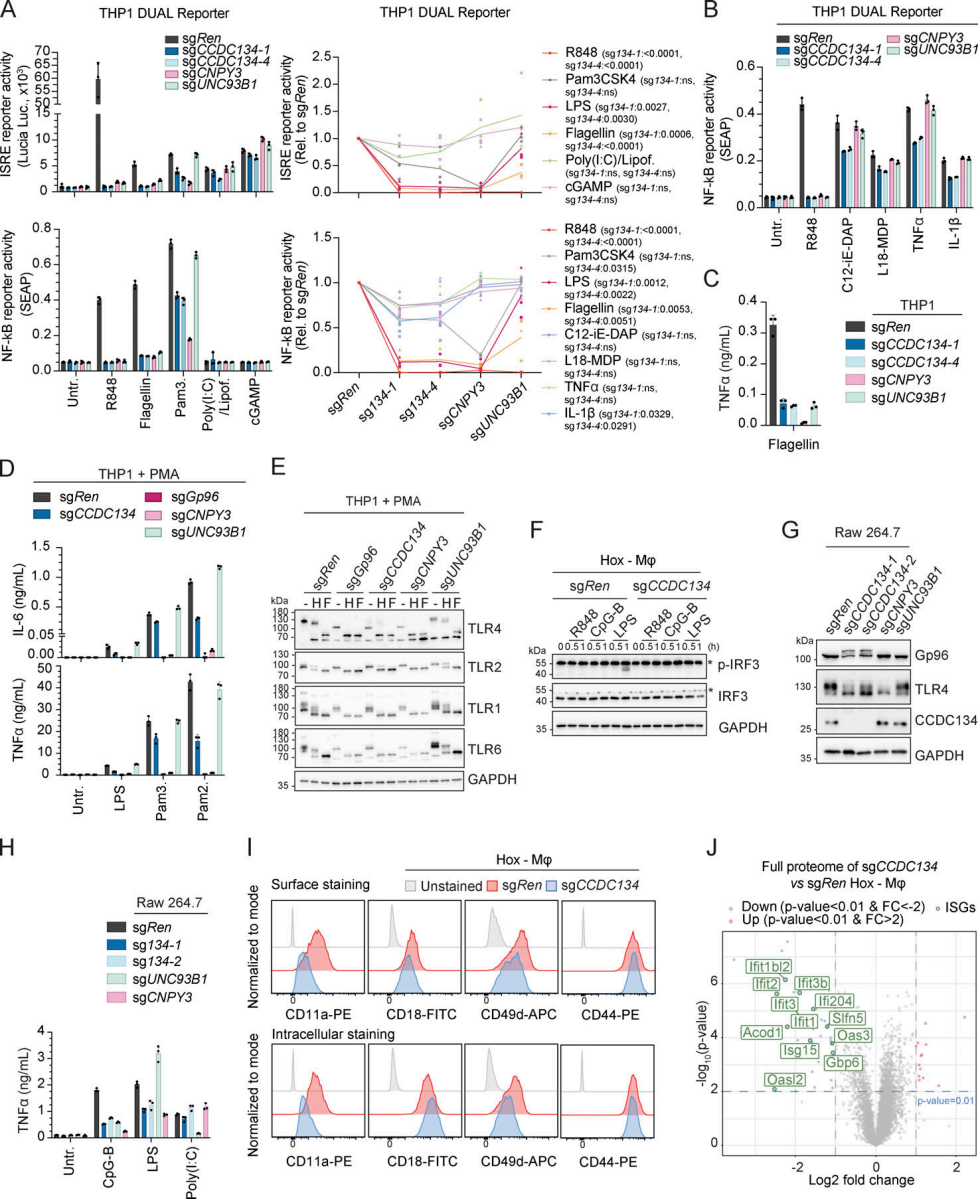
**Impact of CCDC134 loss on TLRs and integrins. (A and B)** THP1 DUAL reporter cells stimulated with R848 (5 μg/ml), flagellin (0.1 μg/ml), Pam3CSK4 (Pam3.) (0.1 μg/ml), LPS (0.1 μg/ml), Poly(I:C) complexed with lipofectamine (1 μg/ml), cGAMP (3 μg/ml), C12-iE-DAP (5 μg/ml), L18-MDP (10 μg/ml), TNFα (10 ng/ml), or IL-1β (10 ng/ml) for 24 h. Supernatants were analyzed for ISRE and NF-κB reporter activity. **(A, left panel, and B)** Data show mean ± SD from one representative experiment performed in stimulation triplicates. **(A, right panel)** Reporter activity relative to sg*Ren*. Data show three independent experiments. Line represents the mean ± SD ISRE reporter activity, R848 sg*CCDC134-1* P value <0.0001, sg*CCDC134-4* P value <0.0001; Pam3CSK4 sg*CCDC134-1* P value = ns, sg*CCDC134-4* P value = ns; LPS sg*CCDC134-1* P value <0.0027, sg*CCDC134-4* P value <0.0030; flagellin sg*CCDC134-1* P value = 0.0006, sg*CCDC134-4* P value <0.0001; Poly(I:C) complexed with lipofectamine sg*CCDC134-1* P value = ns, sg*CCDC134-4* P value = ns; cGAMP sg*CCDC134-1* P value = ns, sg*CCDC134-4* P value = ns. NF-κB reporter activity, R848 sg*CCDC134-1* P value <0.0001, sg*CCDC134-4* P value <0.0001; Pam3CSK4 sg*CCDC134-1* P value = ns, sg*CCDC134-4* P value = 0.0315; LPS sg*CCDC134-1* P value = 0.0012, sg*CCDC134-4* P value = 0.0022; flagellin sg*CCDC134-1* P value = 0.0053, sg*CCDC134-4* P value = 0.0051; C12-iE-DAP sg*CCDC134-1* P value = ns, sg*CCDC134-4* P value = ns; L18-MDP sg*CCDC134-1* P value = ns, sg*CCDC134-4* P value = ns; TNFα sg*CCDC134-1* P value = ns, sg*CCDC134-4* P value = ns; IL-1β sg*CCDC134-1* P value = 0.0329, sg*CCDC134-4* P value = 0.0291. Two-tailed one sample *t* test. Untr.: untreated. **(C)** TNFα production of indicated knockout THP1 cells stimulated with flagellin (0.1 μg/ml) for 24 h. **(D)** IL-6 (Top panel) and TNFα (Bottom panel) production of indicated knockout THP1 cells differentiated with 10 nM of PMA for 24 h before stimulation with LPS (0.1 μg/ml), Pam3CSK4 (Pam3.) (0.1 μg/ml) or Pam2CSK4 (Pam2.) (0.1 μg/ml) for 24 h. Untr.: untreated. **(E)** Immunoblots of cell lysate treated with EndoH (H) or PNGase F (F) of indicated knockout THP1 cells differentiated with 10 nM of PMA for 48 h. **(F and G)** Immunoblots of indicated knockout Hoxb8-macrophages stimulated with R848 (0.1 μg/ml), CpG-B (ODN1668) (1 μM) or LPS (10 ng/ml) for 0–1 h (F) or knockout Raw 264.7 cells (G). Asterisks indicate a non-specific band. **(H)** TNFα production of indicated knockout Raw 264.7 cells stimulated with CpG-B (ODN1668) (150 nM), LPS (10 ng/ml), or poly(I:C) (500 ng/ml) for 24 h. **(I)** Representative histograms of surface (upper panel) or surface and intracellular (lower panel) expression of CD11a, CD18, CD49d, or CD44 in sg*Ren* (red) or sg*CCDC134* (blue) Hoxb8-macrophages. Gray curves represent unstained controls. **(J)** Volcano plot of quantified proteins in whole proteome of sg*CCDC134* versus sg*Ren* Hoxb8-macrophages (upregulated: red, fold change [FC] >2 and P value <0.01; downregulated: blue, FC less than −2 and P value <0.01); green: downregulated ISGs signature. In A–D and H, data show mean ± SD of three stimulation replicates from one experiment representative of three independent experiments. In E–G, data are representative of two independent experiments. In I, data are representative of three independent experiments. Source data are available for this figure: SourceData FS5.

The chaperone activity of Gp96 and its TLR-specific co-chaperone CNPY3 is not restricted to the folding and trafficking of endolysosomal TLR7/9 but also impacts plasma membrane–localized TLRs (Liu et al., 2010; Randow and Seed, 2001; Takahashi et al., 2007; Wakabayashi et al., 2006; Yang et al., 2007). When co-expressed in HEK293T cells, CCDC134 could be co-immunoprecipitated with all TLRs tested (Fig. 5 A). To investigate TLR responses more broadly and to confirm that the requirement of CCDC134 was not restricted to the pDC cell line CAL-1, we next assessed monocytic THP1 cells. CCDC134 deletion strongly induced Gp96 hyperglycosylation and degradation in these cells as well (Fig. 5 B). Accordingly, CCDC134 deficiency impaired R848-induced responses in both wildtype and THP1 Dual cells, which express NF-κB– and ISRE-dependent reporters (Fig. 5, C and D). In these reporter cells, TLR4 and TLR5 responses upon LPS or flagellin stimulations were strongly impaired, while TLR1/2 (Pam3CSK4) were only partially affected (Fig. 5 D and Fig. S5 A). In contrast, CCDC134 deficiency had no or minor effect on cytoplasmic sensing by RIG-I/MDA5 (by transfected poly(I:C)), STING (cGAMP), NOD1 (C12-ie-DAP), or NOD2 (L18-MDP) receptors, and on plasma membrane TNF- and IL-1-receptors (Fig. S5, A and B). Next, we assessed whether CCDC134 deficiency affected plasma membrane TLRs’ trafficking and maturation similarly as observed on their endosomal TLRs’ counterpart. Indeed, CCDC134 knockout strongly reduced TLR4 maturation as monitored by the decrease of the EndoH-resistant form (Fig. 5 E). While this defect was less pronounced compared with Gp96 and CNPY3 knockouts, it resulted in strong impairment of LPS-induced TNF production (Fig. 5 F). TLR5-mediated responses were also diminished (Fig. S5 C). Similar maturation defect was observed on TLR2 co-receptors, TLR1 and TLR6, while TLR2 itself was less affected, consistent with limited effect on Pam3CSK4- (TLR1/2) and Pam2CSK4- (TLR6/2) induced responses (Fig. 5, E and F). Comparable results were obtained in PMA-differentiated THP1 macrophage cells (Fig. S5, D and E). The strong but partial impact observed in CCDC134 deficient cells compared with Gp96 and CNPY3 knockouts could possibly result from residual Gp96 activity in the CCDC134-targeted cell population or from cell type–specific effects. Therefore, we assessed human U937 cells that respond to these TLR agonists upon PMA-induced differentiation to macrophages. In line with CCDC134 being central for Gp96-mediated TLR biogenesis, CCDC134 knockout fully phenocopied Gp96 and CNPY3 loss, affecting TLR4, 1, and 6 maturations as well as responses to LPS, Pam3CSK4, and Pam2CSK4 (Fig. 5, G–I). The crucial role of CCDC134 was not restricted to immortalized myeloid cell lines as this could be recapitulated in human primary dermal fibroblasts. Indeed, CCDC134 targeting with three independent sg*CCDC134* resulted in Gp96 hyperglycosylation, impaired TLR4 maturation, and reduced LPS- and Pam2CSK4-induced IL-6 production (which correlates to the impact on Gp96 protein level) (Fig. 5, J and K).

**Figure 6.**
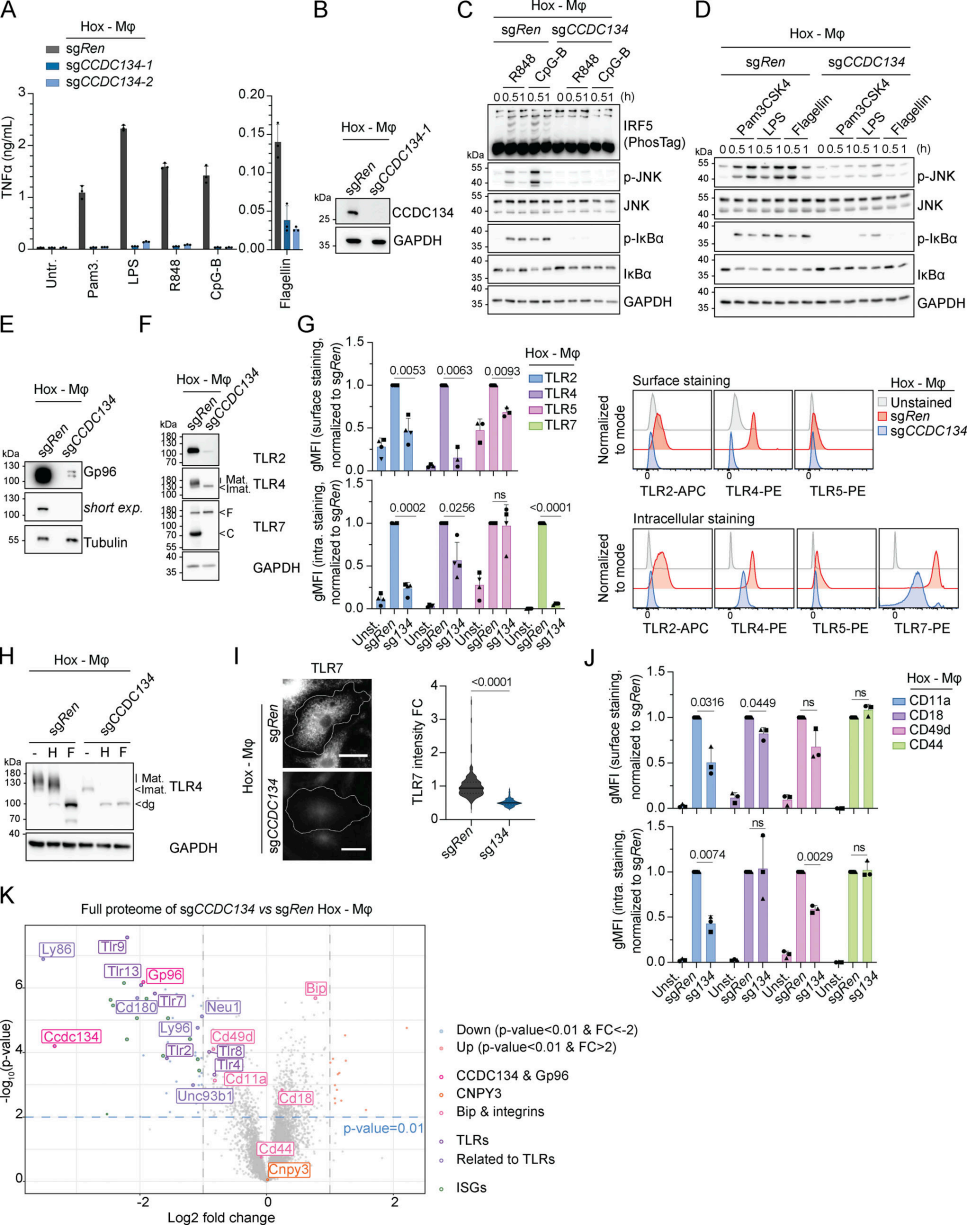
**CCDC134 controls Gp96-dependent maturation and stability of plasma membrane and endolysosomal TLRs. (A)** TNFα production of indicated Hoxb8-macrophages stimulated for 24 h with Pam3CSK4 (Pam3.) (0.1 μg/ml), LPS (10 ng/ml), R848 (0.1 μg/ml), CpG-B (ODN1668, 1 μM), or flagellin (0.1 μg/ml). Untr.: untreated. **(B–D)** Immunoblots of indicated Hoxb8-macrophages untreated (B) or stimulated with R848 (0.1 μg/ml), CpG-B (ODN1668) (1 μM) for 0–1 h (C) or Pam3CSK4 (0.1 μg/ml), LPS (10 ng/ml), and flagellin (0.1 μg/ml) for 0–1 h (D). **(E and F)** Immunoblots of indicated Hoxb8-macrophages. short exp.: short exposure; Mat.: mature; Imat.: immature, F: full length; C: cleaved form. **(G)** Quantification of TLR2, 4, 5, or 7 protein levels in sg*Ren* or sg*CCDC134* Hoxb8-macrophages measured by surface (left upper panel) or surface and intracellular (intra.) (left lower panel) staining and quantified by gMFI (relative to respective sg*Ren*). Representative histograms of surface (right upper panel) or intracellular (right lower panel). gMFI surface staining normalized to sg*Ren* TLR2 P value = 0.0053, TLR4 P value = 0.0063, TLR5 P value = 0.0093; gMFI surface and intracellular staining normalized to sg*Ren* TLR2 P value = 0.0002, TLR4 P value = 0.0256, TLR5 P value = ns, TLR7 P value <0.0001, two-tailed one sample *t* test. **(H)** Immunoblots of lysate from sg*Ren* or sg*CCDC134* Hoxb8-macrophages treated with EndoH (H) or PNGase F (F). Mat.: mature form; Imat.: immature form; dg: deglycosylated. **(I)** Representative image of sg*Ren* or sg*CCDC134* Hoxb8-macrophages stained for TLR7 (scale bar: 5 μm) (left panel) and quantification of TLR7 intensity (right panel). Data are expressed as fold change (FC) and pooled from *n* = 2. Violins show the variation of individual cells across all experiments; P value <0.0001, two-tailed Mann–Whitney test. **(J)** Quantification of CD11a, CD18, CD49d, and CD44 protein levels in sg*Ren* or sg*CCDC134* Hoxb8-macrophages measured by surface (upper panel) or surface and intracellular (intra.) (bottom panel) staining and quantified by gMFI (relative to respective sg*Ren*). Data show mean ± SD from three independent experiments; gMFI surface staining normalized to sg*Ren* CD11a P value = 0.0316, CD18 P value = 0.0449, CD49d P value = ns, CD44 P value = ns; gMFI surface and intracellular staining normalized to sg*Ren* CD11a P value = 0.0074, CD18 P value = ns, CD49d P value = 0.0029, CD44 P value = ns; two-tailed one sample *t* test. **(K)** Volcano plot of quantified proteins in whole proteome of sg*CCDC134* versus sg*Ren* Hoxb8-macrophages (upregulated: red, fold change [FC] > 2 and P value <0.01; downregulated: blue, FC less than −2 and P value <0.01). pink: CCDC134 and Gp96; light pink: Bip and integrins; purple: TLRs; light purple: related to TLRs; green: IFN-stimulated gene proteins (ISGs). In B–F and H, data are representative of two experiments. In A, data show mean ± SD of three stimulation replicates from one experiment representative of three independent experiments. In G–J, data show mean ± SD from four or three independent experiments. Source data are available for this figure: SourceData F6.

To further extend our investigation in primary myeloid cells and to the murine system, we deleted CCDC134 in Hoxb8 immortalized murine myeloid progenitors, which can be differentiated to macrophages (referred as Hoxb8-macrophages), highly similar to primary bone marrow–derived macrophages (Lail et al., 2022), and which express a broader range of TLRs. Remarkably, CCDC134 deficiency impaired responses to agonists of TLR1/2, TLR4, TLR5, TLR7, and TLR9, as assessed by strongly reduced IRF, NF-κB, and MAPK activation as well as virtually blunted TNF production, consistent with a profound impact on Gp96 protein levels (Fig. 6, A–E; and Fig. S5 F). In line with the critical role of Gp96 for TLR folding and trafficking, CCDC134 knockout strongly impacted the stability, maturation, and localization of the assessed TLRs (Fig. 6, F–I). TLR2 protein levels were virtually abolished in immunoblot and undetectable by surface and intracellular FACS staining, as was TLR5 (Fig. 6, F and G). TLR4 was undetectable on the cell surface, and the residual protein detected intracellularly consisted of the immature ER-localized form sensitive to EndoH activity (Fig. 6, F–H). Similarly, while the immature form of TLR7 was still present, the predominant cleaved form was absent in CCDC134-deficient cells, thus resulting in a strong reduction of TLR7 total protein levels, as also confirmed by intracellular FACS and confocal microscopy staining (Fig. 6, F, G, and I). Lastly, we assessed the requirement of CCDC134 for TLR3 responses, which have been shown to be independent of Gp96 and CNPY3 (Liu et al., 2010; Takahashi et al., 2007). In line with this, CCDC134 deficiency in Raw 264.7 macrophages reduced TLR4 and TLR9 responses but did not affect poly(I:C)-induced TNF production, which was blunted by UNC93B1 knockout as expected (Fig. S5, G and H) (Majer et al., 2019b).

Besides TLRs, Gp96 has been reported to affect the stability and trafficking of other client proteins, including a subset of integrins (Liu and Li, 2008; Randow and Seed, 2001; Staron et al., 2010). Consistent with the impact of CCDC134 on Gp96 protein, we observed a reduction in the surface levels of Gp96-dependent integrins CD11a and CD18 (with CD49d showing a similar trend) in CCDC134-deficient Hoxb8-macrophages, while CD44, which does not require Gp96, was not affected (Fig. 6 J and Fig. S5 I). Of note, the effect on integrins was moderate compared to what reported upon Gp96 deficiency (Liu and Li, 2008; Randow and Seed, 2001; Staron et al., 2010), possibly reflecting residual Gp96 expression.

To further and unbiasedly assess the global impact of CCDC134 deficiency, we next performed total proteome analysis on control sg*Ren* and sg*CCDC134* Hoxb8-macrophages (Fig. 6 K and Table S3). Strongly supporting our data, among the most strongly downregulated proteins, we identified Gp96 and multiple TLRs, whose levels of reduction correlated with what observed in our previous assays (Fig. 6, G and K). Moreover, the levels of integrins reflected the results of FACS analysis measuring total (surface and intracellular) proteins (Fig. 6 J and Fig. S5 I). The abundance of other proteins previously described to be affected by Gp96 deficiency was also deregulated in CCDC134 knockouts (including reduction in Ly96/MD2, CD180/RP105, and IGF1) (Hong et al., 2017; Ostrovsky et al., 2010; Wanderling et al., 2007; Weekes et al., 2012) (Fig. 6 K). Lastly, levels of several IFN-inducible proteins were also reduced in CCDC134-deficient cells, possibly suggesting reduced tonic IFN signaling in the absence of TLRs (Fig. S5 J). These data further support that CCDC134 deficiency did not broadly impair protein stability but specifically affected several Gp96-dependent clients.

## Discussion

Altogether, our study uncovered CCDC134 as a critical regulator of TLRs’ folding, maturation, and trafficking, thereby affecting responses of both plasma membrane and endolysosomal TLRs. A notable exception was TLR3, which was not affected by CCDC134 deficiency, in line with previous reports showing that both Gp96 and CNPY3 are not required for its function (Liu et al., 2010; Takahashi et al., 2007). Consistent with our findings, CCDC134 was very recently identified as one of the hits in a loss-of-function screen on TLR4, impairing its glycosylation and surface levels (Lampson et al., 2024). Mechanistically, we showed that CCDC134 is an ER-resident protein whose loss largely phenocopied the effect on TLR7-9 responses observed in cells deficient for CNPY3 (another hit of our screen), the TLR-specific co-chaperone of Gp96. Indeed, CCDC134 interacted with and stabilized Gp96, preventing its hyperglycosylation and degradation through the ERAD pathway. Gp96 has been reported to undergo unusual glycosylation-dependent regulation due to the presence in its sequence of cryptic N-linked glycan acceptor sites (Cherepanova et al., 2019; Dersh et al., 2014). In stress conditions, Gp96 is hyperglycosylated at these sites by the oligosaccharyltransferase complexes STT3A and STT3B resulting in a non-native conformation subjected to a faster degradation (Cherepanova et al., 2019; Dersh et al., 2014). Our data indicate that CCDC134 directly interacts with Gp96 and prevents glycosylation at the cryptic glycan acceptor sites present in its middle region. As the interaction of Gp96 with CCDC134 requires this region, it can be envisioned that CCDC134 binding could prevent the exposure of these sites by promoting or stabilizing the folding of Gp96 and/or by masking them from STT3A and STT3B. Future studies are needed to test this hypothesis and further detail how CCDC134 controls Gp96.

The observation that CCDC134 deficiency affects Gp96 glycosylation and stability reveals that CCDC134 has a different, upstream role than CNPY3, which did not affect Gp96 levels. Indeed, the deletion of CCDC134 impaired the recruitment of both Gp96 and CNPY3 to TLR7. Importantly, results on TLR7 responses indicate that CCDC134 loss impairs TLR function through its effect on Gp96 as Gp96 deletion abrogates the rescuing effect of doxycycline-inducible CCDC134 reconstitution, and, conversely, expression of a Gp96 mutant insensitive to hyperglycosylation, Gp96 N3Q, was able to largely compensate CCDC134 deficiency. While our findings are consistent with CCDC134 deficiency impacting TLRs’ biogenesis through a unique Gp96-dependent mechanism, i.e., defective folding and maturation in the ER resulting in impaired trafficking and reduced receptor levels, different TLRs seem to display various sensitivity to the residual Gp96 levels observed upon CCDC134 loss across the cell types used, with results in THP1 cells suggesting that TLR2 needs a profound defect in Gp96 to be affected. Alternatively, it cannot be excluded that this might result from other, cell-type specific compensatory mechanisms regulating Gp96 in THP1 cells.

The strong impact of CCDC134 loss on Gp96 suggests that its effect could go beyond TLRs and affect most, if not all, Gp96-dependent clients and processes. Indeed, similar as Gp96 deficiency, CCDC134 knockout is embryonically lethal (Wanderling et al., 2007; Yu et al., 2018). Moreover, a recent preprint showed that CCDC134 deficiency impaired the WNT pathway by affecting another of its clients, LRP6, through Gp96 hyperglycosylation and degradation (Ma et al., 2024, *Preprint*). Our data further indicated that CCDC134 deficiency impacted also the levels of Gp96-dependent integrins, even if this reduction was not complete, possibly due to Gp96 residual levels and activity. Interestingly, multiple evidence suggest that Gp96 may utilize different chaperoning mechanisms to fold TLRs compared with integrins. Using different Gp96 mutants to investigate the requirement of ATP binding and hydrolysis, it was shown that TLR folding required both of these Gp96 activities, while integrin chaperoning was dependent only on ATP binding (Randow and Seed, 2001). Moreover, CNPY3 co-chaperone activity was selectively required for TLRs (Liu et al., 2010). This suggests that Gp96 regulates TLRs and integrins using different mechanisms, which could be differently impacted by the Gp96 residual levels observed in CCDC134 knockout cells.

In summary, we uncover that CCDC134 is an essential factor for TLRs’ biogenesis acting through the ER chaperone Gp96. Therefore, the CCDC134–Gp96 pathway controls inflammatory responses induced by both plasma membrane and endolysosomal TLRs, raising the possibility that interfering with this process could be of interest in conditions where multiple TLRs contribute to hyperinflammation.

## Materials and methods

### Antibodies and reagents

#### Antibodies used in western blot

Rabbit anti-phospho-IRF5 (Ser446) (cat. AB309088, RRID: AB_3662099, clone: EPR26132-254, lot: 1054837-15, dilution: 1:1,000; Abcam), rabbit anti-IRF5 (cat. ab181553, RRID: AB_2801301, clone: EPR17067, lot: GR3248905-4 and GR3278824-6, dilution: 1:1,000; Abcam), mouse anti-CCDC134 (cat. sc- 393390, RRID: AB_3662100, clone: E-5, lot: I0717 and G2417, dilution: 1:1,000; Santa Cruz), rat anti-Grp94 (Gp96) (cat. sc-32249, RRID: AB_627676, clone: 9G10, lot: D2122, dilution: 1:1,000; Santa Cruz), rabbit anti-CREB-2 (ATF4) (cat. sc-200, RRID: AB_2058752, clone: C-20, lot: L3113, dilution: 1:1,000; Santa Cruz), mouse anti-GADD153 (CHOP) (cat. sc-575, RRID: AB_631365, clone: F-168, lot: B1411, dilution: 1:1,000; Santa Cruz), mouse anti-GAPDH (cat. sc-365062, RRID: AB_10847862, clone: G-9, lot: I2321, dilution: 1:1,000; Santa Cruz), rabbit anti-CNPY3 (cat. HPA016560, RRID: AB_1858173, lot: A80185, dilution: 1:1,000; Sigma-Aldrich), mouse anti-Flag (cat. F1804, RRID: AB_262044, clone: M2, lot: SLCD6338, dilution: 1:1,000; Sigma-Aldrich), rabbit anti-phospho-IRF3 Ser396 (cat. AP0623, RRID: AB_2771210, lot: 3600002505, dilution: 1:1,000; ABclonal), rabbit anti-IRF3 (cat. 4302, RRID: AB_1904036, clone: D83B9, lot: 7, dilution: 1:1,000; Cell Signaling) mouse anti-IκBα (cat. 4814, RRID: AB_390781, clone: L35A5, lot: 17 and 21, dilution: 1:1,000; Cell Signaling), rabbit anti-phospho-IκBα Ser32 (cat. 2859, RRID: AB_561111, clone: 14D4, lot: 18, dilution: 1:1,000; Cell Signaling), mouse anti-IKKα (cat. 11930, RRID: AB_2687618, clone: 3G12, lot: 5, dilution: 1:1,000; Cell Signaling), rabbit anti-phospho-IKKα/β (Ser176/180) (cat. 2697, RRID: AB_2079382, clone: 16A6, lot: 21, dilution: 1:1,000; Cell Signaling), rabbit anti-phospho-MLKL (Ser358) (cat. 91689, RRID: AB_2732034, clone: D6H3V, lot: 5, dilution: 1:1,000; Cell Signaling), rabbit anti- MLKL (cat. 14993, RRID: AB_2721822, clone: D2I6N, lot: 4, dilution: 1:1,000; Cell Signaling), rabbit anti-SAPK/JNK (cat. 9252, RRID: AB_2250373, lot: 17 and 18, dilution: 1:1,000; Cell Signaling), rabbit anti-phospho-SAPK/JNK Thr183/Tyr185 (cat. 4668, RRID: AB_823588, lot: 15 and 17, clone: 81E11, dilution: 1:1,000; Cell Signaling), rabbit anti-TLR2 (cat.13744, RRID: AB_2798308, clone: E1J2W, lot:1, dilution: 1:1,000; Cell Signaling), rabbit anti-TLR4 (cat. 14358, RRID: AB_2798460, clone: D8L5W, lot: 3, dilution: 1:1,000; Cell Signaling), rabbit anti-TLR7 (cat. 82658, RRID: AB_3662102, clone: E4J3Z, lot: 1, dilution: 1:1,000; Cell Signaling), rabbit anti-TLR7 (cat. 5632, RRID: AB_10692895, clone: D7, lot: 3, dilution: 1:1,000; Cell Signaling), rabbit anti-TLR9 (cat. 5845, RRID: AB_10715078, clone: D2C9, lot: 1, dilution: 1:1,000; Cell Signaling), rabbit anti-BiP (cat. 3177, RRID: AB_2119845, clone: C50B12, lot: 2, dilution: 1:1,000; Cell Signaling), rabbit anti-HA (cat. 3724, RRID: AB_1549585, Clone: C29F4, lot: 10 and 11, dilution: 1:1,000; Cell Signaling), rabbit anti-V5 (cat. 13202, RRID: AB_2687461, clone: D3H8Q, lot: 6, dilution: 1:1,000; Cell Signaling), rabbit anti-GAPDH (cat. 2118, RRID: AB_561053, clone: 14C10, lot: 16, dilution: 1:1,000; Cell Signaling).

#### Reagents and ligands

Tunicamycin was from Enzo (cat. BML-CC104-0010) and NMS-873 from Sigma-Aldrich (cat. SML1128). Z-VAD-FMK (Z-VAD) was from Invivogen (cat. HY-16658B). NSA was from Calbiochem (cat. 480073; Sigma-Aldrich). Dimethyl sulfoxide (DMSO) was from Applichem (cat. A3672.0100). TNFα (cat. 300-01A) and IL-1β (cat. 200-01B) were from Peprotech. R848 (cat. tlrl-r848), CL307 (cat. tlrl-c307), Pam2CKS4 (cat. tlrl-pm2s-1), Pam3CKS4 (cat. tlrl-pms), LPS (cat. tlrl-3pelps), cGAMP (cat. tlrl-nacga23), Poly(I:C) (HMW) (cat. tlrl-pic), C12-iE-DAP (cat. tlrl-c12dap), L18-MDP (cat. tlrl-lmdp), and standard flagellin from *S. typhimurium* (cat. tlrl-stfla) were from Invivogen. CpG-B (ODN2006, and ODN1668) were synthesized by IDT.

### Cell culture

HEK293T cells (cat. CRL-3216), THP1 cells (cat. TIB-202), Raw 264.7 (cat. TIB-71) and Primary Dermal Fibroblast Normal; Human, Neonatal (HDFn) (cat. PCS-201-010) were purchased from ATCC. THP1 DUAL reporter cell lines were obtained from Invivogen (cat. dhpd-nfis). HeLa cells were from DSMZ (cat. ACC-57). CAL-1 cells were kindly provided by T. Maeda (Nagasaki University, Nagasaki, Japan) and U937 by F. Martinon (University of Lausanne, Lausanne, Switzerland). Cell lines were regularly tested for mycoplasma contamination. HEK293T, HeLa, and Raw 264.7 cells were cultured in DMEM (Gibco), THP1, U937, and CAL-1 in RPMI (Gibco), supplemented with 10% (vol/vol) fetal bovine serum (FBS; Gibco) and antibiotics (100 U/ml penicillin, 100 μg/ml streptomycin; Bioconcept). Primary fibroblasts were cultured in fibroblast basal medium (cat. PCS-201-030; ATCC) supplemented with Fibroblast Growth Kit–Low Serum (cat. PCS-201-041; ATCC). The GM-CSF-derived Hoxb8 progenitors were cultivated in DMEM (Gibco), supplemented with 10% (vol/vol) FBS (Gibco), antibiotics (100 U/ml penicillin, 100 μg/ml streptomycin; Bioconcept), 20 ng/ml granulocyte–macrophage colony-stimulating factor (GM-CSF) (cat. 315-03; PeproTech), and 1 µM β-estradiol (Sigma-Aldrich). Cells were incubated at 37°C in a 5% CO_2_ incubator.

### Generation and differentiation of Hoxb8-macrophages

For the generation of Hoxb8 progenitors, mouse bone marrow from wildtype C57BL/6J mice (10 wk old males) was flushed. The progenitors in the bone marrow were enriched by negative depletion of cells expressing TCRβ, CD45R/B220, and CD11b (cat. 109204, 103204, 101204; BioLegend) using biotinylated antibodies and anti-biotin microbeads (cat. 130-090-485; Miltenyi) and cultured in stem cell medium (IMDM [Gibco]) supplemented with 20% FBS. The supernatant contained the following growth factors: stem cell factor (100× dilution), IL-3 (500× dilution), IL-6 (1,000× dilution), all three from HEK293T cells transfected with corresponding plasmids, and antibiotics (100 U/ml penicillin, 100 μg/ml streptomycin; Bioconcept). After 2 days, cells were spinfected with a retrovirus containing an HA-ER-Hoxb8-MSCV-Neo construct and selected for immortalization during subsequent passages in progenitor medium (DMEM [Gibco], supplemented with 10% [vol/vol] FBS [Gibco], antibiotics [100 U/ml penicillin, 100 μg/ml streptomycin; Bioconcept], 20 ng/ml GM-CSF [PeproTech] and 1 μM β-estradiol [Sigma-Aldrich]). Primary murine cells (bone marrow) used to generate the Hoxb8 progenitors were obtained under the guidelines of and with approval from the cantonal veterinary office of the canton of Vaud (Switzerland), license number VD3716.

For the differentiation of Hoxb8 progenitors into macrophages, the progenitor medium was removed by centrifugation at 300 *g* for 4 min, and the cell pellets were washed twice with 1× phosphate-buffered saline (PBS; Gibco). Progenitor cells (2–3 × 10^6^) were seeded in sterile non-tissue culture-treated round petri plates (cat. BH93B-102; Corning) with 10 ml of macrophage medium (DMEM [Gibco], supplemented with 10% [vol/vol] FBS [Gibco], antibiotics [100 U/ml penicillin, 100 μg/ml streptomycin; Bioconcept], and 20 ng/ml macrophage colony-stimulating factor [M-CSF] [PeproTech]). On day 3, half of the medium was exchanged with fresh 5 ml of macrophage medium. On days 5–7, the macrophages were used for experiments.

### PMA-induced differentiation of THP1 and U937 macrophages

Differentiation of THP1 and U937 cells was induced with 10 or 200 nM of PMA (cat. P8139; Sigma-Aldrich), respectively. For ELISA assays, cells were differentiated for 24 h, followed by an additional 24-h treatment with the specified ligands. For western blot analysis, the cells were directly harvested after 48 h of differentiation.

### Plasmids and sgRNAs

Codon-optimized cDNAs for human IRF5, HA-tagged wildtype, hyperglycosylated, or deletion mutant Gp96 were obtained from Genscript. The template for MLKL cloning was previously described (Shkarina et al., 2022). The expression vectors were obtained from the gene expression core facility of the école polytechnique fédérale de Lausanne (EPFL, Lausanne, Switzerland) including human CCDC134 (ID: JF432590) and CNPY3 (ID: EU446764) from the ORFeome Collaboration cDNA Clone and human TLR1-3 (ID: ccsbBroad304_01678, ID: ccsbBroad304_07074, ccsbBroad304_01679) and MyD88 (ID: ccsbBroad304 _10981) from the CCSB-Broad lentiviral expression library (Yang et al., 2011). Plasmids coding for UNC93B1 (ID:132298), Gp96 (ID: 82130), TLR4 (ID: 13086), and TLR5 (ID: 13019) were from Addgene. Plasmids coding for GFP, TLR7, 8, and 9 were kindly provided by G. Superti-Furga (CeMM Research Center for Molecular Medicine of the Austrian Academy of Sciences, Vienna, Austria). TNFR1-flag was kindly provided by P. Schneider (University of Lausanne, Lausanne, Switzerland). Internal Flag-tag CCDC134 and deletion constructs (Δ2–25, Δ226–229, Δ25–57, Δ57–91, Δ91–133, Δ133–156, Δ156–178, Δ178–229, Δ226–229) were generated by site-directed mutagenesis using the PfuUltra High-Fidelity DNA Polymerase AD (cat. 600385-51; Agilent). Stop codon of Gp96 was similarly removed by site-directed mutagenesis. All cDNAs were verified by sequencing and shuttled by Gateway cloning (Invitrogen) to destination vectors: doxycycline-inducible lentiviral gene expression vector pINDUCER21 (ORF-EG) (cat. 46948; Addgene) or pRRL-based lentiviral expression plasmids with a selectable resistance cassette allowing untagged or C-terminal Strep-HA-tagged (SH), V5, or Flag-tagged expression. Gibson assembly cloning (NEB) was used to clone MLKL-IRF5(122–498) construct into pRRL-based lentiviral expression containing a viral T2A sequence and mCherry.

CRISPR–Cas9-based knockout cell line generation was performed using pLentiCRISPRv2 (ID: 52961; Addgene). sgRNA sequences targeting SLC15A4 or TASL as well as a non-targeting, control sgRNA sequence designed against Renilla (sg*Ren*) has been previously described (Heinz et al., 2020). Additional sgRNA sequences used were as follows (5′–3′ orientation):

- *hCCDC134* sgRNA no. 1, F: 5′-CAC​CGG​GAG​CGA​TCC​TGT​CGT​TAG​G-3′, R: 5′-AAA​CCC​TAA​CGA​CAG​GAT​CGC​TCC​C-3′
- *hCCDC134* sgRNA no. 2, F: 5′-CAC​CGA​GTA​ATA​GTG​CAC​AAT​CCT​C-3′, 5′-RAAACGAGGATTGTGCACTATTACTC-3′
- *hCCDC134* sgRNA no. 3, F:5′-CAC​CGA​TCT​GGG​AGC​ACA​TCA​GCA​G-3′, R:5′-AAACCTGCTGATGTGCTCCCAGATC-3′
- *hCCDC134* sgRNA no. 4, F: 5′-CAC​CGC​AGC​ACC​ACA​TCG​CCG​AAG​A-3′, R:5′-AAACTCTTCGGCGATGTGGTGCTGC-3′
- *hCCDC134* sgRNA no. 5, F: 5′-CAC​CGC​CCC​TTG​AGC​ATG​ACA​TCA-3′, R: 5′-AAA​CTG​ATG​TCA​TGC​TCA​AGG​GGC-3′
- h*CNPY3* sgRNA, F: 5′-CAC​CGA​CAC​AAC​CTG​GTA​CAC​AAA​G-3′, R: 5′-AAA​CCT​TTG​TGT​ACC​AGG​TTG​TGT​C-3′
- h*C10orf12* sgRNA, F: 5′-CAC​CGA​CAG​CCA​GAA​AGA​GTA​CTC​G-3′, R: 5′-AAA​CCG​AGT​ACT​CTT​TCT​GGC​TGT​C-3′
- h*ENPP2* sgRNA, F: 5′-CAC​CGC​ACA​CAC​TCT​CCC​TAC​ATG-3′, R: 5′-AAA​CCA​TGT​AGG​GAG​AGT​GTG​TGC-3′
- h*GANAB* sgRNA, F: 5′-CAC​CGA​TTC​CGC​CTT​GAC​CTA​CTA​G-3′, R: 5′-AAA​CCT​AGT​AGG​TCA​AGG​CGG​AAT​C-3′
- *hHsp90b1/Gp96* sgRNA, F: 5′-CAC​CGA​AAC​AGC​AAC​GCT​TCG​GTC​A-3′, R: 5′-AAA​CTG​ACC​GAA​GCG​TTG​CTG​TTT​C-3′
- h*JMJD6* sgRNA, F: 5′-CAC​CGT​TGC​GCA​TTC​AAC​AAA​ACC​A-3′, R: 5′-AAA​CTG​GTT​TTG​TTG​AAT​GCG​CAA​C-3′
- h*MyD88* sgRNA, F: 5′-CAC​CGC​TGC​TCT​CAA​CAT​GCG​AGT​G-3′, R: 5′-AAA​CCA​CTC​GCA​TGT​TGA​GAG​CAG​C-3′
- h*SPCS1* sgRNA, F: 5′-CAC​CGC​TTC​ACC​CCA​GGA​TTA​CAA-3′, R: 5′-AAA​CTT​GTA​ATC​CTG​GGG​TGA​AGC-3′
- h*TMEM8A* sgRNA, F: 5′-CAC​CGG​GCG​AGG​CAG​CAT​TCA​CGC-3′, R: 5′-AAA​CGC​GTG​AAT​GCT​GCC​TCG​CCC-3′
- h*TNFRSF1A* sgRNA, F: 5′-CAC​CGA​AGA​CCA​AAG​AAA​ATG​ACC​A-3′, R: 5′-AAA​CTG​GTC​ATT​TTC​TTT​GGT​CTT​C-3′
- h*UNC93B1* sgRNA, F: 5′-CAC​CGA​CAA​TGA​GGT​TTC​CGC​TCC​G-3′, R: 5′-AAA​CCG​GAG​CGG​AAA​CCT​CAT​TGT​C-3′
- *mCCDC134* sgRNA no. 1, F: 5′-CAC​CGT​CCA​CCA​CGT​GCG​AGA​AAG​C-3′, R: 5′-AAA​CGC​TTT​CTC​GCA​CGT​GGT​GGA​C-3′
- *mCCDC134* sgRNA no. 2, F: 5′-CAC​CGT​GAT​GCC​CCA​GCG​GAT​CAG​G-3′, R: 5′-AAA​CCC​TGA​TCC​GCT​GGG​GCA​TCA​C-3′
- *mUNC93B1* sgRNA, F: 5′-CAC​CGC​GGA​GTG​GTC​AAG​AAC​GTG​C-3′, R: 5′-AAA​CGC​ACG​TTC​TTG​ACC​ACT​CCG​C-3′
- *mCNPY3* sgRNA, F: 5′-CAC​CGT​AGG​TTG​TGC​AGC​GTC​TCA​A-3′, R: 5′-AAA​CTT​GAG​ACG​CTG​CAC​AAC​CTA​C-3′.

### Generation of stable knockout and overexpressing cell lines by lentiviral transduction

For lentiviral gene transduction, HEK293T cells were transfected with the respective lentiviral vectors and packaging plasmids psPAX2 and pMD2.G using PEI (PolySciences). 6 h later, medium was exchanged with fresh DMEM, supplemented with 10% (vol/vol) FBS and antibiotics (100 U/ml penicillin, 100 µg/ml streptomycin; Bioconcept). 48 h after transfection, virus-containing supernatants were collected, filtered through 0.45 μm polyethersulfone filters (Millipore), and supplemented with 5 μg/ml polybrene (Sigma-Aldrich). Cells were infected by spin infection (800 *g*, 45 min, room temperature). 24 h after infection, cells were washed and resuspended in a fresh medium containing appropriate antibiotics, or, in the case of pINDUCER21-based vectors or MLKL-IRF5(122–498)-T2A-mCherry construct, sorted by flow cytometer (BD FACS Aria [BD Biosciences]) based on GFP or mCherry signal, respectively. Selected cell populations were used either for experimental investigations or for isolation of single-cell clones using a flow cytometer (BD FACS Aria [BD Biosciences]), in case of MLKL-IRF5(122–498)-T2A-mCherry or doxycycline-inducible CCDC134 expressing CAL-1 cell clones.

For infection of Hoxb8 progenitors, after filtering, virus-containing supernatants were concentrated by centrifugation (4,000 *g*, 10 min) in an Amicon Ultra 15-ml tube (100 kDa cutoff) (cat. UFC91008; Merck). Concentrated viruses were resuspended in a small volume of fresh DMEM supplemented with 10% (vol/vol) FBS and antibiotics (100 U/ml penicillin, 100 µg/ml streptomycin), 20 ng/ml GM-CSF (Peprotech), 1 μM β-estradiol (Sigma-Aldrich), and 5 μg/ml polybrene (Sigma-Aldrich) and added to the cells. Cells were infected by spin infection (800 *g*, 90 min, room temperature) before being selected, as previously described.

### Genome-scale loss-of-function screen

For the genome-wide screen, 7.5 × 10^7^ of MLKL-IRF5 stably expressing CAL-1 cells were infected with a lentivirus Human CRISPR Knockout Pooled Library (Brunello) (cat. 73179-LV; Addgene) at a coverage of 1,000× and a multiplicity of infection of 0.3. Cells were selected for 4 days with the addition of puromycin (0.5 μg/ml). On day 9 after infection, a first pellet of cells was collected, washed with 1× PBS, and stored at −20°C (sample referred to as uninduced untreated t0). The remaining cells were either kept in normal conditions in culture or induced with doxycycline (0.5 μg/ml) (cat. D9891-1G; Sigma-Aldrich) overnight and stimulated or not with CL307 (cat. tlrl-c307; Invivogen) for 6 h before to be washed and amplified (samples referred to as uninduced untreated, induced untreated, and induced CL307). The treatment induction/stimulation was repeated after 4 days, six times. The cells were induced one last time before being sorted according to mCherry and GFP expression (double positive population). After sorting, the cells were amplified for 5 days and then collected, washed with 1× PBS, and stored at −20°C until genomic DNA extraction. For each condition (uninduced untreated t0, uninduced untreated, induced untreated, and induced CL307) two replicates were performed.

### Screen analysis and hit scoring

Genomic DNA was extracted using the NucleoSpin Blood L kit (cat. 740954.20; Macherey-Nagel) according to the manufacturer’s protocol. Genomic DNA was then purified using the OneStep PCR Inhibitor Removal Kit (cat. D6030; Zymo Research). DNA was quantified using the Qubit 1× dsDNA HS Assay Kit (cat. Q33230; Invitrogen) on a Qubit 4 Fluorometer. The sgRNA cassette was amplified by PCR using Q5 High-Fidelity DNA Polymerase (cat. M0491S; NEB) in single-step PCR reactions of 25 cycles per sample with primers introducing sequencing adapters and barcodes (dual-index sequencing strategy). PCR products were normalized, pooled, and gel-extracted using a SmartPure Gel Kit (cat. SK-GEPU-100; Eurogentec).

#### Sequencing

Sequencing was performed on an Illumina NovaSeq 6000 for 100 cycles single read (version 1.5 flow cell). Sequencing data were demultiplexed using the bcl2fastq2 Conversion Software (version 2.20; Illumina).

#### Bioinformatics

Reads sequences were 3′ and 5′ adapter and quality trimmed using Cutadapt (version 1.3 [Martin, 2011]). Alignment to the Brunello Library was performed with Bowtie (version 1.2 [Langmead et al., 2009]). Read counts per sgRNA and gene were summed up from the alignment output. Statistical analysis utilized Mageck software (version 0.5.7 [Li et al., 2014]) via the “test” function (--norm-method median --adjust-method fdr).

### Protein precipitation in cell culture supernatant, cell lysis, and western blotting

For the detection of secreted proteins in the supernatant, HEK293T cells were transfected with the indicated plasmids encoding for proteins using PEI (PolySciences). After 6 h, cells were washed twice with 1× PBS (Gibco) and incubated overnight with Opti-MEM medium (Gibco). The supernatant was harvested and mixed with chloroform (Biosolve) and methanol (Thermo Fisher Scientific). After centrifugation (18,000 *g*, 10 min, 4°C), the aqueous phase was removed without disturbing the protein layer. After the addition of methanol (Thermo Fisher Scientific), a last centrifugation step was performed (18,000 *g*, 10 min, 4°C) and the protein pellet was resuspended in SDS sample buffer supplemented with 50 mM dithiothreitol (DTT) (cat. A1101.005; BioChemica).

Cells were lysed in radioimmunoprecipitation assay (25 mM Tris, 150 mM NaCl, 0.5% NP-40, 0.5% deoxycholate [wt/vol], 0.1% SDS [wt/vol], pH 7.4), or E1A (50 mM HEPES, 250 mM NaCl, 5 mM EDTA, 1% NP-40, pH 7.4) supplemented with benzonase nuclease (cat. 71205; Merck), complete EDTA-free protease inhibitor cocktail (one tablet for 50 ml) (cat. 11836170001; Roche), and halt phosphatase inhibitor cocktail (cat. 78420; Thermo Fisher Scientific). Lysates were cleared by centrifugation (18,000 *g*, 10 min, 4°C), proteins were quantified with BCA (cat. 23225; Thermo Fisher Scientific) using BSA as standard, and SDS sample buffer supplemented with 50 mM DTT (cat. A1101.005; BioChemica) was added. Cell lysates were resolved by regular or Phos-tag-containing (20 μM, cat. WA3 304-93521; WAKO Chemicals) SDS–PAGE. Following electrophoresis, Phos-tag-containing SDS–PAGE were soaked in transfer buffer with 10 mM EDTA three times for  10 min, rinsed for 10 min in transfer buffer without EDTA, and blotted to nitrocellulose membranes (Amersham, Glattbrugg, Switzerland). Membranes were blocked with 5% non-fat dry milk in TBST and probed with the indicated antibodies. Binding was detected with anti-mouse-HRP secondary antibodies (cat. 115-035-003) or anti-rabbit-HRP secondary antibodies (cat. 111-035-003), from Jackson ImmunoReasearch, using the ECL western blotting system (cat. K-12045-D50; Advansta). In experiments in which multiple antibodies were used, equal amounts of samples were loaded on multiple SDS–PAGE gels and western blots sequentially probed with a maximum of three antibodies.

### Supernatant cell transfer assay

Donor cells (CAL-1 sg*Ren* Ev, sg*CCDC134* Ev or expressing a secreted form of CCDC134 by C-terminal tagging with strep-HA) were cultured in Opti-MEM medium (Gibco). After 24 h of cell culture, supernatants were harvested and added to recipient cells (CAL-1 sg*Ren* Ev, sg*CCDC134* Ev, or expressing a doxycycline-inducible CCDC134 construct [clone 1]) at a final dilution of 1:2. After a subsequent 48-h incubation with the supernatants or induction with doxycycline (0.5 μg/ml; cat. D9891-1G; Sigma-Aldrich), the cells were collected for cell lysis and western blotting as previously described. To confirm the secretion of the C-terminal strep-HA tagged CCDC134, 40 μl of the supernatant collected after 24 h of cell culture was directly mixed with SDS sample buffer containing 50 mM DTT (cat. A1101.005; BioChemica) and used for western blot analysis.

### EndoH and PNGase F treatments

Per sample, 50 μl of cleared lysate was either incubated with or without 0.2 μl of Endo H (cat. P0702S; NEB) or PNGase F (cat. P0704S; NEB) for 30 min at 37°C. Samples were analyzed by western blotting.

### Co-immunoprecipitation

For co-immunoprecipitation assays, HEK293T cells were transfected with the indicated plasmids encoding for tagged proteins using PEI (PolySciences). After 6 h, the media was exchanged, and, 24 h post-transfection, cells were harvested for the following steps. 2–4 × 10^7^ CAL-1 cells were used. The cells were lysed using E1A buffer (50 mM HEPES pH 7.4, 250 mM NaCl, 5 mM EDTA, 1% NP40) supplemented with Roche EDTA-free protease inhibitor cocktail (1 tablet per 50 ml) for 10 min on ice. Lysates were cleared and quantified as described above. After the removal of whole cell lysate as input, the remaining material was subjected to immunoprecipitation with anti-V5 agarose (cat. A7345; Sigma-Aldrich), StrepTactin Sepharose resin (cat. 2-1201-002; IBA Lifesciences), or anti-Flag M2 affinity gel (cat. A2220; Sigma-Aldrich) 3 h or overnight at 4°C. After washing of beads three times with E1A buffer, proteins were eluted with SDS sample buffer supplemented with 50 mM DTT (cat. A1101.005; BioChemica), 5 mM biotin (cat. B4501; Sigma-Aldrich), or ammonium hydroxide elution buffer (pH 11–12), respectively, and analyzed by SDS-PAGE and immunoblotting.

### Production of recombinant proteins and in vitro binding assay

For the production of recombinant proteins, human N-terminally Flag-tagged CCDC134 and C-terminally HA-tagged Gp96 were cloned into the pET28a expression vector (C-terminal His-tag) and expressed in ClearColi expression system in LB medium upon IPTG (0.5 mM; cat. 162423; Biosolve) induction overnight at 22°C. Bacteria were then lysed by sonication, cleared by centrifugation and the lysates were loaded on Ni-Sepharose 6 Fast Flow columns (cat. 17-5318-01; Cytiva), washed with increasing concentration of imidazole (10–30 mM) and finally eluted (20 mM Tris pH 8.0, 300 mM NaCl, 10% glycerol, 300 mM imidazole). Samples of Gp96-HA were further purified on Akta pure FPLC system using Superdex 200 10/300 column (cat. GE28-9909-44; Cytiva).

Before the co-immunoprecipitation assay, Flag-CCDC134 and Gp96-HA were preincubated overnight at 4°C to allow the formation of a complex. After resuspension in E1A buffer (50 mM HEPES pH 7.4, 250 mM NaCl, 5 mM EDTA, 1% NP40), the samples were incubated with anti-HA agarose beads (cat. A2095; Sigma-Aldrich) for 3 h at 4°C. Following the washing of beads three times with E1A buffer, proteins were eluted with SDS sample buffer supplemented with 50 mM DTT (cat. A1101.005; BioChemica) and analyzed by SDS-PAGE and immunoblotting.

### Immunoprecipitation and mass spectrometry

5 × 10^7^ of CAL-1 cells were used for immunoprecipitation of Flag-CCDC134 as described above. An additional preclearing step using Sepharose 6B (cat. 6B100; Sigma-Aldrich) for 1 h at 4°C was performed before incubation with the anti-Flag M2 affinity gel (cat. A2220; Sigma-Aldrich). Eluted samples were snap-frozen in liquid nitrogen before further use. Four replicates were performed for mass spectrometry analysis.

#### Digestion

Eluates from Flag beads were frozen and dried in a SpeedVac system. The material was resuspended in 40 µl miST lysis buffer (1% sodium deoxycholate, 100 mM Tris pH 8.6, and 10 mM DTT) and denatured at 75°C for 10 min. Samples were diluted 1:1 (vol:vol) with water and reduced disulfides were alkylated by adding 0.25 vol of 160 mM chloroacetamide (32 mM final) and incubating for 45 min at room temperature in the dark. Samples were adjusted to 3 mM EDTA and digested with 0.5 µg Trypsin/LysC mix (cat. V5073; Promega) for 1 h at 37°C, followed by a second 1-h digestion with an additional 0.5 μg of proteases. To remove sodium deoxycholate, two sample volumes of isopropanol containing 1% TFA were added to the digests, and the samples were desalted on a strong cation exchange (SCX) plate (Oasis MCX; Waters Corp.) by centrifugation. After washing with isopropanol/1% TFA, peptides were eluted in 200 μl of 80% MeCN, 19% water, 1% (vol/vol) ammonia, and dried by centrifugal evaporation.

#### LC-MS analysis (Exploris 480)

Tryptic peptide mixtures were injected on an Ultimate RSLC 3000 nanoHPLC system interfaced via a nanospray Flex source to a high-resolution Orbitrap Exploris 480 mass spectrometer (Thermo Fisher Scientific). Peptides were loaded onto a trapping microcolumn Acclaim PepMap100 C18 (20 mm × 100 μm ID, 5 μm; Dionex) before separation on a C18 custom packed column (75 μm ID × 45 cm, 1.8 μm particles, Reprosil Pur, Dr. Maisch) using a gradient from 4% to 90% acetonitrile in 0.1% formic acid for peptide separation (total time: 140 min). Full MS survey scans were performed at 120,000 resolution. A data-dependent acquisition (DDA) method controlled by Xcalibur software (Thermo Fisher Scientific) was used that optimized the number of precursors selected (“top speed”) of charge 2+ to 5+ while maintaining a fixed scan cycle of 2 s. Peptides were fragmented by higher energy collision dissociation with a normalized energy of 30% at 15,000 resolution. The window for precursor isolation was 1.6 m/z units around the precursor, and the selected fragments were excluded for 60 s from further analysis.

#### MS data processing

Data files were analyzed with MaxQuant 2.4.7.0 (Cox and Mann, 2008) incorporating the Andromeda search engine (Cox et al., 2011). Cysteine carbamidomethylation was selected as fixed modification while methionine oxidation and protein N-terminal acetylation were specified as variable modifications. The sequence databases used for searching were the human reference proteome based on the UniProt database (https://www.uniprot.org, RefProt_Homo_sapiens_20230301, version of January 2023, containing 81,856 sequences) and a “contaminant” database containing the most usual environmental contaminants and enzymes used for digestion (keratins, trypsin, etc.) (Frankenfield et al., 2022). Mass tolerance was 4.5 ppm on precursors (after recalibration) and 20 ppm on MS/MS fragments. Both peptide and protein identifications were filtered at 1% false discovery rate (FDR) relative to hits against a decoy database built by reversing protein sequences. The match between runs function was not used. iBAQ values were used for processing.

All subsequent analyses were done with a custom software tool. Contaminant proteins were removed and intensity iBAQ values were log_2_-transformed (Schwanhausser et al., 2011). After assignment to groups, only proteins quantified in at least two samples of one group were kept. After missing values imputation (based on a down-shifted normal distribution), *t* tests were carried out with Benjamini-Hochberg FDR correction for multiple testing (Q-value threshold <0.05).

### Hoxb8-macrophages full proteome

5 × 10^6^ Hoxb8-macrophages were washed twice with 1× PBS before being snap-frozen in liquid nitrogen for further use.

#### Protein extraction and digestion

Washed cell pellets were lysed in 250 μl miST lysis buffer (1% sodium deoxycholate, 100 mM Tris pH 8.6, 10 mM DTT) and heated for 10 min at 75°C. Genomic DNA was disrupted by tip sonication (2 × 15 s). Samples were digested following a modified version of the iST method (named miST method) (Kulak et al., 2014). Based on tryptophane fluorescence quantification (Wisniewski and Gaugaz, 2015), 50 μg of proteins at 1 μg/μl in miST lysis buffer (1% sodium deoxycholate, 100 mM Tris pH 8.6, 10 mM DTT) was transferred to new tubes. Samples were heated for 5 min at 95°C, diluted 1:1 (vol:vol) with water containing 4 mM MgCl_2_ and benzonase (cat.70746, dilution: 1:100 of stock = 250 U/μl; Merck), and incubated for 15 min at room temperature to digest NA. Reduced disulfides were alkylated by adding 0.25 vol of 160 mM chloroacetamide (32 mM final) and incubating for 45 min at room temperature in the dark. Samples were adjusted to 3 mM EDTA and digested with 1.0 μg Trypsin/LysC mix (cat. V5073; Promega) for 1 h at 37°C, followed by a second 1 h digestion with an additional 0.5 μg of proteases. To remove sodium deoxycholate, two sample volumes of isopropanol containing 1% TFA were added to the digests, and the samples were desalted on an SCX plate (Oasis MCX; Waters Corp.) by centrifugation. After washing with isopropanol/1% TFA, peptides were eluted in 200 μl of 80% MeCN, 19% water, 1% (vol/vol) ammonia, and dried by centrifugal evaporation.

#### LC-MS analysis

LC-MS/MS analyses were carried out on a TIMS-TOF Pro (Bruker) mass spectrometer interfaced through a nanospray ion source (“captive spray”) to an EvoSep One LC system. Peptides were separated on a reversed-phase, 15-cm C18 column (150 μm ID, 1.5 μm particles, EV1137; EvoSep) at a flow rate of 0.220 μl/min with a 15-samples per day method (total method time: 88 min, all solvents contained 0.1% formic acid). Protein identification and quantitation were done by data-independent acquisition (DIA) using a standard method as reported previously (Meier et al., 2020). Per cycle, the mass range 400–1,200 m/z was covered by a total of 32 windows, each 25 Th wide and a 1/k0 range of 0.3. The collision energy was ramped linearly based uniquely on the 1/k0 values from 20 (at 1/k0 = 0.6) to 59 eV (at 1/k0 = 1.6). Two windows were acquired per TIMS scan (100 ms) so that the total cycle time was 1.7 s.

#### MS data processing

Raw Bruker MS data were processed directly with Spectronaut 18.6 (Biognosys) in DIA direct mode, i.e., without library construction based on DDA. For identification, peptides of 7–52 AA length were considered, cleaved with trypsin/P specificity and a maximum of two missed cleavages. Carbamidomethylation of cysteine (fixed), methionine oxidation, and N-terminal protein acetylation (variable) were the modifications applied. Mass calibration was dynamic and based on a first database search. The Pulsar engine was used for peptide identification using the mouse (mus musculus) reference proteome database based on the UniProt database (https://www.uniprot.org, February 2024 version, containing 54,830 sequences) and a “contaminant” database containing the most usual environmental contaminants and enzymes used for digestion (keratins, trypsin, etc.) (Frankenfield et al., 2022). Protein inference was performed with the IDPicker algorithm. Spectra, peptide, and protein identifications were all filtered at 1% FDR against a decoy database. Peptide quantitation was based on XIC area, for which a minimum of one and a maximum of three (the three best) precursors were considered for each peptide, from which the median value was selected. Quantities for protein groups were derived from inter-run peptide ratios based on MaxLFQ algorithm (Cox et al., 2014). Global normalization of runs/samples was done based on the median of peptides.

All subsequent analyses were done with a custom software tool. Contaminant proteins were removed and intensity iBAQ values were log_2_-transformed (Schwanhäusser et al., 2011). After assignment to groups, only proteins quantified in at least two samples of one group were kept. After missing values imputation (based on a down-shfited normal distribution), *t* tests were carried out with Benjamini-Hochberg FDR correction for multiple testing (Q-value threshold <0.05).

### THP1 DUAL cell reporter assay

THP1 DUAL cells (1 × 10^5^ cells per 96 well) were stimulated for 24 h with different ligands as indicated. Poly(I:C) was complexed with lipofectamine (1:1 ratio) (cat. 11668019; Invitrogen) in Opti-MEM medium (Gibco) for 20 min before stimulation of cells. Cell culture supernatant was collected, cleared of residual cells by centrifugation, and analyzed for NF-κB and ISRE reporter activity according to the manufacturer’s instructions. Assays were detected with SpectraMax iD3 microplate reader and results were analyzed in Prism version 10.

### Enzyme-linked immunosorbent assay

Cells were stimulated with the indicated ligands. Cell supernatants were harvested 24 h later and cleared from residual cells by centrifugation. ELISA kits for human IL-6 (cat. 88-7066-88), human TNFα (cat. 88-7346-77), and mouse TNFα (cat. 88-7324-88) were from Invitrogen. Kits for human IFN-β (cat. DY814-05) and mouse IFN-β (cat. DY823405) were from R&D Systems. Cell supernatants were diluted except for measurement of human IFN-β, which was performed using undiluted supernatants. All ELISA experiments were performed according to the manufacturer’s instructions. For detection of human IFN-β, supernatants were incubated overnight at 4°C.

### Quantitative real-time PCR (qPCR)

Cells were collected and total RNAs were isolated using a Quick-RNA Miniprep Kit (cat. R1055; Zymo Research). Reverse transcription was performed using RevertAid First Strand cDNA Synthesis Kit (cat. K1622; Thermo Fisher Scientific) using oligo (dT) primers. Real-time PCR was performed using LightCycler 480 KAPA SYBR FAST (cat. KK4611; Roche). Samples were analyzed on LightCycler 480 (Roche). Data were analyzed and Ct values were calculated using LightCycler Software version 1.5 (Roche). Results were obtained using the 2^−ΔΔCt^ method, using *HPRT1* as a reference. The following gene-specific primers were used:

- Doxycycline-inducible hCCDC134 (does not amplify endogenous ccdc134 as reverse primer target integrated vector sequence) R: 5′-GAG​AGA​AGA​AGA​GAA​ACG​CCG-3′, R: 5′-CCA​CTT​TGT​ACA​AGA​AAG​TTG​GGT​AC-3′
- hHSP90B1(/GP96) F: 5′-CTA​CAA​ATT​ACT​ATG​CGA​GTC​AGA​AG-3′, R: 5′-CAT​CTT​CCT​TAA​TTC​GTC​GAA​GC-3′
- hHPRT1 R: 5′-AGA​CTT​TGC​TTT​CCT​TGG​TCA​G-3′, R: 5′-CCA​ACA​AAG​TCT​GGC​TTA​TAT​CC-3′.

### Cell viability and flow cytometry

MLKL-IRF5 expressing CAL-1 cells were treated as indicated and cell viability was estimated based on morphologic changes using forward scatter (FSC) versus side scatter (SSC) gating or by live/dead staining using a Live/dead fixable near-IR fluorescent reactive dye (cat. L34975; Thermo Fisher Scientific) diluted 1:1,000 in FACS Buffer (2% FBS, 2 mM EDTA, PBS) on ice for 10 min. Samples were analyzed on cytoflex S (Beckman Coulter), and data were analyzed using FlowJo (version 10.9.0; BD Biosciences).

For the surface staining, cells were washed using 1× PBS and resuspended in FACS buffer (2% FBS, 2 mM EDTA, 1× PBS) with TruStain FcX (anti-mouse CD16/32) antibody (cat. 101320, dilution: 1:500; BioLegend) 10 min prior incubation with indicated antibodies and a live/dead fixable near-IR fluorescent reactive dye (cat. L34975, dilution: 1:1,000; Thermo Fisher Scientific) for 30–60 min. Cells were washed and fixed using a fixation buffer (cat. 420801; BioLegend) for 10 min before being analyzed.

For intracellular staining, after washing with 1× PBS, cells were resuspended in FACS buffer (2% FBS, 2 mM EDTA, 1× PBS) with a live/dead fixable near-IR fluorescent reactive dye (cat. L34975A, dilution: 1:1,000; Thermo Fisher Scientific) for 15 min. Cells were washed, fixed using a fixation buffer (cat. 420801; BioLegend) for 10 min, and permeabilized with an intracellular staining permeabilization wash buffer (cat. 421002; BioLegend). Cells were incubated with a TruStain FcX (anti-mouse CD16/32) antibody (cat. 101320, dilution: 1:500; BioLegend) 10 min before incubation with indicated antibodies diluted in FACS buffer (2% FBS, 2 mM EDTA, PBS) for 30–60 min, on ice. Cells were washed before being analyzed. All incubation steps were performed on ice. Samples were analyzed on BD LSRII flow cytometer and data were analyzed using FlowJo (version 10.9.0; BD Biosciences).

For flow cytometry, TLR2 APC (cat. 130-120-138, RRID: AB_2752006, clone: REA109, dilution: 1:50) was from Miltenyi, TLR4 PE (cat. 117605, RRID: AB_313792, clone: MTS510, dilution: 1:20), TLR5 PE (cat. 148107, RRID: AB_2890696, clone: ACT5, dilution: 1:20), TLR7 PE (cat. 160003, RRID: AB_2860749, clone: A94B10, dilution: 1:50), CD11a PE (cat. 101107, RRID: AB_312780, clone: M17/4, dilution: 1:200), CD18 FITC (cat. 101405, RRID: AB_312814, clone: M18/2, dilution: 1:200), CD44 PE (cat. 103007, RRID: AB_312958, clone: IM7, dilution: 1:400), and CD49d APC (cat. 103621, RRID: AB_2565776, clone: R1-2, dilution: 1:200) were from BioLegend.

### Confocal microscopy and unbiased quantification of co-localization

HeLa cells were seeded in 24-well plates on coverslips coated with poly-L-lysin hydrobromide (cat. P6282; Sigma-Aldrich). Cells were transfected with the indicated plasmids encoding for proteins using PEI (PolySciences). After 6 h, the media was exchanged to fresh one, and 24 h after transfection, cells were washed with 1× PBS, fixed with 2% paraformaldehyde (PFA) (Sigma-Aldrich) for 20 min, and permeabilized and blocked in blocking solution (PBS, 0.3% saponin [Sigma-Aldrich], 10% FBS) for 1 h. Afterward, cells were stained for 1 h at room temperature with the indicated primary antibodies in a blocking solution. Antibodies used for immunofluorescence analysis in this study are mouse anti-CCDC134 E-5 (cat. 393390, RRID: AB_3662100, dilution: 1:250; Santa Cruz), rabbit anti-calreticulin (cat. 2907, RRID: AB_303402, dilution: 1:500; Abcam), rabbit anti-V5 D3H8Q (cat. 13202, RRID: AB_2687461, dilution: 1:1,000; Cell Signaling), and mouse anti-Flag M2 (cat. F1804, RRID: AB_262044, dilution: 1:500; Sigma-Aldrich). After three washes with blocking solution, cells were stained with goat anti-mouse IgG (Alexa Fluor 488, cat.115-545-166, dilution: 1:1,000; Jackson ImmunoResearch) and goat anti-rabbit (Alexa Fluor 568, cat. A1101, dilution: 1:1,000; Thermo Fisher Scientific) antibodies for 1 h at room temperature. Cells were then washed once in the blocking solution. Nuclear counterstaining was performed with DAPI (cat. R37606; Invitrogen). After three washes with blocking buffer and one wash with 1× PBS, coverglasses were mounted onto microscope slides using ProLong Gold (cat. P10144; Invitrogen) antifade reagent. Images were acquired on a confocal laser scanning microscope (Zeiss LSM 880; Carl Zeiss) and analyzed using ZEN 2.3 (Carl Zeiss).

To determine the degree of colocalization between CCDC134 deletion mutant constructs (labeled with Alexa Fluor 488) and calreticulin (labeled with Alexa Fluor 568), the composite image was split into its individual channels (green and red signal), which were then used as input for the colocalization threshold plugin in ImageJ (version 1.53t). For each field of view, the Rcoloc value was plotted as an individual data point. Of note, this value is identical to the Pearson R value (no threshold) provided by the Coloc2 plugin. Detailed documentation of the functionality of both algorithms is provided online. The statistically significant discrepancy of the colocalization values between the wildtype and the mutant was determined by two-tailed Mann-Whitney test.

### Super-resolution structured illumination microscopy (SIM)

#### Immunofluorescence

On day 6 of differentiation, Hoxb8-macrophages were plated onto 15-mm round glass coverslips (no. 15H) in a 12-well plate with 1.5 × 10^5^ cells per coverslip. Samples were washed once with 1× PBS and fixed with 2% (vol/vol) PFA-PBS (Thermo Fisher Scientific) for 15 min at room temperature. Next, macrophages were permeabilized with a 0.5% (vol/vol) saponin solution PBS for 5 min at room temperature, followed by a 10-min incubation with a quenching solution (50 mM ammonium chloride in PBS + 0.1% saponin [Roth]) to minimize PFA autofluorescence. Between each indicated step, the cells were thoroughly rinsed with 1× PBS. Slides were blocked in 3% BSA and 0.1% saponin in 1× PBS with 4% horse serum (cat. 26050-070; Gibco) for 1.5 h at room temperature. Primary antibody labeling using mouse anti-TLR7 (cat. MABF2273, RRID: AB_3662104, clone: A94B16, lot: 3956068, dilution: 1:200; Millipore) was performed overnight at 4°C (1% BSA, 0.1% saponin in 1× PBS). After washing three times with 1× PBS, the samples were incubated for 1 h with secondary antibodies goat anti-mouse IgG (H+L) (Alexa Fluor 488, cat. A-11029, lot: 2179204, dilution: 1:1,000; Invitrogen) in 3% BSA and 0.1% saponin in 1× PBS. The macrophages were washed three times in 1× PBS, incubated with Phalloidin-iFluor405 (cat. AB176752, lot: 1038197-11, dilution: 1:4,000; Abcam) for 5 min, rinsed again with 1× PBS, and mounted using ProLong Glass antifade mountant (cat. P36982; Invitrogen).

#### Image acquisition

Epifluorescence microscopy was performed on a Zeiss Elyra 7 lattice SIM microscope equipped with 405 nm (50 mW), 488 nm (500 mW), 561 nm (500 mW), and 642 nm (500 mW) lasers for excitation. Images were acquired using a 63×/1.6 oil immersion objective (1.6× magnification) and a pco.edge 4.2 sCMOS (scientific complementary metal-oxide semiconductor) camera. Images were visualized and contrast adjusted using FIJI/ImageJ.

#### Image analysis

Quantification of TLR7 protein levels in sg*Ren* and sg*CCDC134* macrophages by epifluorescence microscopy was executed in FIJI/ImageJ. In the first step, macrophages were manually segmented using the Phalloidin staining to identify the cell outlines. The corresponding regions of interest (ROIs) were saved and then used to determine the mean fluorescence intensity (mean gray value) for each cell.

### Multiple sequence alignment and secondary structure prediction

Protein sequences were extracted from the UniProt database (UniProt Consortium, 2019) and aligned using Clustal Omega (default settings) (Madeira et al., 2022). Secondary structure prediction was performed for the human TASL sequence using JPred4 (Drozdetskiy et al., 2015).

### Statistical analysis

No statistical methods were used to predetermine the sample size. Data are represented as individual values, mean ± SD, or box and violin plots as described in the figure legends. Group sizes (*n*), the applied statistical test as well as the exact P values are indicated in the figure legends. Statistical analyses were performed using the Prism software (GraphPad).

### Online supplemental material

Fig. S1 complements Fig. 1 by offering further information on the MLKL-IRF5 reporter system and on the loss-of-function genetic screen. Fig. S2 relates to Fig 2 and provides additional details on CCDC134 protein, its localization and effect on its loss on TLR7-9 cleavage. Together with Fig. 3, Fig. S3 shows that CCDC134 binds to Gp96 and that its deletion leads to GP96 hyperglycosylation. Fig. S4 supports data in Fig. 4 which detail the domains of CCDC134 required to rescue Gp96 levels in CCDC134 knockout cells. Fig. S5 complements Figs. 5 and 6 by providing additional information on the impact of CCDC134 loss on plasma membrane TLR biogenesis, localization, and function. Table S1 provides information related to the loss-of-function genetic screen. Table S2 details the results of the interaction proteomic experiments using CCDC134 as bait, while Table S3 lists the results of the total proteome analysis on control sg*Ren* and sg*CCDC134* Hoxb8-macrophages.

## Supplementary Material

Table S1 provides information related to the loss-of-function genetic screen.

Table S2 details the results of the interaction proteomic experiments using CCDC134 as bait.

Table S3 lists the results of the total proteome analysis on control sg*Ren* and sg*CCDC134* Hoxb8-macrophages.

SourceData F1 is the source file for Fig. 1.

SourceData F2 is the source file for Fig. 2.

SourceData F3 is the source file for Fig. 3.

SourceData F4 is the source file for Fig. 4.

SourceData F5 is the source file for Fig. 5.

SourceData F6 is the source file for Fig. 6.

SourceData FS1 is the source file for Fig. S1.

SourceData FS2 is the source file for Fig. S2.

SourceData FS3 is the source file for Fig. S3.

SourceData FS4 is the source file for Fig. S4.

SourceData FS5 is the source file for Fig. S5.

## Data Availability

The mass spectrometry proteomics data underlying Fig. S3 B, Table S2, Fig. 6 K, and Table S3 have been deposited to the ProteomeXchange Consortium via the PRIDE partner repository with the dataset identifiers PXD053008 and PXD053042. All other data are available in the article and its supplementary materials and are also available upon request from the corresponding author.
