# Supplementary figures and images for: CCDC134 controls TLR biogenesis through the ER chaperone Gp96
Source: J Exp Med. 2024 Dec 10;222(3):e20240825. doi: 10.1084/jem.20240825 (PMC11629888; doi:10.1084/jem.20240825)

SourceDataF1F

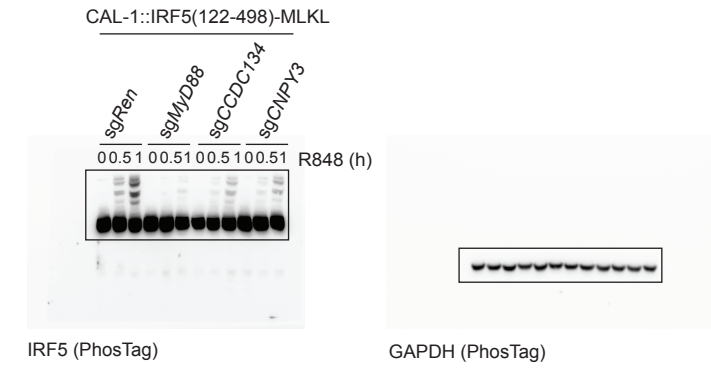

SourceDataF1G

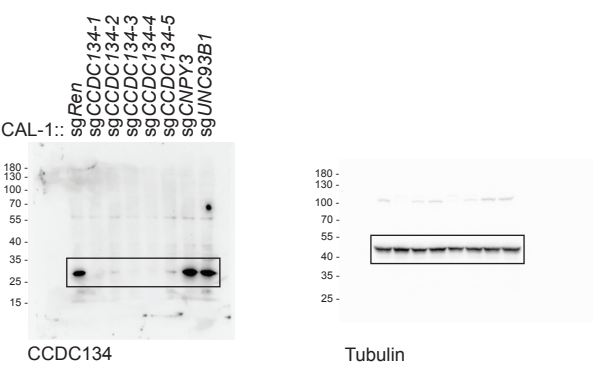

SourceDataF1I

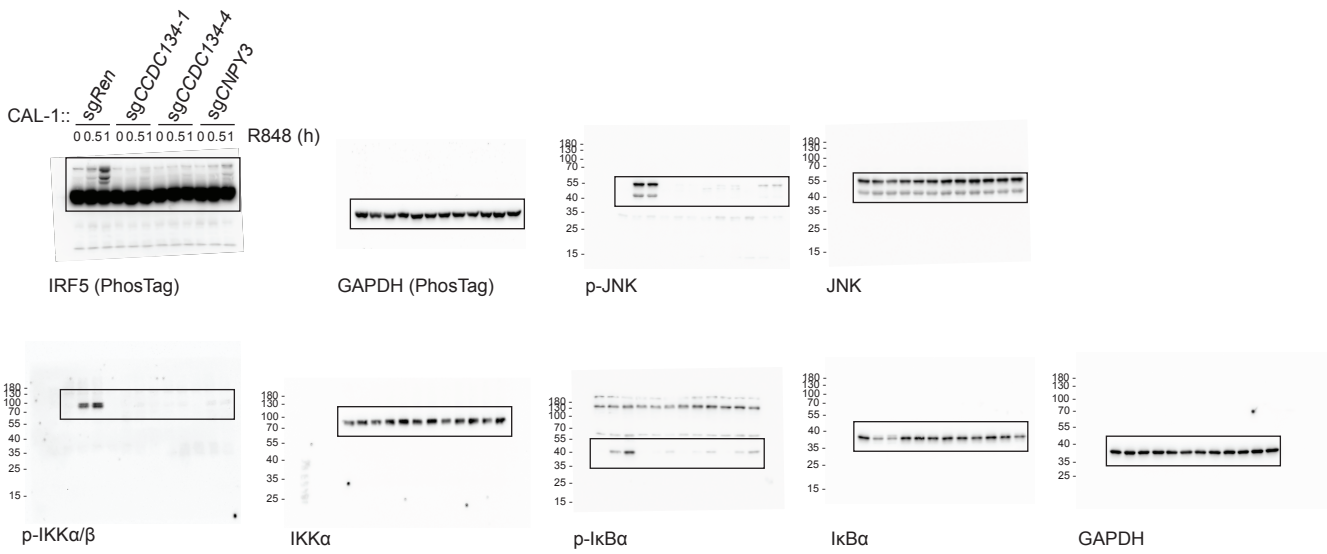

SourceDataF1J

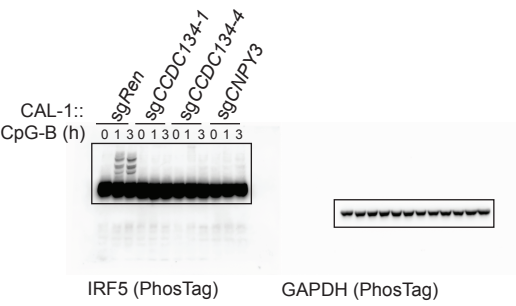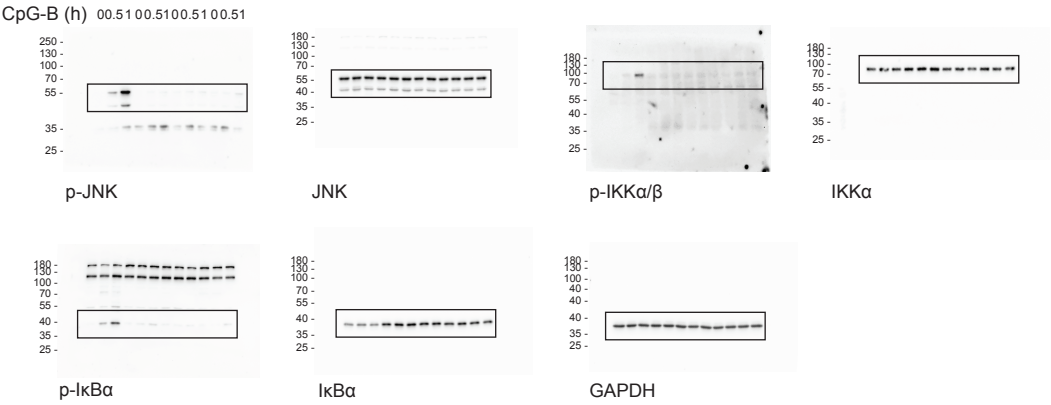

SourceDataF1K

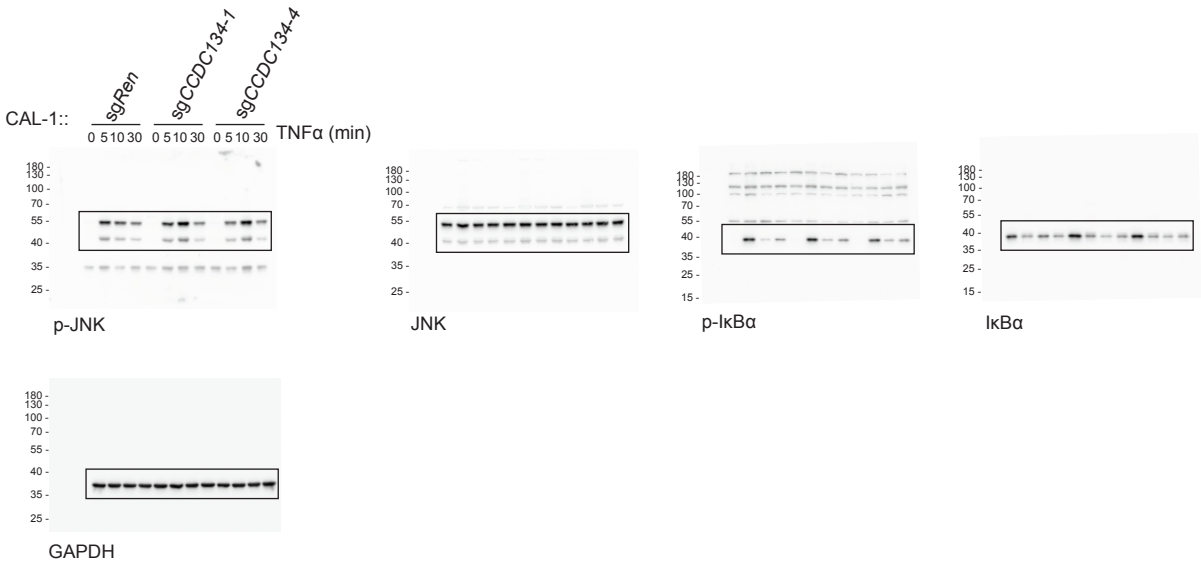

Supplement: SourceData F1 — is the source file for Fig. 1. [file jem_20240825_sourcedataf1.pdf]

SourceDataF2C

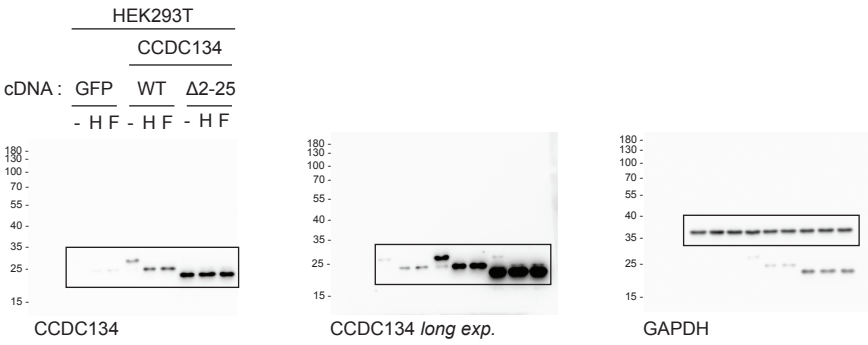

SourceDataF2D

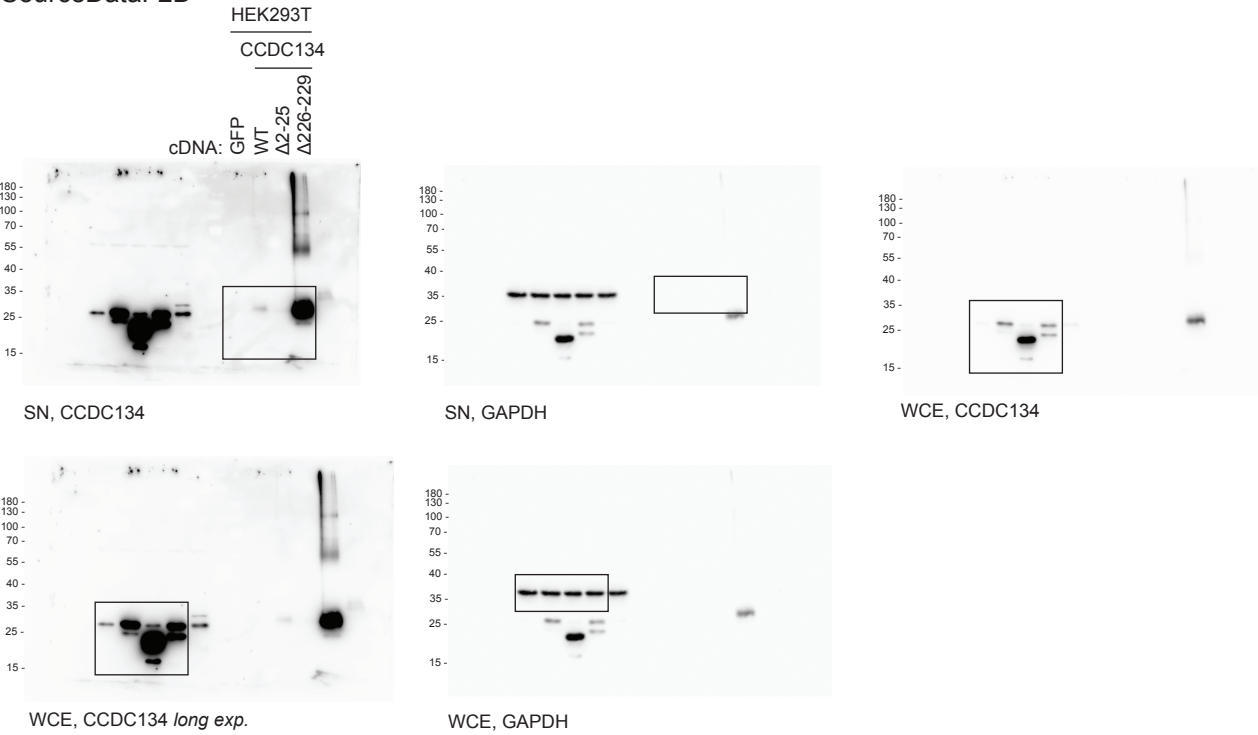

SourceDataF2F

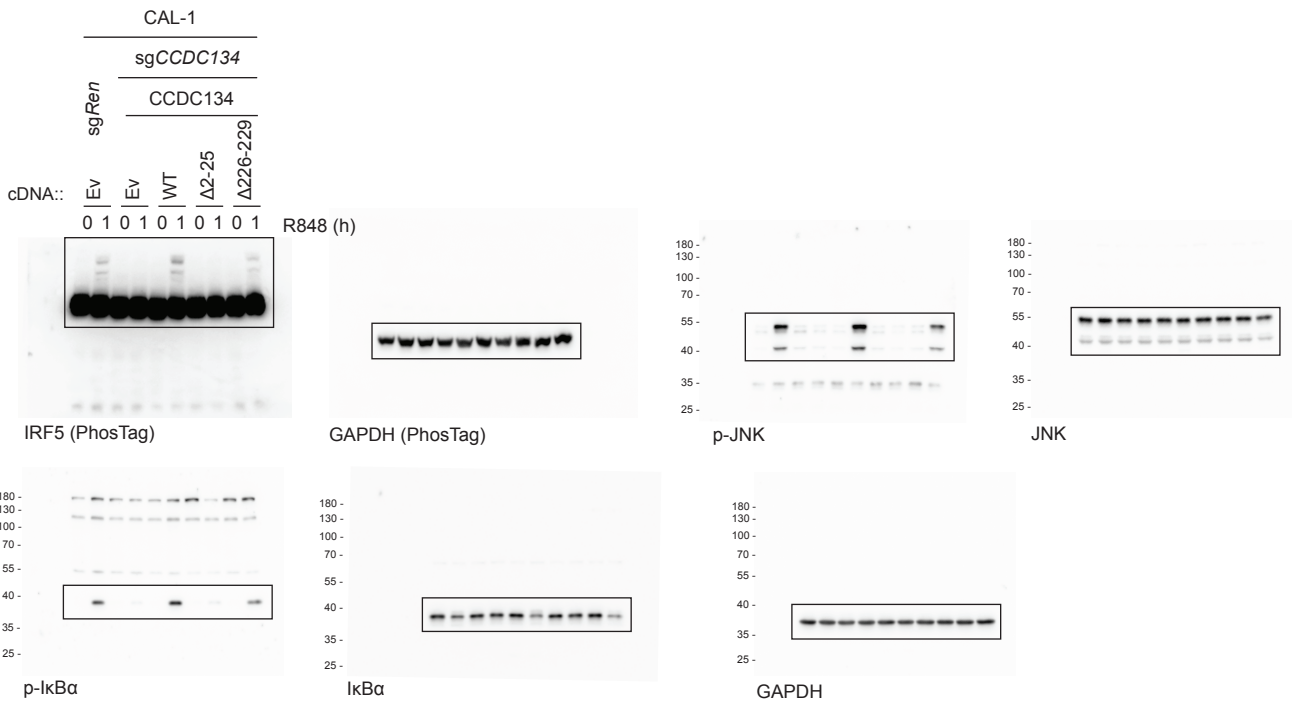

SourceDataF2G

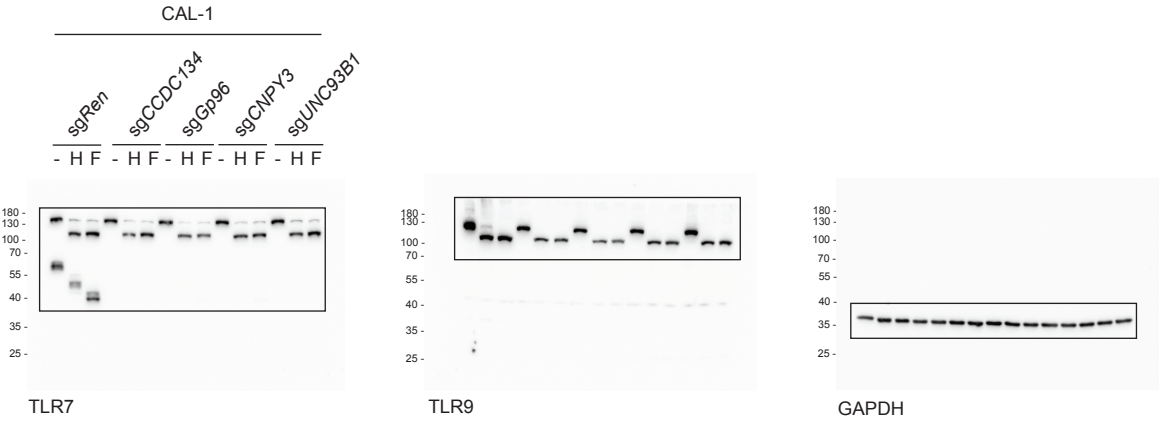

SourceDataF2H

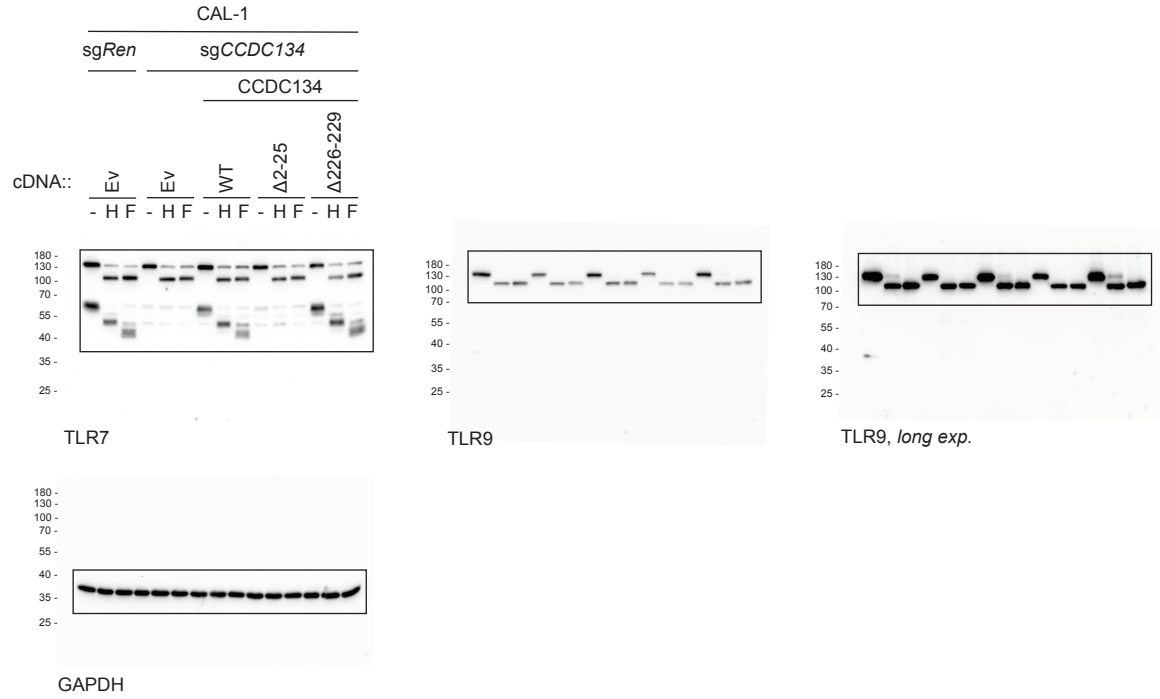

Supplement: SourceData F2 — is the source file for Fig. 2. [file jem_20240825_sourcedataf2.pdf]

SourceDataF6B

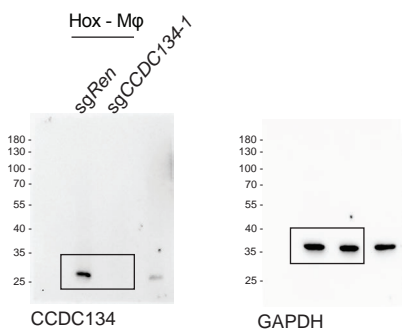

SourceDataF6C

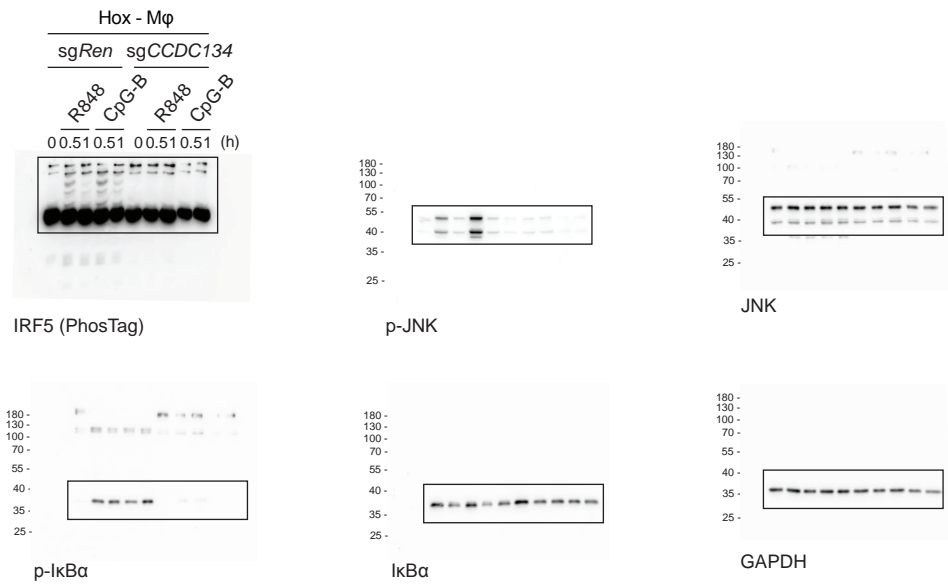

SourceDataF6D

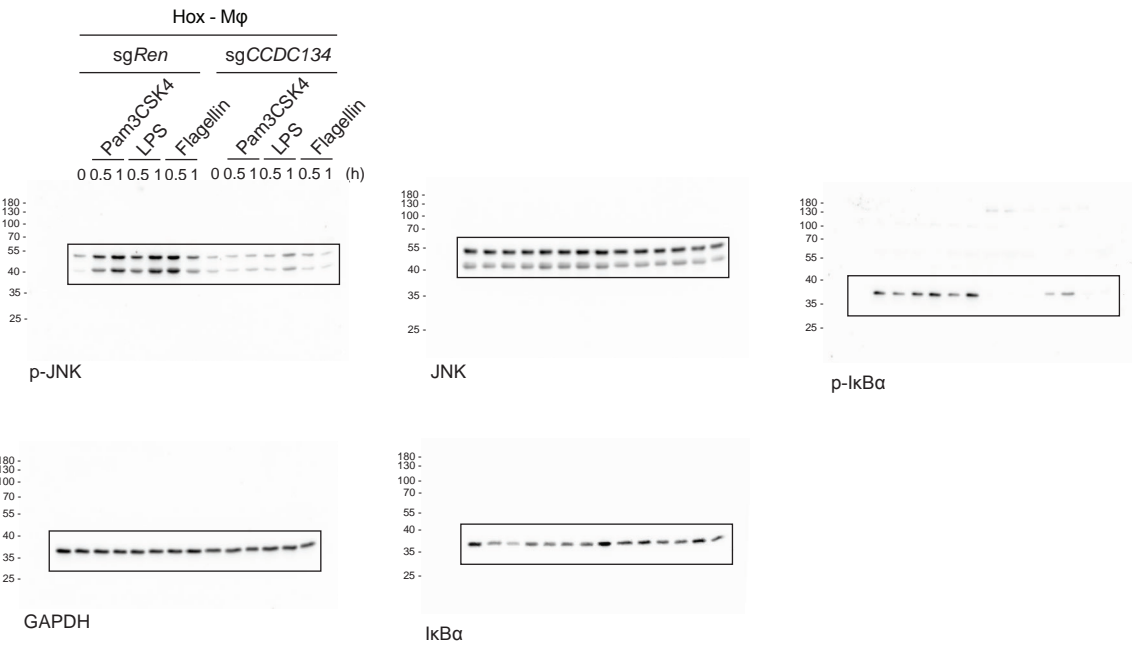

SourceDataF6E

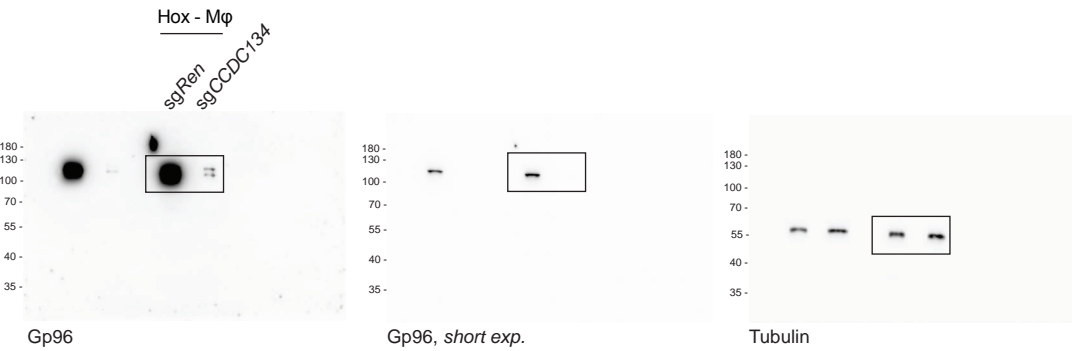

SourceDataF6F

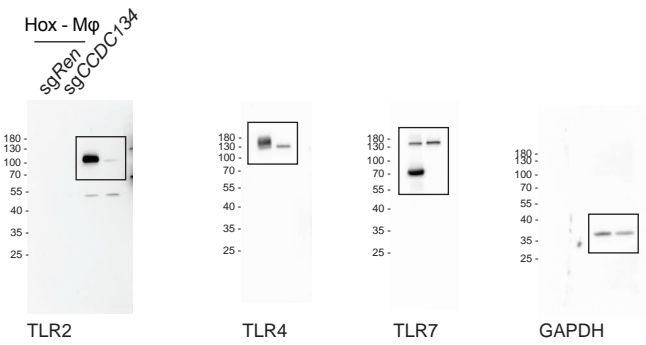

SourceDataF6H

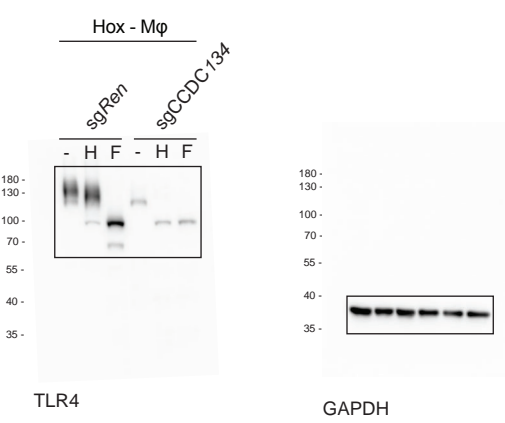

Supplement: SourceData F6 — is the source file for Fig. 6. [file jem_20240825_sourcedataf6.pdf]

SourceDataFS1D

CAL-1::  
IRF5(122-498)-MLKL  
(Clone)  
0 0.5 1 R848 (h)  
- + - + - + Dox.

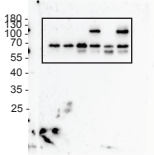

p-IRF5

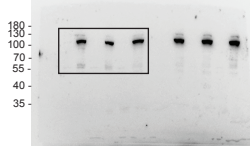

p-MLKL

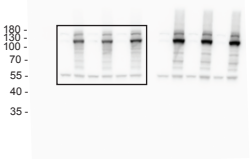

MLKL

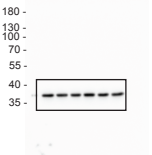

GAPDH

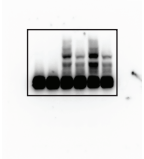

IRF5 (PhosTag)

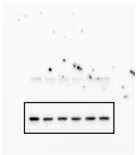

GAPDH (PhosTag)

Supplement: SourceData FS1 — is the source file for Fig. S1. [file jem_20240825_sourcedatafs1.pdf]

SourceDataFS3A

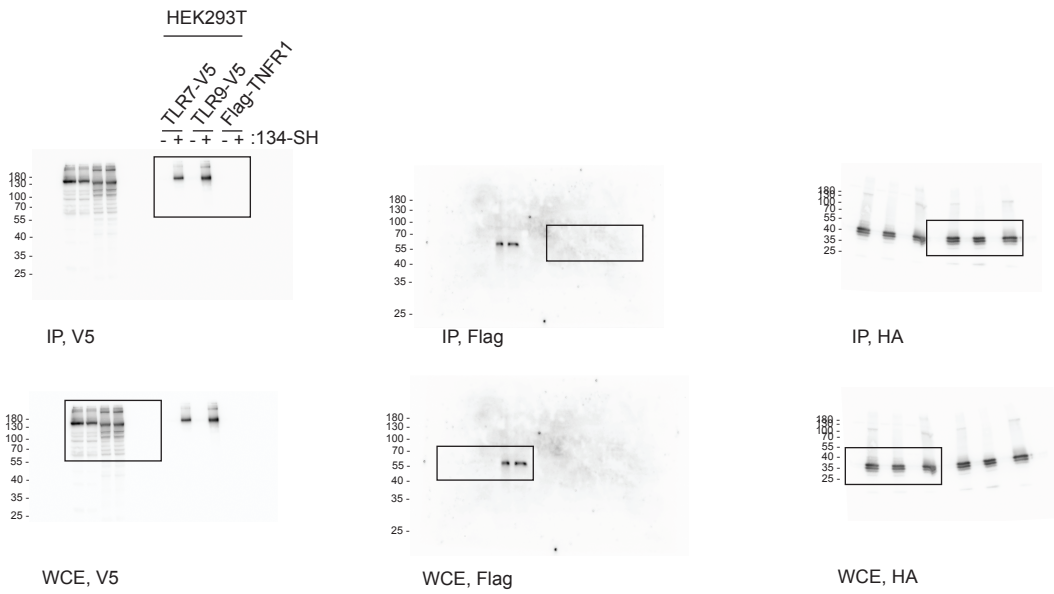

SourceDataFS3C

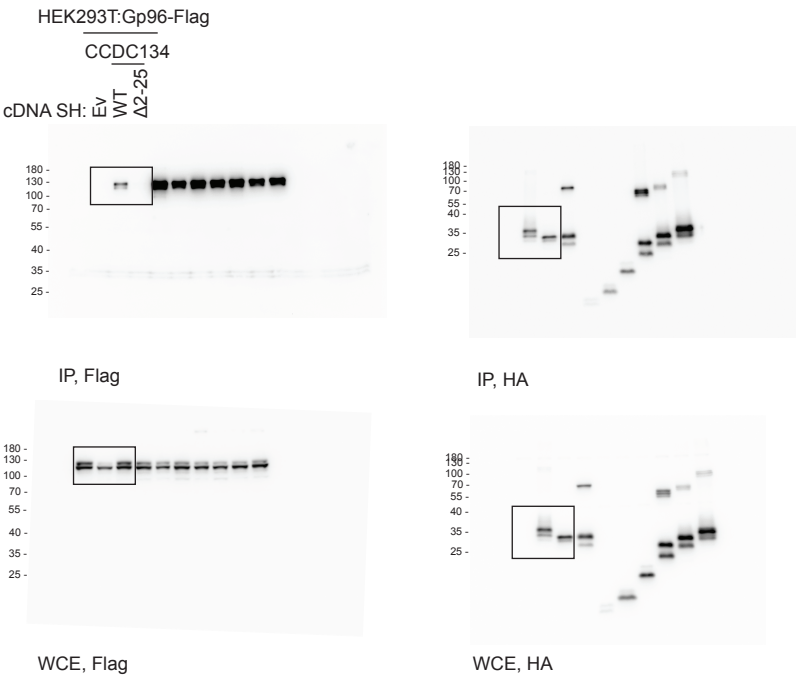

SourceDataFS3E

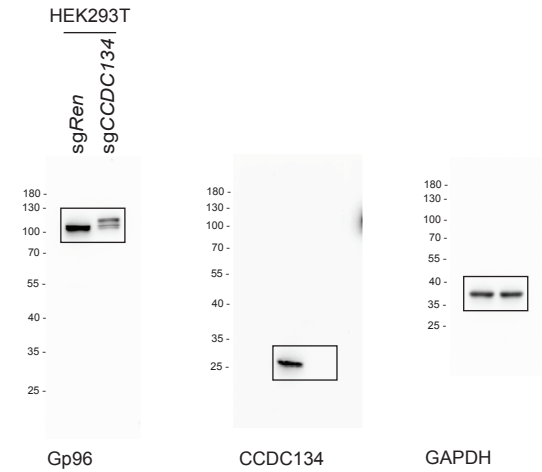

SourceDataFS3F

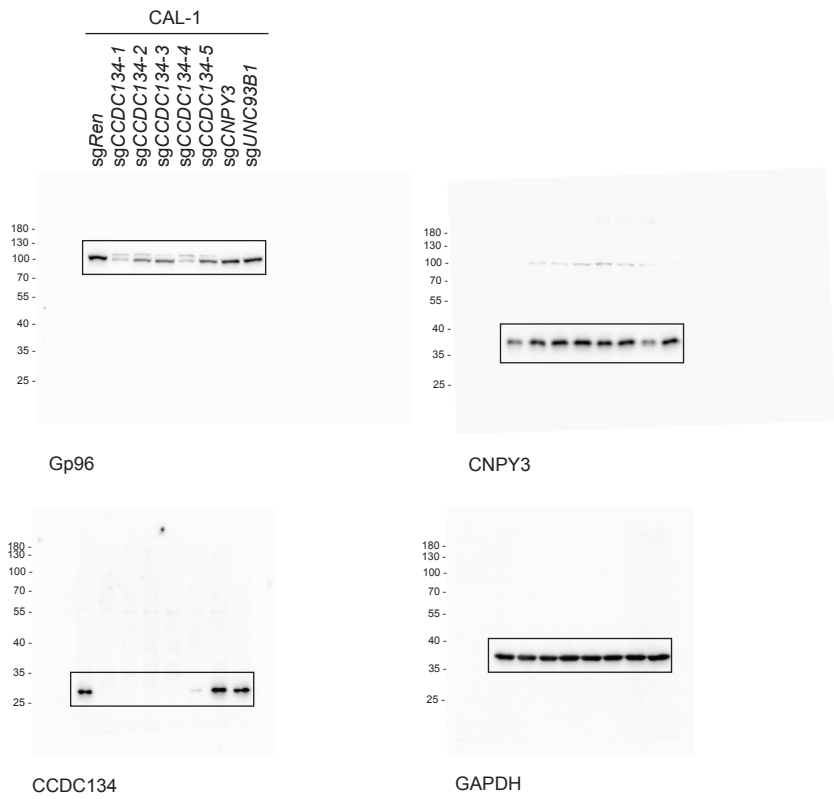

SourceDataFS3H

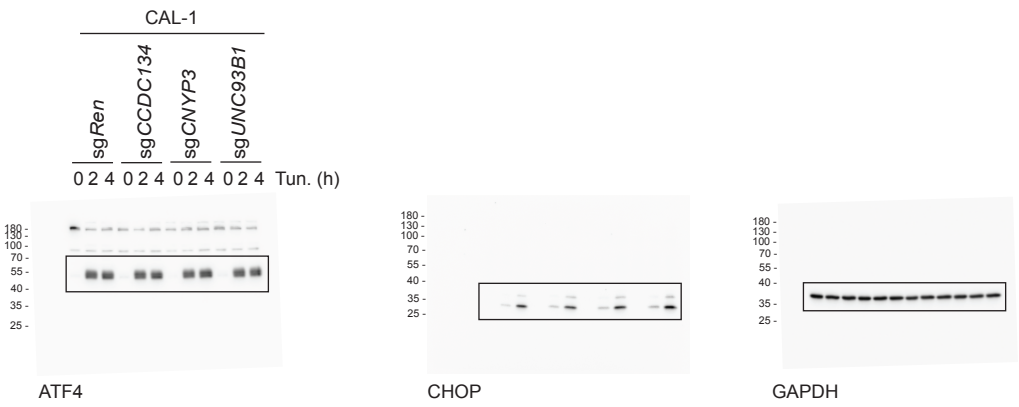

Supplement: SourceData FS3 — is the source file for Fig. S3. [file jem_20240825_sourcedatafs3.pdf]

|       |      | CAL-1     |        |         |  |  |
|-------|------|-----------|--------|---------|--|--|
| sgRen |      | sgCCDC134 |        |         |  |  |
| EV    | EV   | Δ25-57    | Δ57-91 | Δ91-133 |  |  |
| - HF  | - HF | - HF      | - HF   | - HF    |  |  |

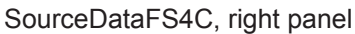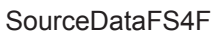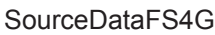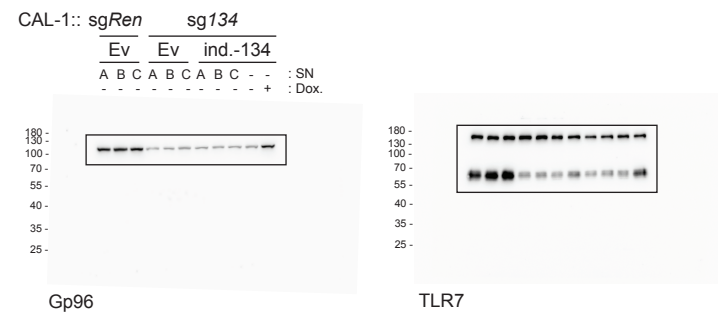

Supplement: SourceData FS4 — is the source file for Fig. S4. [file jem_20240825_sourcedatafs4.pdf]

SourceDataFS5E

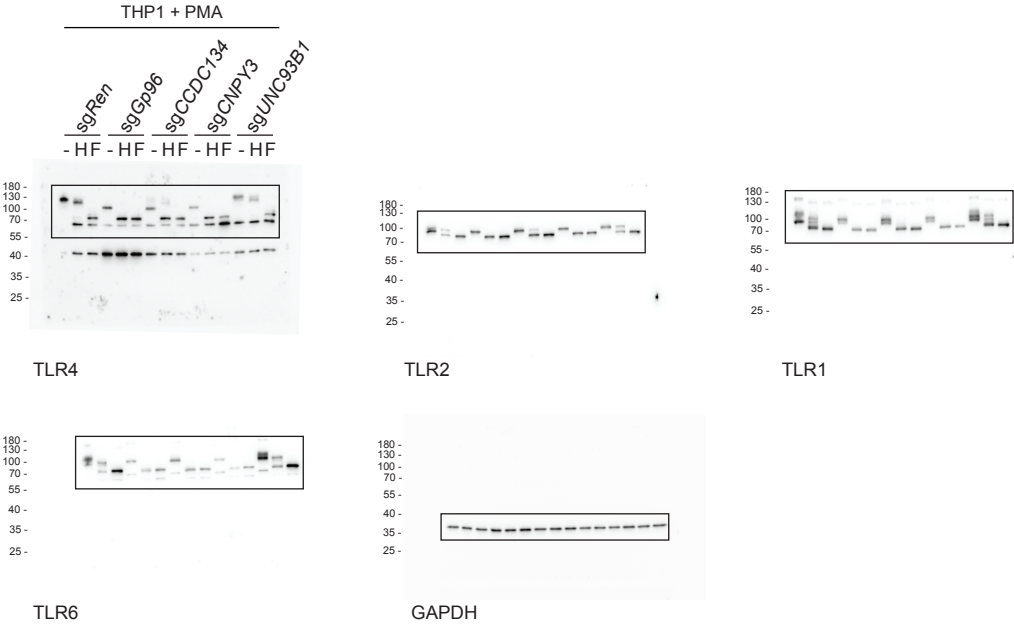

SourceDataFS5F

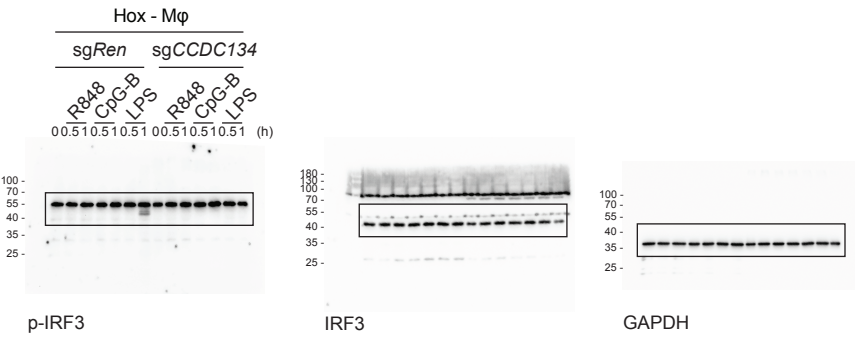

SourceDataFS5G

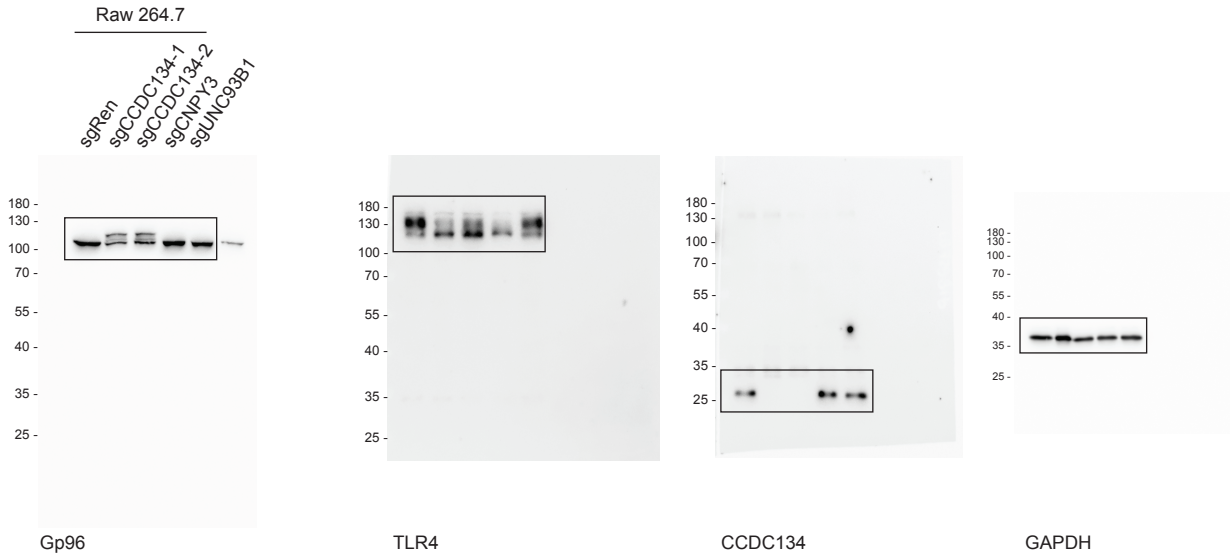

Supplement: SourceData FS5 — is the source file for Fig. S5. [file jem_20240825_sourcedatafs5.pdf]
