# Supplementary material for: CCDC134 controls TLR biogenesis through the ER chaperone Gp96
Source: J Exp Med. 2024 Dec 10;222(3):e20240825. doi: 10.1084/jem.20240825 (PMC11629888; doi:10.1084/jem.20240825)

SourceDataF3A

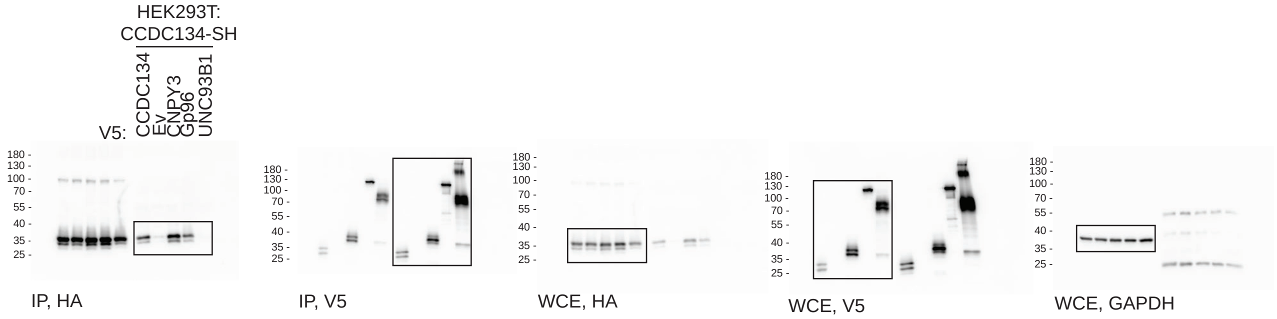

SourceDataF3B

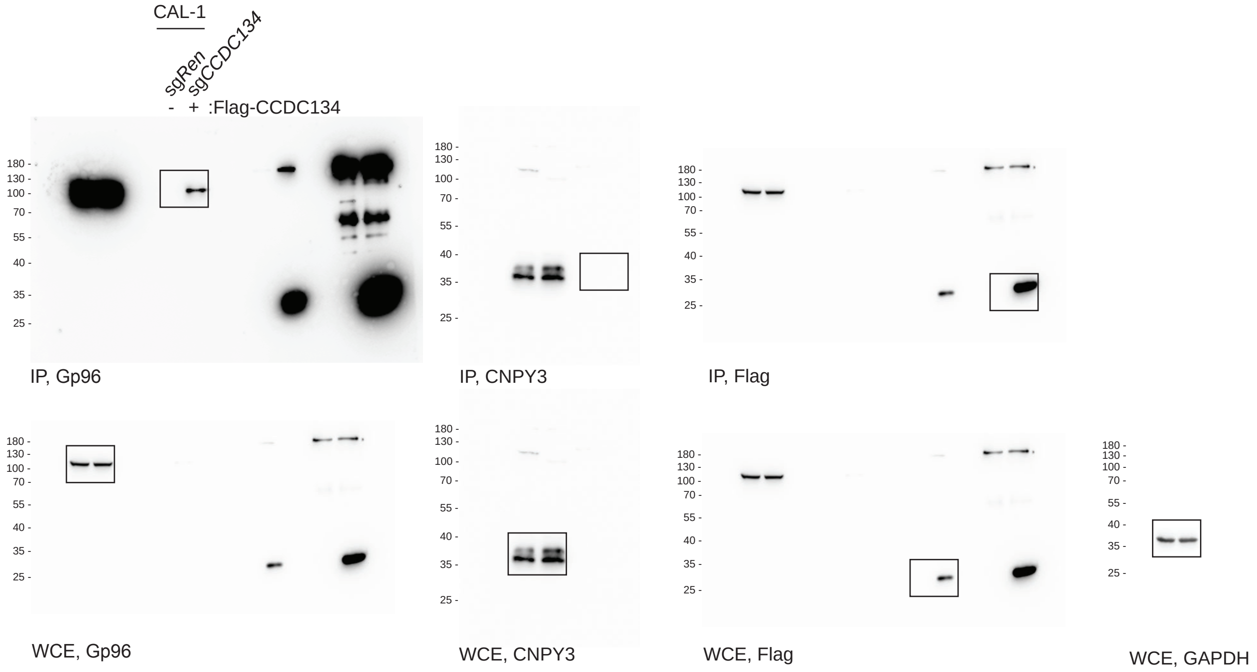

SourceDataF3C

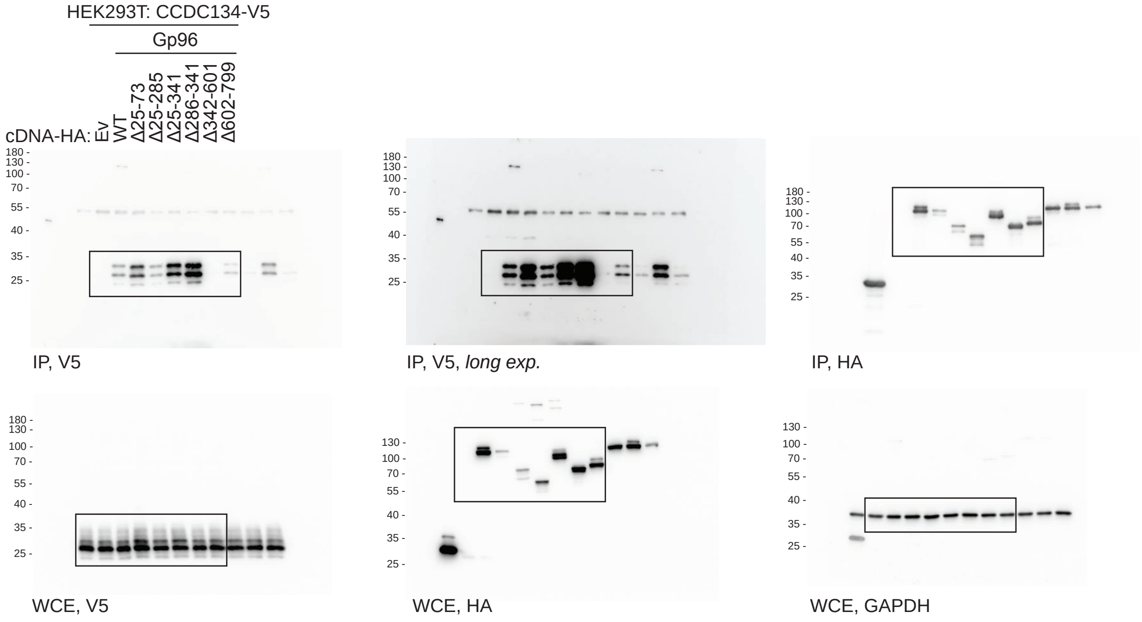

SourceDataF3D

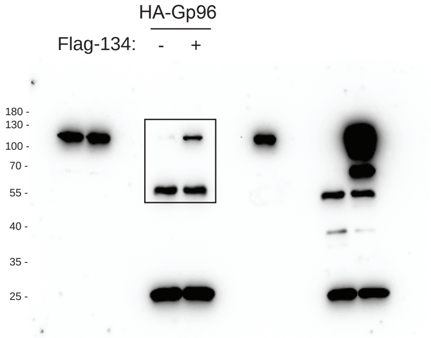

IP, Gp96

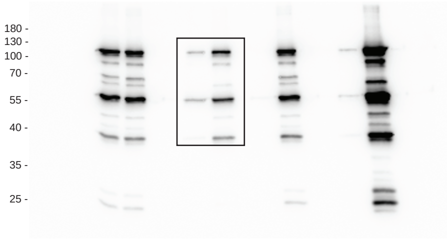

IP, HA

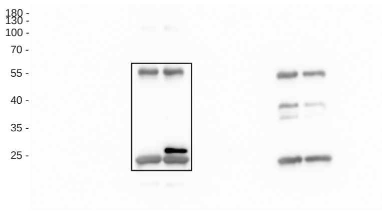

IP, Flag

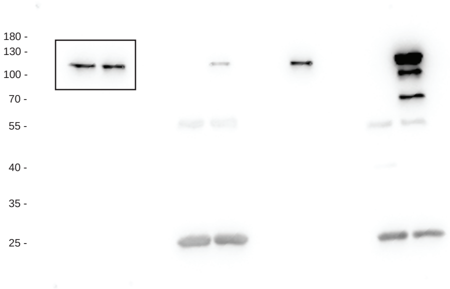

WCE, Gp96

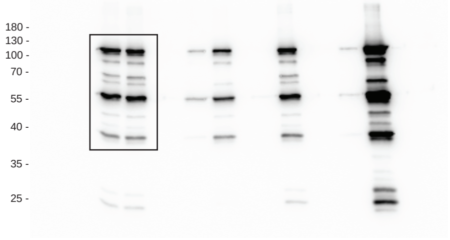

WCE, HA

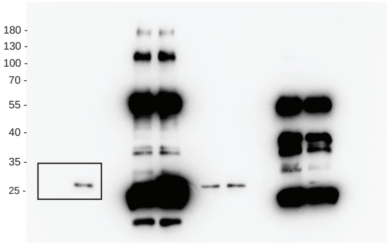

WCE, Fag

SourceDataF3E

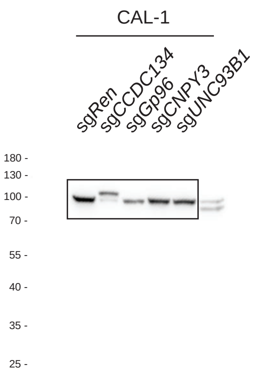

Gp96

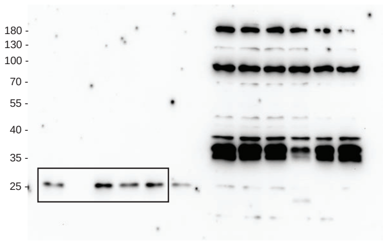

CCDC134

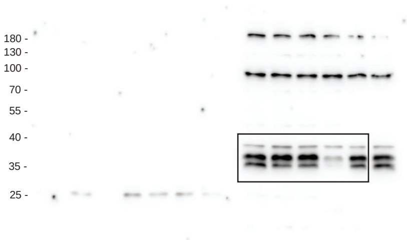

CNPY3

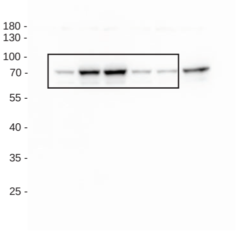

BiP

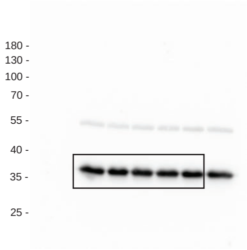

GAPDH

SourceDataF3G

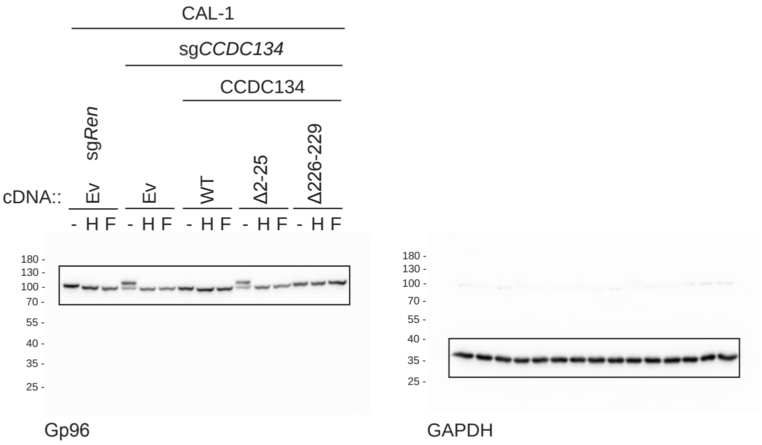

SourceDataF3H

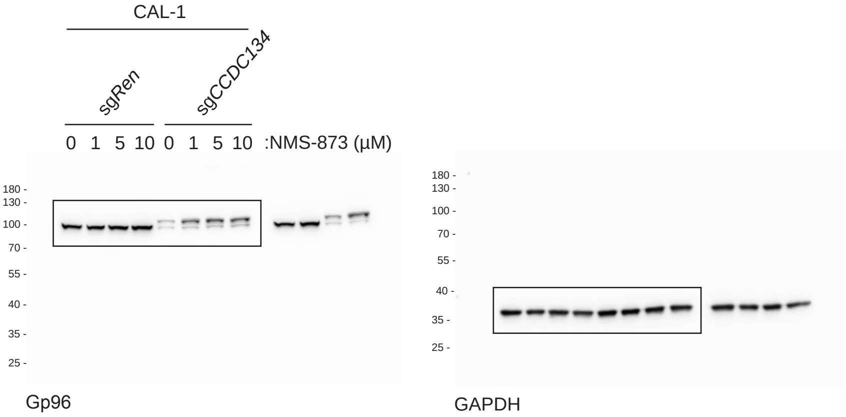

Supplement: SourceData F3 — is the source file for Fig. 3. [file jem_20240825_sourcedataf3.pdf]
