# Supplementary material for: CCDC134 controls TLR biogenesis through the ER chaperone Gp96
Source: J Exp Med. 2024 Dec 10;222(3):e20240825. doi: 10.1084/jem.20240825 (PMC11629888; doi:10.1084/jem.20240825)

SourceDataF4A

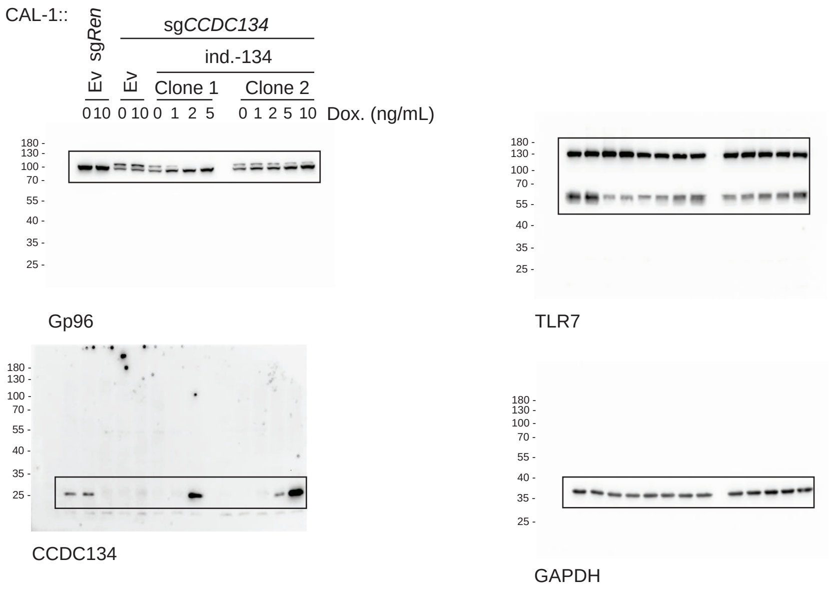

SourceDataF4B

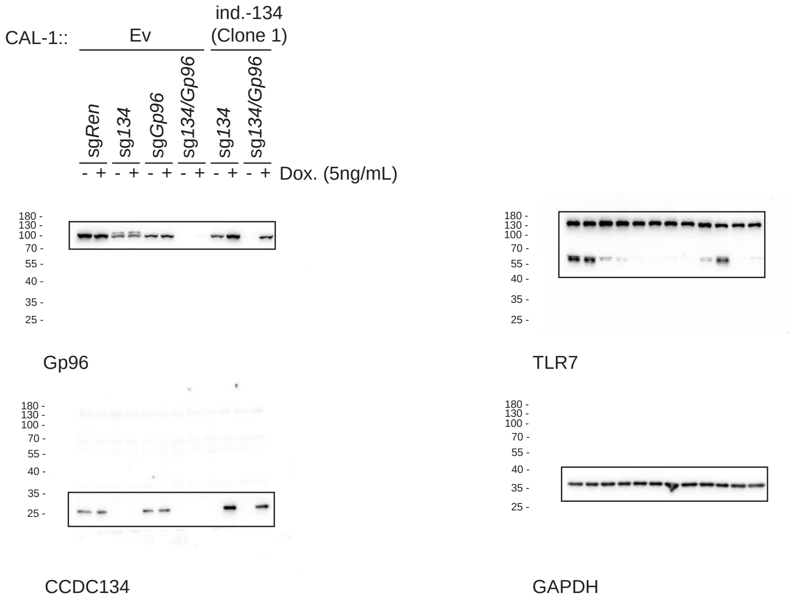

SourceDataF4D

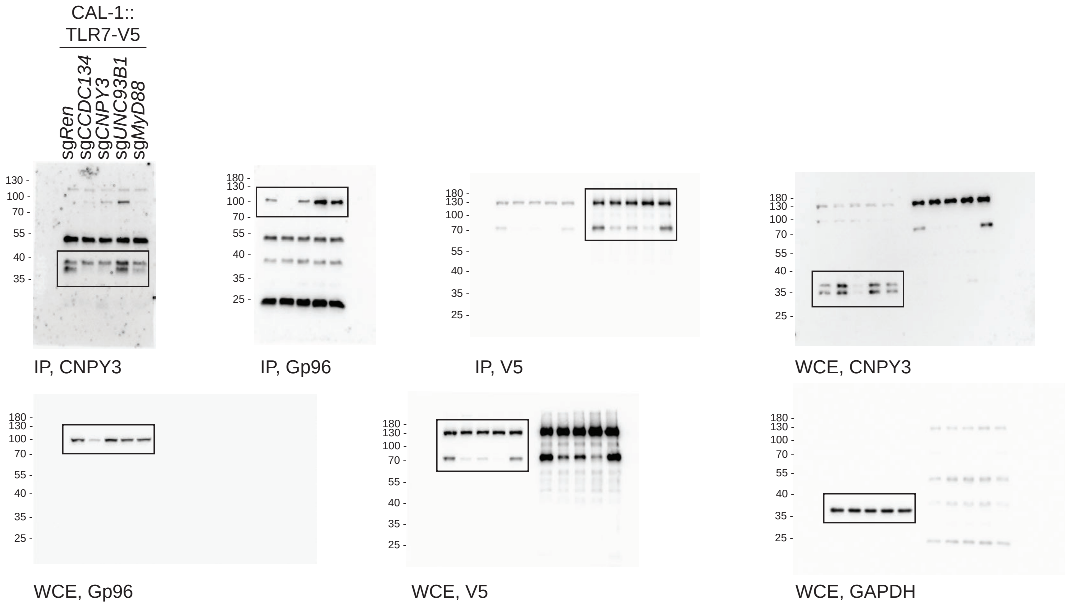

SourceDataF4E

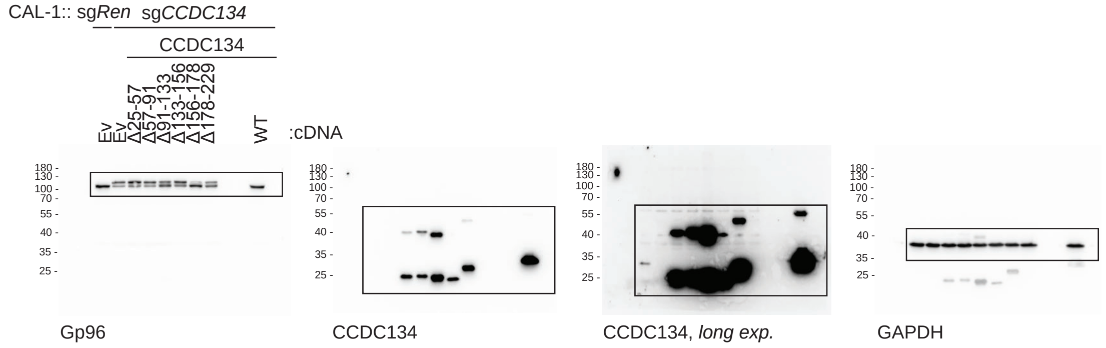

SourceDataF4G, left panel

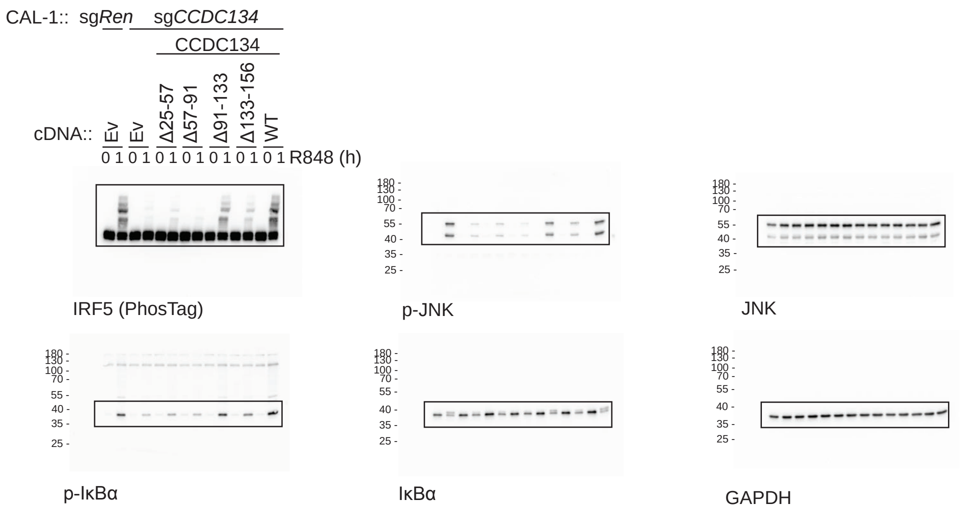

SourceDataF4G, right panel

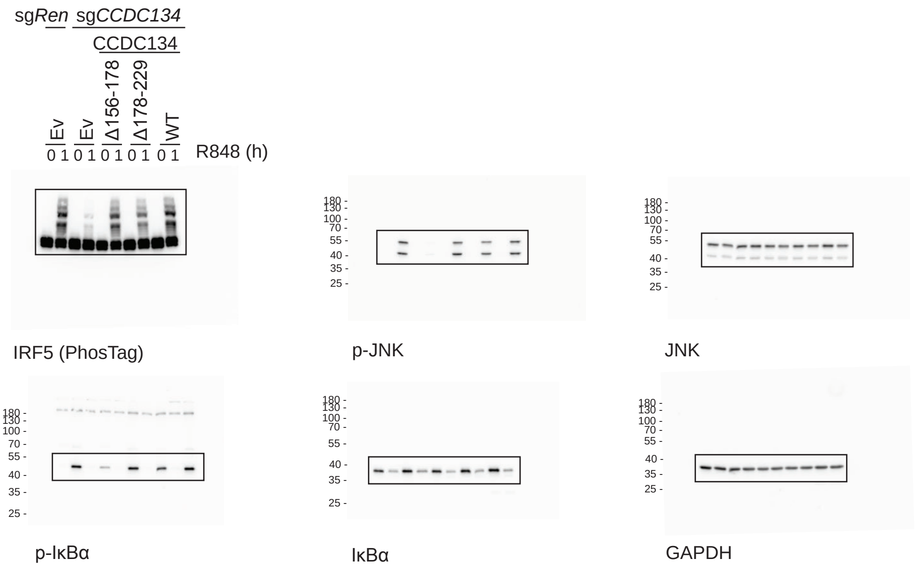

SourceDataF4H

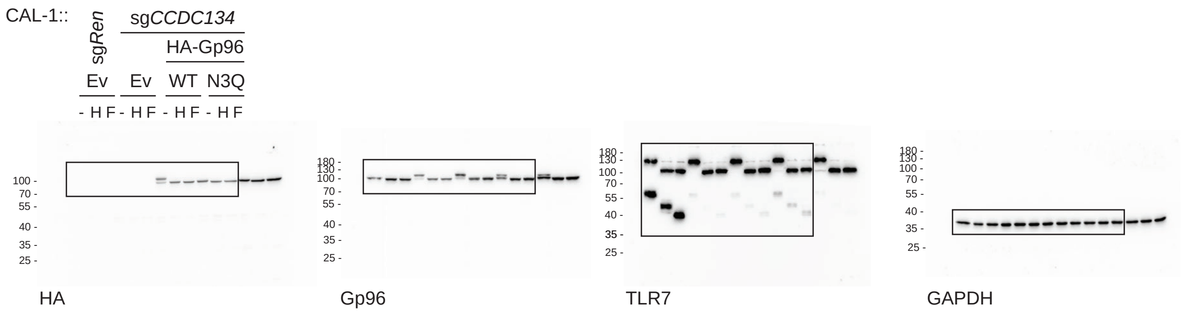

Supplement: SourceData F4 — is the source file for Fig. 4. [file jem_20240825_sourcedataf4.pdf]
