# Supplementary material for: CCDC134 controls TLR biogenesis through the ER chaperone Gp96
Source: J Exp Med. 2024 Dec 10;222(3):e20240825. doi: 10.1084/jem.20240825 (PMC11629888; doi:10.1084/jem.20240825)

SourceDataF5A

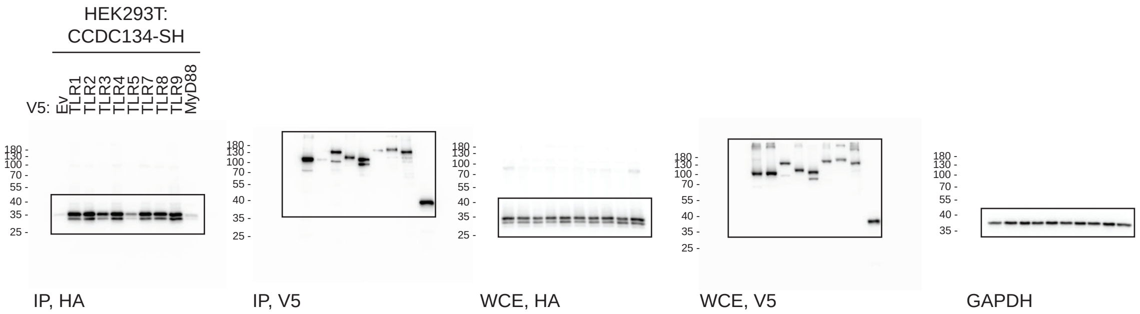

SourceDataF5B

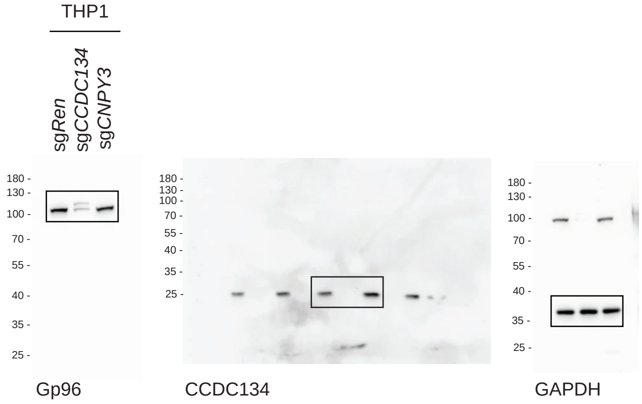

SourceDataF5C

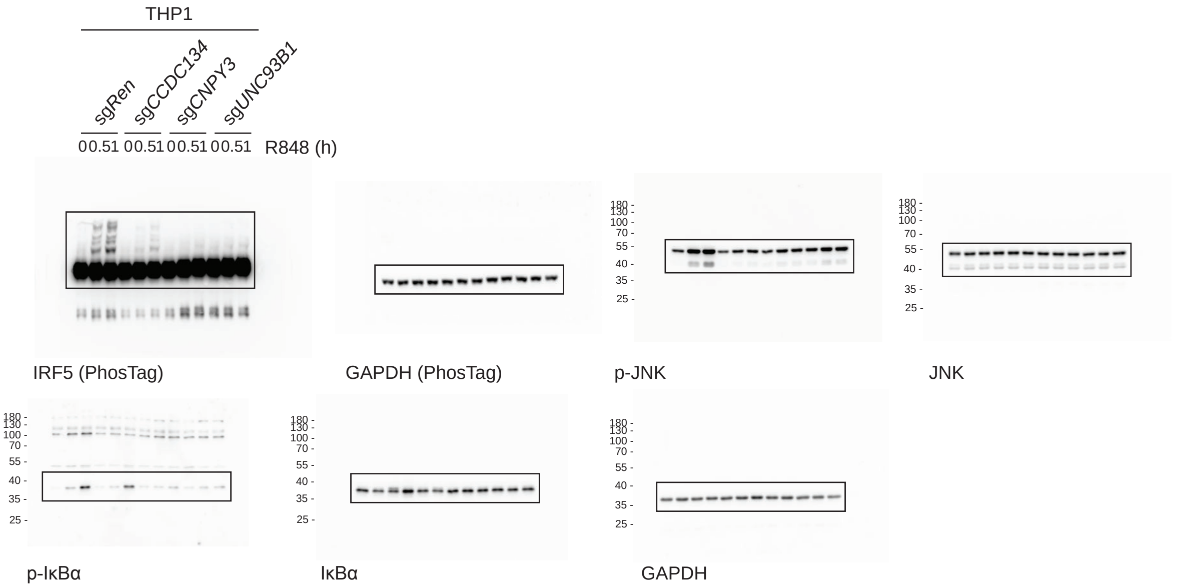

SourceDataF5E

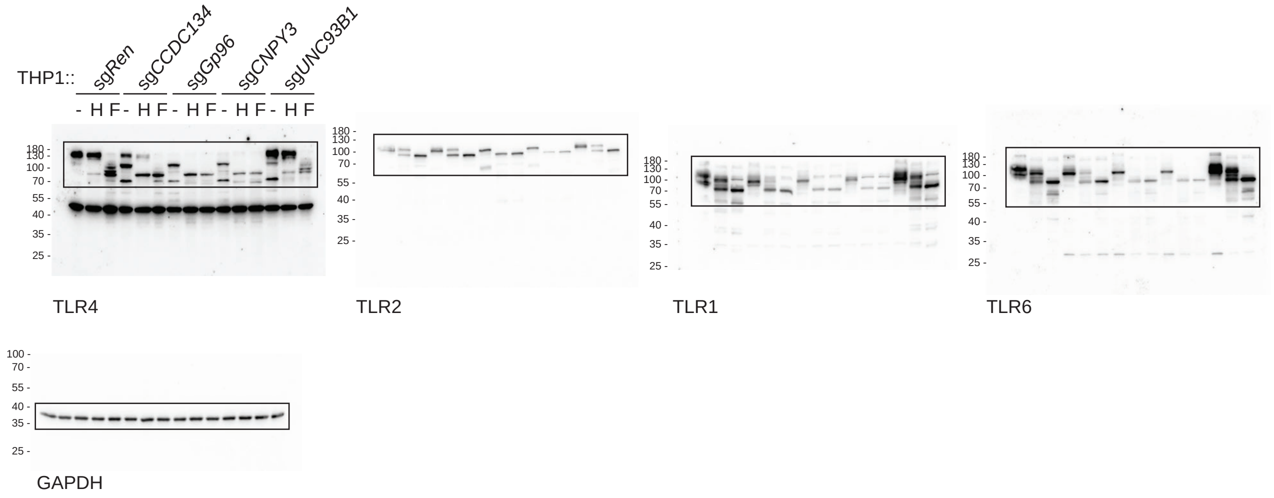

SourceDataF5G

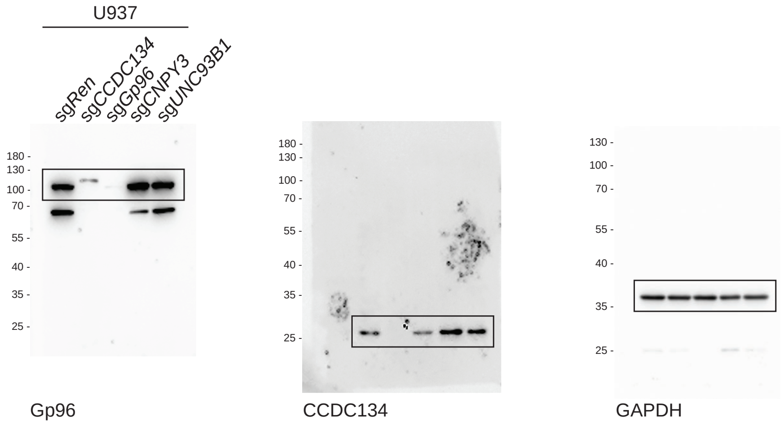

SourceDataF5I

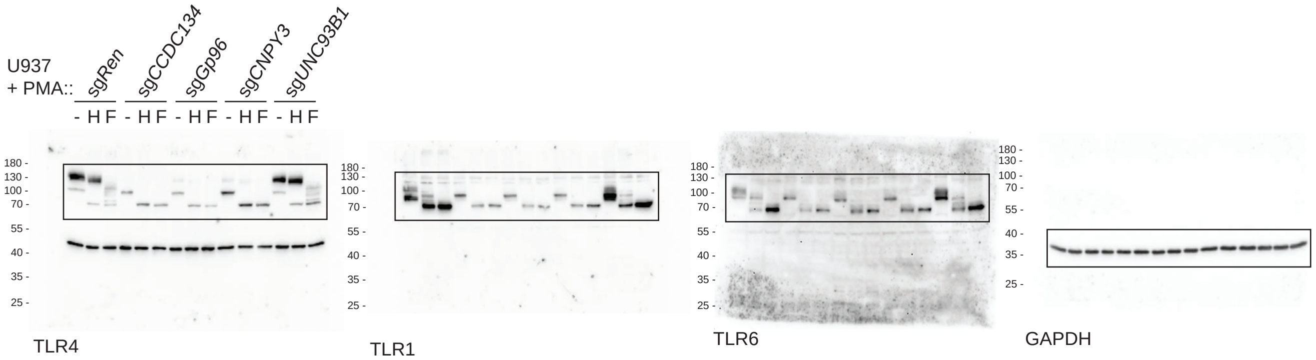

SourceDataF5J

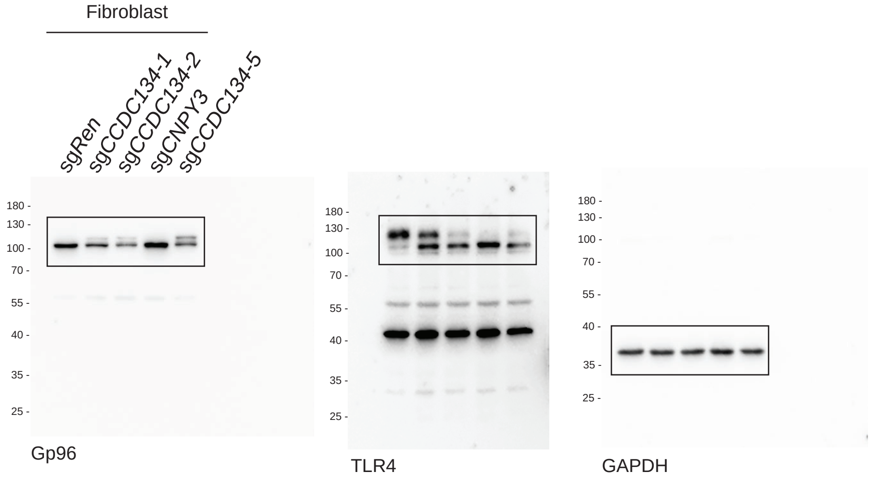

Supplement: SourceData F5 — is the source file for Fig. 5. [file jem_20240825_sourcedataf5.pdf]
