# Supplementary material for: CCDC134 controls TLR biogenesis through the ER chaperone Gp96
Source: J Exp Med. 2024 Dec 10;222(3):e20240825. doi: 10.1084/jem.20240825 (PMC11629888; doi:10.1084/jem.20240825)

SourceDataFS2D

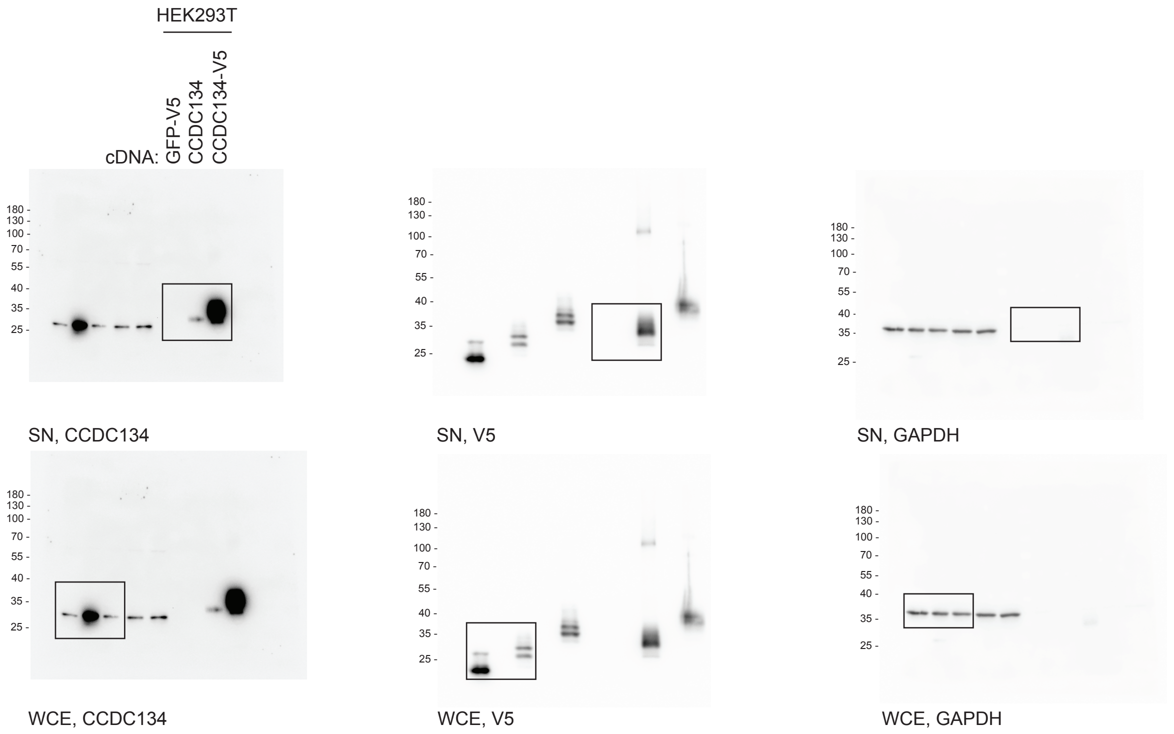

SourceDataFS2E

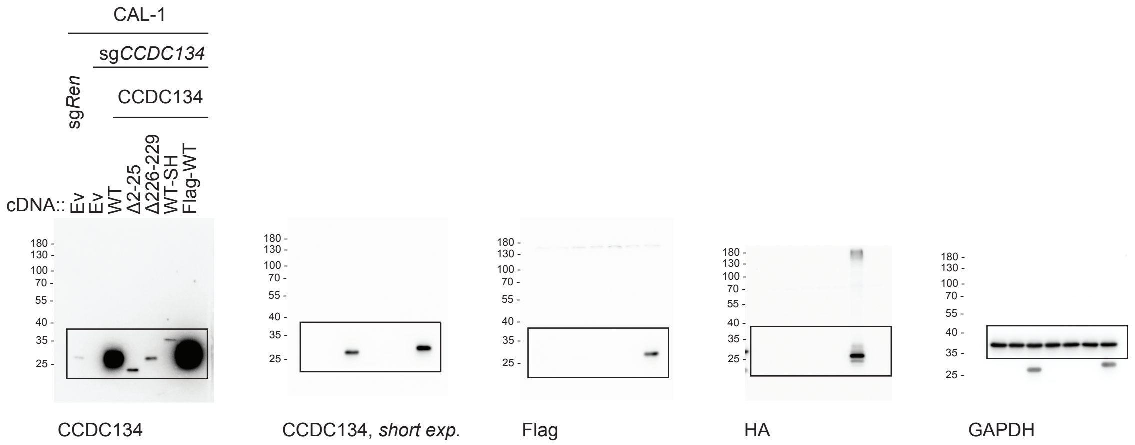

SourceDataFS2F, left panel

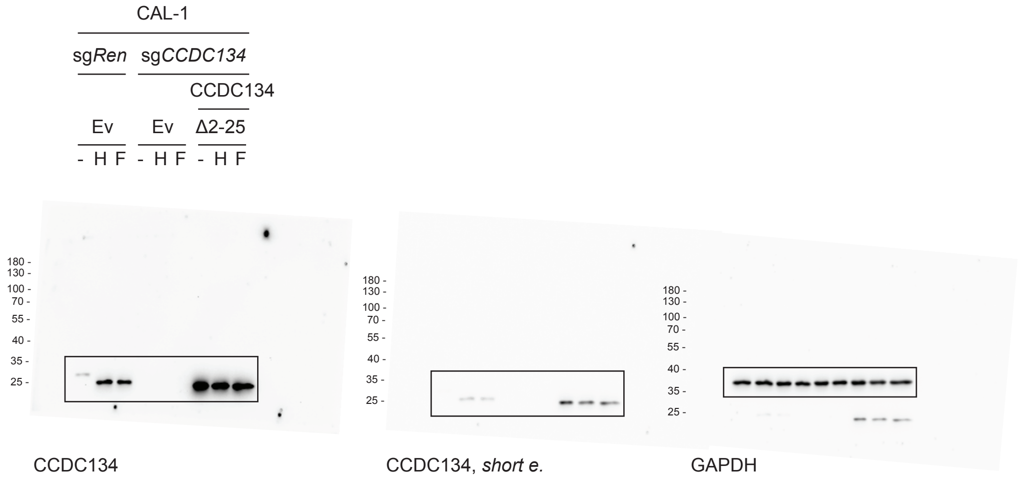

SourceDataFS2F, rigtht panel

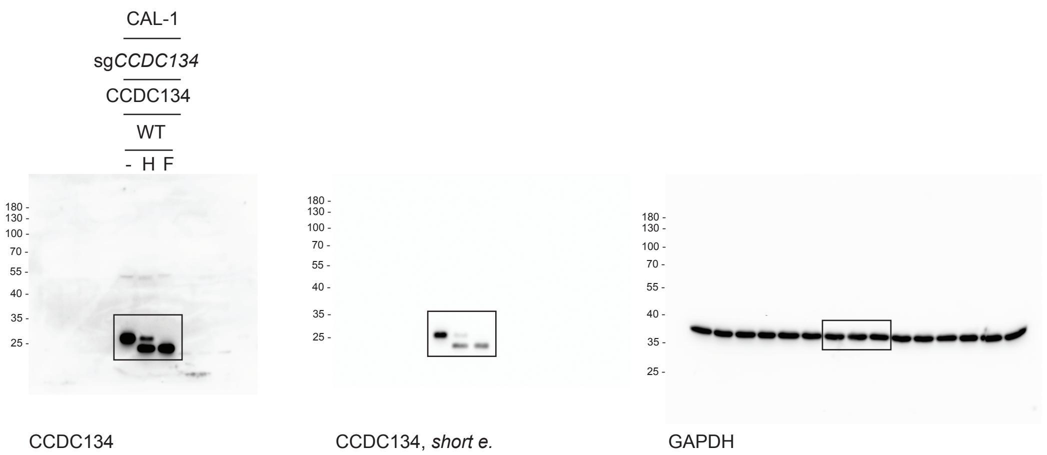

SourceDataFS2G

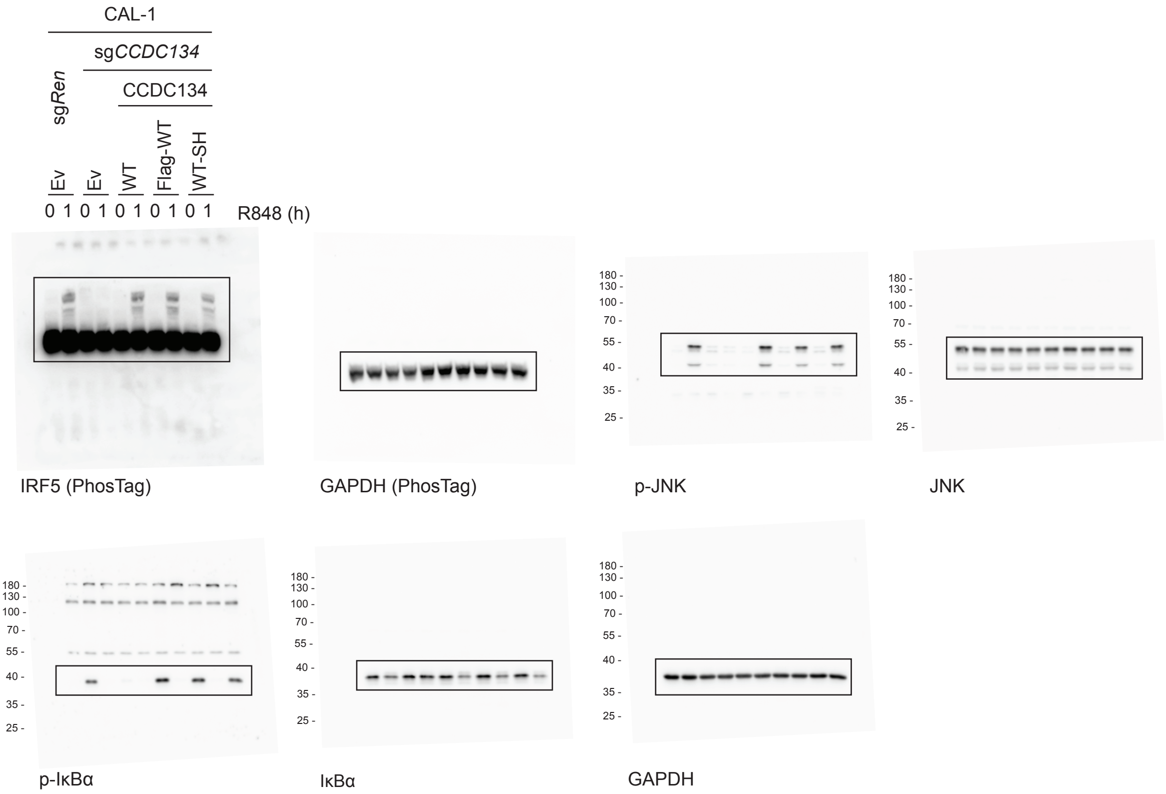

SourceDataFS2H

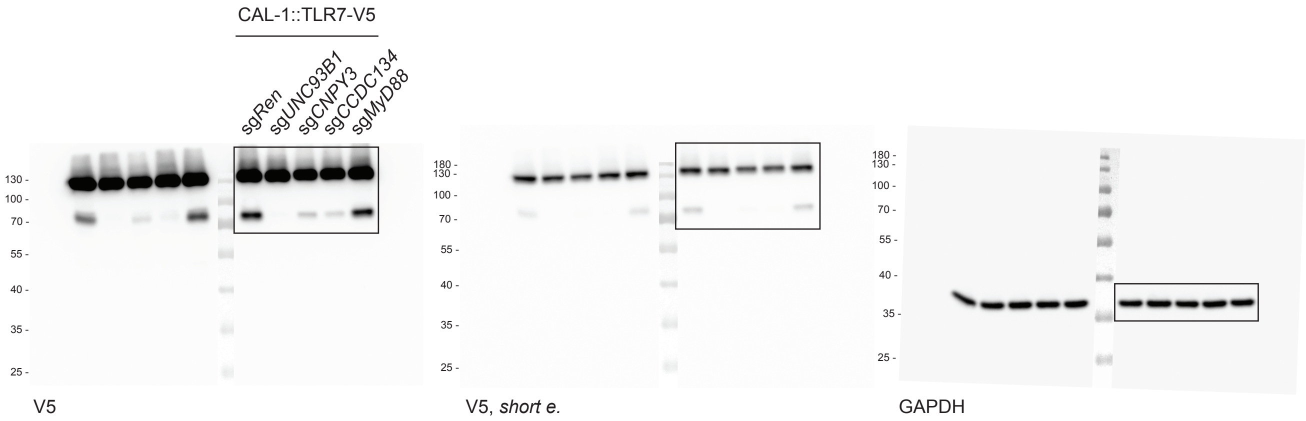

SourceDataFS2I

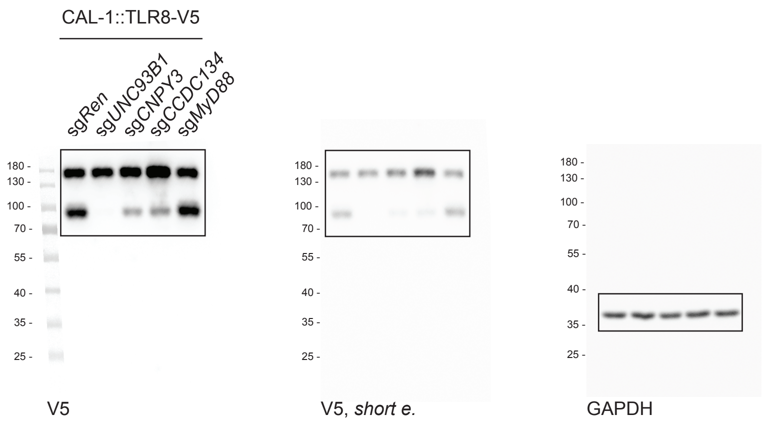

SourceDataFS2J

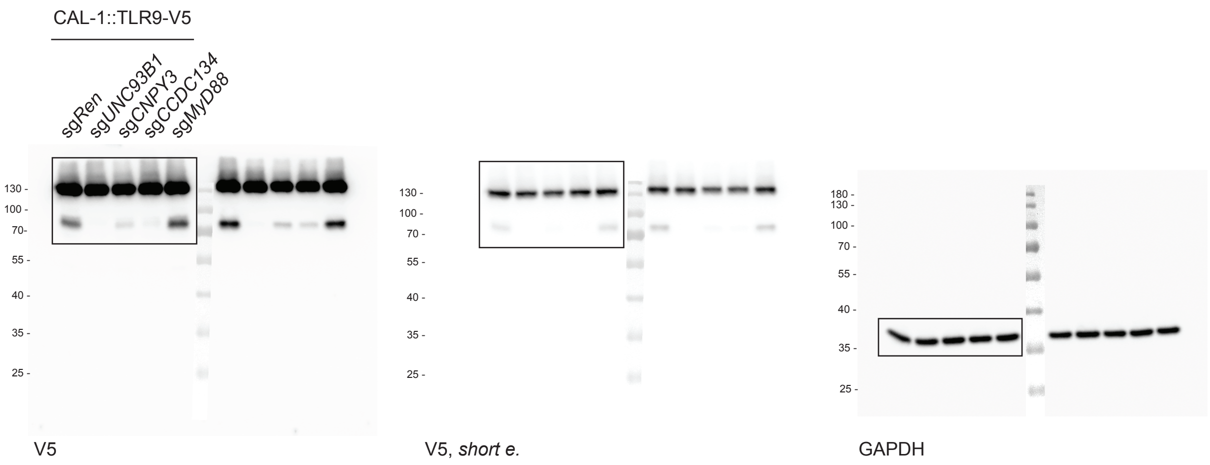

Supplement: SourceData FS2 — is the source file for Fig. S2. [file jem_20240825_sourcedatafs2.pdf]
